# The genomic basis of adaptive leaf variation in the Galápagos giant daisies

**DOI:** 10.1038/s41467-026-71865-3

**Published:** 2026-04-16

**Authors:** Vanessa C. Bieker, Siyu Li, José Cerca, Paul Battlay, Mohsen Falahati Anbaran, Amit Sharma, Patricia Jaramillo Díaz, Mario Fernández-Mazuecos, Jazmín Ramos-Madrigal, Sarah L. F. Martin, Luisa Santos-Bay, Gitte Petersen, Ole Seberg, Pablo Vargas, Rasmus Nielsen, M. Thomas P. Gilbert, Gonzalo Rivas-Torres, James Leebens-Mack, Loren H. Rieseberg, Lene R. Nielsen, Neelima Sinha, Michael D. Martin

**Affiliations:** 1 Department of Natural History, NTNU University Museum, Norwegian University of Science and Technology (NTNU), Trondheim, Norway; 2 Royal Botanic Gardens, Kew, Richmond, Surrey, United Kingdom; 3 Department of Plant Biology, University of California, Davis, Davis, CA USA; 4 Centre for Ecological and Evolutionary Synthesis (CEES), Department of Biosciences, University of Oslo, Oslo, Norway; 5 Department of Bioinformatics and Genetics, Swedish Museum of Natural History, Stockholm, Sweden; 6 SciLifeLab, Karolinska Institutet Science Park, Tomtebodavägen 23, Solna, Sweden; 7 School of Biological Sciences, Monash University, Melbourne, VIC Australia; 8 Department of Microbiology, University of Oslo (UiO), Oslo, Norway; 9 Charles Darwin Research Station, Charles Darwin Foundation, Santa Cruz, Galápagos Ecuador; 10 IUCN SSC Galápagos Plant Specialist Group, Puerto Ayora, Galápagos Ecuador; 11 Department of Botany and Plant Physiology, University of Málaga, Málaga, Spain; 12 Department of Biology (Botany), Facultad de Ciencias, Universidad Autónoma de Madrid, Madrid, Spain; 13 Centro de Investigación en Biodiversidad y Cambio Global, Universidad Autónoma de Madrid (CIBC-UAM), Madrid, Spain; 14 Department of Biodiversity and Conservation, Real Jardín Botánico (RJB), CSIC, Madrid, Spain; 15 Center for Evolutionary Hologenomics, The Globe Institute, University of Copenhagen, Copenhagen, Denmark; 16 Department of Ecology, Environment and Plant Sciences, Stockholm University, Stockholm, Sweden; 17 Botanical Garden, Natural History Museum of Denmark, University of Copenhagen, Copenhagen, Denmark; 18 Center for Theoretical Evolutionary Genomics, Department of Integrative Biology, University of California, Berkeley, CA USA; 19 Universidad San Francisco de Quito USFQ, Galápagos Science Center, San Cristóbal, Galápagos Ecuador; 20 Adjunct Faculty, University of North Carolina-UNC Chapel Hill, Chapel Hill, NC USA; 21 Department of Plant Biology, University of Georgia, Athens, GA USA; 22 Department of Botany and Biodiversity Research Centre, University of British Columbia, Vancouver, BC Canada; 23 Department of Geosciences and Natural Resource Management, University of Copenhagen, Rolighedsvej 23, Frederiksberg, Denmark

**Keywords:** Population genetics, Evolutionary genetics, Gene regulatory networks, Natural variation in plants

## Abstract

*Scalesia* (Asteraceae) is the largest endemic plant genus of the Galápagos archipelago and an example of adaptive radiation. While *Scalesia* species are highly varied in habit and morphology, most remarkable is their variety of leaf shapes, especially in the differential presence of leaf lobing/serration, a derived trait that evolved multiple times as a likely adaptation to the islands’ hot and dry equatorial climate. Using population-level genomic data from 396 individuals representing all 15 recognized *Scalesia* species, we characterize this young radiation (around 1 million years ago), and reveal that their substantial morphological divergence and ecological specialization are primarily based on shared genetic variation. To further elucidate the repeated adaptive evolution of leaf lobing in *Scalesia*, we integrate genomic and leaf morphometric data, with transcriptomes from different developmental stages, and conclude that leaf lobing evolved through diversifying selection. Natural selection occurs independently on different regulators in the pathway controlling development of adaxial-abaxial leaf polarity, highlighting the importance of the founder populations’ high genetic diversity maintained via allopolyploidy. Finally, our findings have implications for the conservation of *Scalesia*’s threatened biodiversity, as unexpectedly high intra-specific genetic structure and long-term isolation among populations indicate widespread nascent speciation.

## Abstract (web summary)

The Scalesia plant radiation displays striking leaf-shape variation within Galápagos microclimates. Genome-wide analyses shows that lobed leaves evolved repeatedly through diversifying selection on diverse developmental regulators.

## Introduction

Adaptive radiation has contributed substantially to the study of evolutionary biology. Such radiations have produced stunning diversity and extreme phenotypes across a variety of ecological niches. Species diversification during radiation is typically a product of increased speciation rates, possibly in combination with decreased extinction rates^1,2^. Adaptive radiations are driven by natural selection acting on trait variation, leading to ecological and phenotypic diversification^1,3–5^. Prominent instances of adaptive radiation include the cichlid fishes of East Africa^6^, the silversword plants of Hawai’i^7^, and the Caribbean anole lizards^8^.

Among oceanic archipelagos, the Galápagos archipelago has been a hotspot for at least ten adaptive radiations^9^ and holds an important status in the history of evolutionary thinking. Famously, Charles Darwin’s retrospective consideration of the distributions and phenotypic diversity of Galápagos finches, which show substantial morphological variation in beak size and shape as an adaptation to different feeding habits^10^, provided evidence of the special nature of Galápagos radiations and for his nascent theory of evolution by natural selection^11^. Sometimes referred to as the “Darwin’s finches of the plant world”^12,13^, the plant genus *Scalesia* (Asteraceae) is arguably an even more spectacular adaptive radiation^14,15^. Darwin’s own personal collections of *Scalesia* specimens during his 1832–1836 voyage^12,16^ led to botanist J. D. Hooker’s early observation of their close relations to South American Asteraceae^17^. With 15 extant species currently accepted, *Scalesia* is the largest endemic plant genus of the Galápagos^18^ where it has radiated to produce extreme diversity in life form and habit. *Scalesia* are fast-growing woody plants that reach maturity early and generally have a short life expectancy of about 14 years^19^. Three species are remarkable “daisy trees” that form dense highland forests on the larger islands and thus play a fundamental role in Galápagos ecology^20^.

Although the *Scalesia* lineage split from its closest mainland relatives about 3 Ma, diversification of the extant species appears to have occurred in the last million years, after the emergence of most islands of the Galápagos^13^. Today, *Scalesia* species are distributed across the archipelago on eleven main islands, with several islands containing more than one species^13–15,18^. The older and larger islands tend to host more species, with up to six species co-occurring on the island of Santa Cruz. However, even when several species occur on the same island, their distributions are mostly allopatric^18^. Indeed, most speciation events may have occurred within islands rather than between islands^13^.

**Fig. 1:**
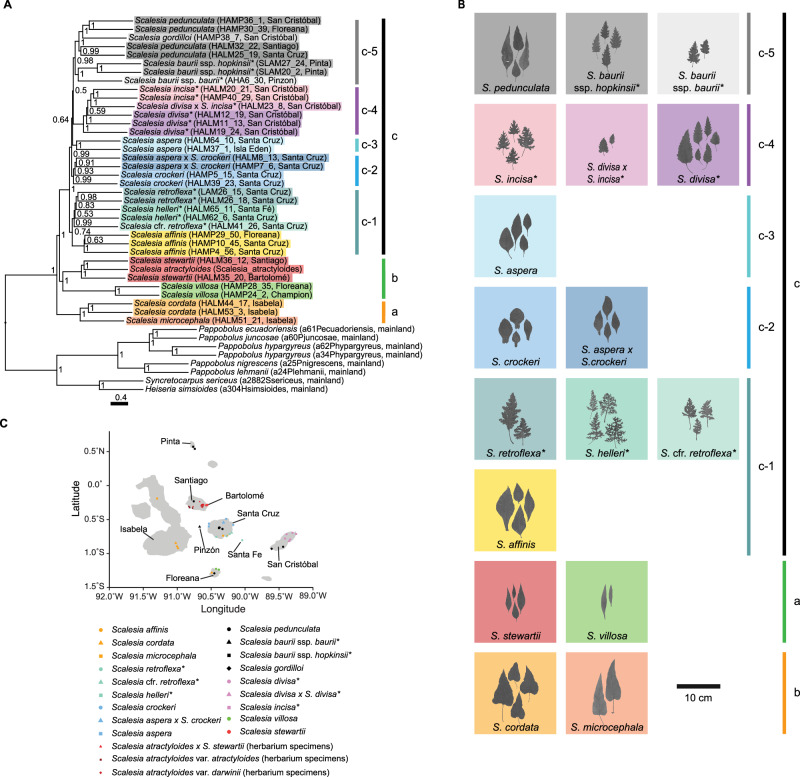
Phylogeny and overview of sampled *Scalesia* species. **A** Multi-species coalescent phylogeny based on samples with sequencing depth samples >8X. Numbers on nodes indicate posterior probability, and internal branch lengths are in coalescent units (*N*_*e*_ generations). Terminal branch lengths are set to 1 in the phylogeny and tips are labeled with species names and identifiers for the individual plants, with island names given in parentheses. Tip labels are colored by species. Vertical bars and labels indicate clades (a, b, c) and subclades (c-1, c-2, c-3, c-4, c-5). **B** Examples of leaf scans of *Scalesia* species included in the leaf morphology analysis. **C** Map with sampling locations for populations and herbarium samples included in the genetic analysis. For better visibility, maps of sampling location per species can be found in Figs. S2–8. Source data are provided on Dryad (10.5061/dryad.8gtht76rh). **A**–**C** *Scalesia* species with a lobed leaf phenotype are marked with an asterisk.

The diversification of *Scalesia* into the varied but predominantly open, rocky, and dry environments of the Galápagos archipelago provides a textbook example of a plant adaptive radiation^3,14,21^. Although it is relatively young, the genus *Scalesia* shows a remarkable phenotypic diversity in habit (shrubs and trees), inflorescence (capitulum), fruit (achene) types^22^, and especially leaf morphology^13,14^, which is not seen in its closest mainland relatives^23,24^. Much attention has been paid to the variability in the shapes and sizes of *Scalesia* leaves, which range from small and highly dissected, to large and entire (Fig. 1B). Among other traits, plant leaf size has been correlated with the environment, with smaller, more lobed leaves often produced by species that occupy hot and arid habitats^25–29^. In *Scalesia*, the occurrence of lobed-leaf phenotypes throughout the phylogeny is a derived trait thought to be an adaptation to dry and warm climate, as lobed leaves reduce transpiration and increase the rate of heat dissipation^13,14,30^. Further adaptive significance of such leaf lobing may arise from reduced hydraulic resistance and improved water balance under arid conditions, reducing self-shading and more efficiently covering the available area^31^, as well as reducing the dependence of heat dissipation on leaf orientation^30,32,33^. It has been shown that in the *Oreinotinus* plant radiation that different leaf ecomorphs evolved in parallel and that disparate leaf forms differ in their climate niche^34^. Indeed, all *Scalesia* species with lobed leaf phenotypes are restricted to hot and dry climate zones^14^, excepting only *S. baurii* which may also occur in moist forest zones^13,35^.

While a key feature of adaptive radiation is that traits exhibiting exceptional variability have been subject to natural selection, leading to adaptation^3^, this is typically challenging to demonstrate. Although it is relatively well accepted that such radiation can be driven by divergent natural selection for adaptation to different environments^3^, and that adaptive evolution is negatively correlated with population size and is more common in rapid radiations than in slower evolving lineages^36,37^, the genetic basis underlying this adaptive evolution is much less well understood. For example, selection can act on genetic variants from different sources: ancestral variation inherited from the common ancestor^38^, de novo mutations that arose during diversification^39^, or introgressed variants that are exchanged via interspecific hybridization^40^. But only in relatively few cases have genome sequencing approaches been applied to investigate the biological mechanisms of adaptive radiation^9^.

A recent chromosome-level nuclear genome assembly for *Scalesia atractyloides* provided a basis for exploring the expansion of specific gene families within the daisy tree lineage and the role of the recent ploidy shift in *Scalesia*’s rapid evolution^41^. Here, we leverage this high-quality reference genome to apply phylogenomic and population genomic approaches using whole-genome re-sequencing data of 396 accessions representing all 15 currently accepted *Scalesia* species. We combine the genetic data with leaf morphometric analysis and transcriptomic data to characterize selection on specific genes underlying the adaptive variation in leaf lobedness, a key phenotype in this insular plant radiation.

## Results and discussion

We present a population-level dataset of 401 whole genomes that represent the 15 species in genus *Scalesia*, including both subspecies of *S. baurii*, two varieties of *S. atractyloides*, and additionally four putative hybrid populations (Fig. 1C and Figs. S2–8). Mapping rates against the 3.2-Gbp *S. atractyloides* reference genome^41^ ranged between 83 and 99% (mean: 98.9%, SD: 0.0159), achieving a mean final sequencing depth of 4.8X (SD: 3.24, Fig. S1). One sample was excluded from the final dataset due to too-low sequencing depth, and four samples were excluded due to being first- or second-degree relatives of other samples in the dataset, resulting in a final analysis dataset of 396 genomes comprising five to ten individuals per population and one to eight populations per species. For most of the populations, one sample was sequenced to >8X sequencing depth (Supplementary Data 1).

### Rapid diversification, long-term isolation, and ongoing speciation

**Fig. 2:**
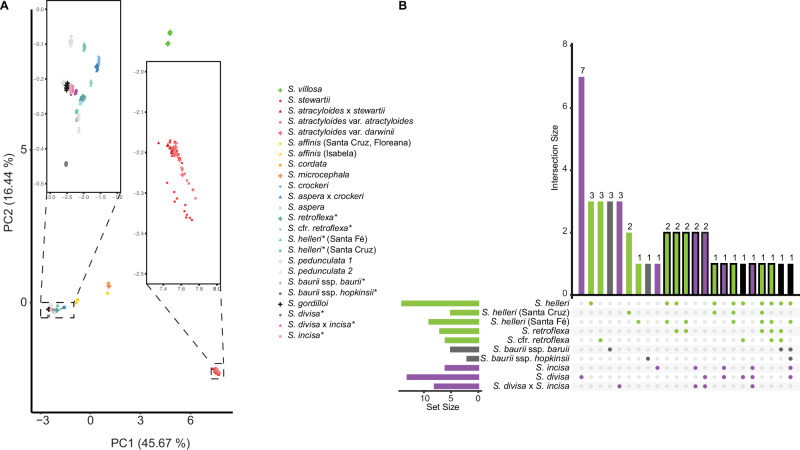
Genetic structure and sharing of putatively selected leaf-gene variation in the *Scalesia* radiation. **A** PCA of *Scalesia* species based on genotype likelihoods. Samples are colored based on species. *Scalesia* species with a lobed leaf phenotype are marked with an asterisk. **B** UpSet plot of sharing of putatively selected leaf genes between lobed *Scalesia* species. Top: intersect size. Colored bars with black outline show intersections within subclades with green: clade c-1 (*S. helleri*, *S. retroflexa*, and *S*. cfr. *retroflexa*); gray: clade c-5 (*S. baurii* ssp. *baurii, S. baurii* ssp. *hopkinsii*); purple: clade c-4 (*S. incisa, S. divisa, S. divisa* x *incisa*); black: intersection between different subclades. Numbers above each bar indicate the intersection size. Bottom left bars: Number of putatively selected leaf genes (set size) within each lobed species. Bottom right: Matrix showing the intersection with colored circles indicating which species intersect. If only one set is colored, it indicates the genes unique to the species. Circles are colored based on subclade. Source data are provided on Dryad (10.5061/dryad.8gtht76rh).

Adaptive radiation is the rapid diversification of a lineage into different species whose phenotypic diversity is associated with ecological characteristics^3^. Parsing of the results from our phylogenetic analysis using ASTRAL reveals many short internode branches on the spine of clade *c*, which includes, among unlobed species, all species with lobed leaves (Fig. 1A), suggesting some combination of increased speciation rates and decreased extinction rates relative to *Scalesia*’s early evolution. Thus, the tree suggests that net diversification increased with the evolution of lobed leaves some time after colonization of the Galápagos. Despite the large interspecific morphological variation between species within clade *c*, they cluster closely together on the PCA (Fig. 2A) and their pairwise *F*_*ST*_ and *d*_*XY*_ values are frequently below 0.2 and 0.041, respectively (Fig. S9). Such *F*_*ST*_ values are typically considered moderate to high for differentiated populations within species^42^, indicating that individual *Scalesia* species have not yet managed to generate substantial genetic differentiation despite their success in adapting to various environments. For comparisons between *Scalesia* species in clades *a* and *b*, relative to clade c species, *F*_*ST*_ and *d*_*XY*_ values are generally higher (*F*_*ST*_ > 0.25, *d*_*XY*_ > 0.042). Moreover, samples in clade a and b are separated from clade *c* species on the PCA (Fig. 2A). Genome-wide Tajima’s D ranges between −0.9 and 1.3, with most species showing a positive value, indicating recent population decline (Figs. S10 and S11). In addition, our PSMC reconstructions of effective population size (*N*_*e*_) dynamics using only samples with sequencing depth above 12 after MAPQ30 filtering of 35 *Scalesia* populations show only recent divergence of extant populations (Fig. S12). Prior to ~1.4 Ma, all species share a similar demographic trajectory, indicating that they may not have yet begun to diversify into species. Between 0.2 Ma and 1 Ma, population sizes of extant *Scalesia* taxa increased and began to differ from one another, consistent with previous phylogeny-based estimates of the timing of *Scalesia* diversification^13^. Together, these results indicate that *Scalesia* indeed underwent rapid radiation, with an especially rapid differentiation of clade *c* that spawned three origins of leaf lobedness (Fig. 1).

Despite the rapid diversification and young age of the genus, we detected only limited gene flow between extant *Scalesia* species. This is indicated by most species forming distinct clusters in the PCA (Fig. 2A) and being fully assigned to unique genetic clusters in the admixture analysis (Fig. S13). However, not all species show a clear separation based on the PCA and admixture analysis (see Supplementary Note 1). Species occurring on several islands often show strong population differentiation and genetic structure (Table 1 and Fig. S13), indicating limited gene flow between islands. In several cases, intra-specific differences in *N*_*e*_ were notably already present early on (Fig. S12). Moreover, our *F*_branch_ analysis shows that allele sharing mostly occurs from ancestral lineages to extant species and is higher within clade *c* than within or between the other clades (Fig. S14), congruent with incomplete lineage sorting during the rapid diversification^43^ of clade *c*. Low gene flow between species, even when occurring on the same island, may be due to their nearly allopatric distribution^18^ in combination with poor adaptation for long-distance dispersal in *Scalesia*^14^. In the case of occasional successful long-distance dispersal or the long-distance movement of pollen, other factors may prevent hybridization. As *Scalesia* species occupy different niches such as dry lowlands or humid highlands^13^ and have a nearly allopatric distribution^18^, hybrids may experience outbreeding depression and thus be unable to establish successfully. However, successful hybridization does occur within some islands (see Supplementary Note 1), and incomplete reproductive barriers have been found between some *Scalesia* species^44^.

**Table 1:** *F*_*ST*_ between populations of species found on different islands

Species	Island 1	Island 2	*F*_*ST*_ (weighted)
*S. aspera*	Isla Eden	Santa Cruz	0.239197
*S. stewartii*	Bartolomé	Santiago	0.063696
*S. villosa*	Champion	Floreana	0.063906
*S. affinis*	Isabela	Santa Cruz	0.244783
*S. affinis*	Isabela	Floreana	0.274747
*S. affinis*	Santa Cruz	Floreana	0.081650
*S. pedunculata*	Santa Cruz	Santiago	0.099942
*S. pedunculata*	Santa Cruz	Floreana	0.112116
*S. pedunculata*	Santa Cruz	San Cristobal	0.162225
*S. pedunculata*	Santiago	Floreana	0.181842
*S. pedunculata*	Santiago	San Cristobal	0.221148
*S. pedunculata*	Floreana	San Cristobal	0.119267
*S. helleri*	Santa Cruz	Santa Fe	0.204068

We also found multiple lines of evidence suggesting a need for taxonomic revision to better reflect the systematic relationships within this emblematic group of plants (see Supplementary Notes 1–3). Populations from several *Scalesia* species are differentiated (Table 1 and Fig. S13) and do not always form monophyletic groups (Fig. 1A), indicating that each should be regarded as a different taxonomic unit, or even as different species, despite their morphological similarities^14,15^ (see Supplementary Notes2–3). In most cases, conspecific *Scalesia* populations have already begun to follow separate evolutionary trajectories, often for more than 50k years (Fig. S12). This new understanding of the high degree of intra-specific genetic structure in *Scalesia* has profound implications for conservation of its threatened biodiversity and for management of the most threatened populations^45^.

### Leaf morphometrics delineate the lobed phenotype

**Fig. 3:**
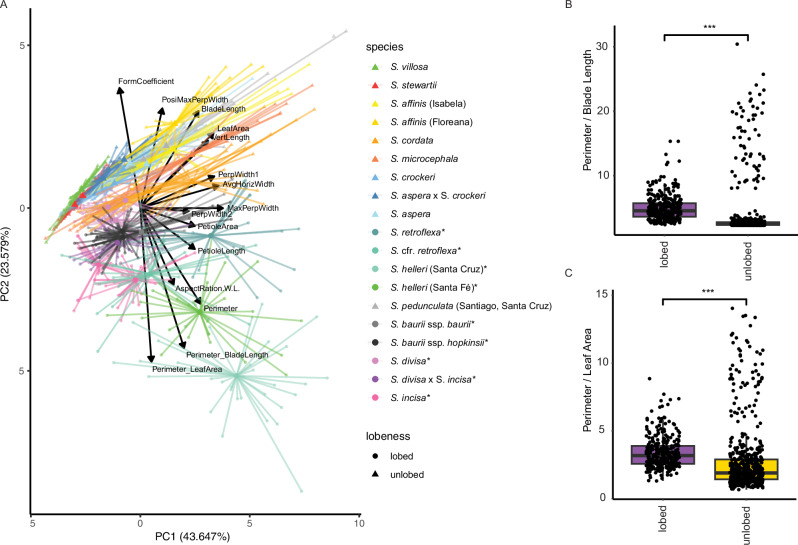
Morphometric discrimination of *Scalesia* leaf shapes. **A** PCA of morphological characters. The mean value for each population is plotted in dark and the individual measures are plotted in light color and connected to the mean value with a line. Values are colored based on species assignment and shaped based on lobeness. Lobed species are marked with an asterisk in the legend. The biplot was scaled by a factor of 10 to increase visibility **B** Boxplot of perimeter/blade length. **C** Boxplot of perimeter/leaf area. **B**,** C** Boxplot for lobed species in purple (*n* = 311 measurements) and unlobed in yellow (*n* = 560 measurements). Box limits represent the 25th and 75th percentiles with the median shown by a black line. Individual data points are plotted over the box. Significance was estimated with a two-sided Wilcoxon signed-rank test in R with the *p* value displayed as *** = 0.001, ** = 0.01, * = 0.05. Source data are provided on Dryad (10.5061/dryad.8gtht76rh).

To quantitatively assess the leaf morphological variation within the genus, 16 different leaf morphological traits were measured from pressed leaf samples of one to three populations per species and ten to twelve individuals per population (Supplementary Data 2 and 3). A PCA of these leaf morphometric data (Fig. 3A) shows considerable overlap between species and often a high within-species variation, with the average distance to the species mean on PC1 and PC2 ranging from 0.61 (SD: 0.49) for *S. stewartii* to 3.13 (SD: 1.88) for *S. microcephala*. Species with a lobed leaf phenotype segregate from unlobed species on PC2, with most of the variance on this axis being explained by the metrics *Perimeter*, *Perimeter/LeafArea*, and *Perimeter/BladeLength* (Fig. 3). We defined all taxa with a mean PC2 < 0 as lobed (*S. retroflexa*, *S. helleri*, *S*. cfr. *retroflexa*, *S. baurii* ssp. *baurii*, *S. baurii* ssp. *hopkinsii*, *S. incisa*, *S. divisa, S. divisa x S. incisa*), with *S. divisa* showing the least extreme lobed phenotype and *S. helleri* showing the most extreme lobed phenotype (see also Fig. 1B). This delineation of lobed species includes all species that were previously non-quantitatively described as “distinctly lobed” or “slightly lobed”^13^. The two species not included in our analysis (*S. atractyloides*, *S. gordilloi*) were previously described as unlobed.

### Convergent evolution of lobed leaves

The derived lobed leaf phenotype only occurs in three subclades (c-1, c-4, c-5) within the rapidly radiated clade c of the *Scalesia* phylogeny (Fig. 1A). Introgression analysis based on *F*_*branch*_ statistics, however, shows no strong genome-wide signal of introgression between different lobed species (Fig. S14), in line with previous findings of three origins of the lobed phenotype using ancestral state reconstruction and *D*-statistics^13^. To infer which genes may underlie the lobed leaf phenotype and to understand whether different species utilized the same genes in their evolution of lobed leaves, we performed *F*_*ST*_ outlier analysis based on global *F*_*ST*_ measures between the different lobed and the closest related unlobed species (Fig. S9). First, we scanned the genome for windows of extreme outlier pairwise genetic differentiation as measured by *F*_*ST*_ (*ZF*_*ST*_ > 4) between lobed and unlobed species (Figs. S15–S24). We further filtered these outlier windows for those containing genes previously implicated in leaf development processes (Supplementary Data 10 and 11) and showing evidence of selection in the lobed species, as defined by a negative Fay & Wu’s *H*^46^. A total of 78 outlier windows (between two and 13 per species) were identified (Supplementary Data 4), containing 43 unique *Scalesia* genes corresponding to 38 unique *Arabidopsis thaliana* genes (Supplementary Data 5). The putatively selected genes are enriched for GO terms associated with gibberellin hormones (e.g., GO:0009739 and GO:0009686) as well as flower development (GO:0009908) (Fig. S25). Most (56%) of the putatively selected leaf genes are only found in one lobed species, and no gene is shared by all lobed species (Fig. 2C). Of those shared among two lobed species, the majority (16/19) are shared by species in the same subclade (e.g., *S. retroflexa* and *S. helleri*), indicating that these genes may already have been under selection in their common ancestor. We chose the window-based *F*_*ST*_-outlier analysis due to the low sequencing depth of the data, as it uses genotype likelihoods and is thus appropriate for low sequencing depth datasets^47^. However, as a window may contain several genes, the exact gene or mutation under selection cannot be identified. Future studies should further investigate the candidate genes under selection identified here using higher sequencing depth and functional genomics to further investigate the genomic basis of lobed leaf phenotypes in *Scalesia*.

In addition to the *F*_*ST*_ outlier analysis, we performed a GWAS using samples from closely related lobed and unlobed species of clade c, revealing a single outlier region on chromosome 29 (Figs. S26–S28). Outlier single-nucleotide polymorphisms (SNPs) within this region overlap with eleven genes (Supplementary Data 6), but only one of these genes (*ATEBP1*) is associated with leaf development, and another (*ARP4*) is involved in flowering (GO:0048574) and pollen cell differentiation (GO:0048235). The *Scalesia ARP4* homolog may play a role in the partially heterogamous flower morphology that is observed only in lobed *Scalesia* species^13^. In the *F*_*ST*_ outlier analysis, all lobed species have outlier windows on chromosome 29 (Figs. S15–24), but only within *S. divisa* do these outlier regions overlap with the leaf development-associated gene found in the GWAS. It should be noted, however, that even after accounting for population structure in the GWAS, elevated *p* values are found (lambda = 2.31, Fig. S27), likely because population structure strongly correlates with the lobed leaf phenotype. Together, our results indicate that the different species mostly use different genes to realize the lobed leaf phenotype and that this phenotype likely evolved in clade *c* through three episodes of convergent evolution. This is congruent with findings in the island-like cloud forest system of the rapidly diversified *Oreinotinus* clade (*Viburnum*) where similar leaf morphologies evolved in parallel in different regions, likely due to ecological adaptation^34^.

### Selection on leaf development regulatory networks

**Fig. 4:**
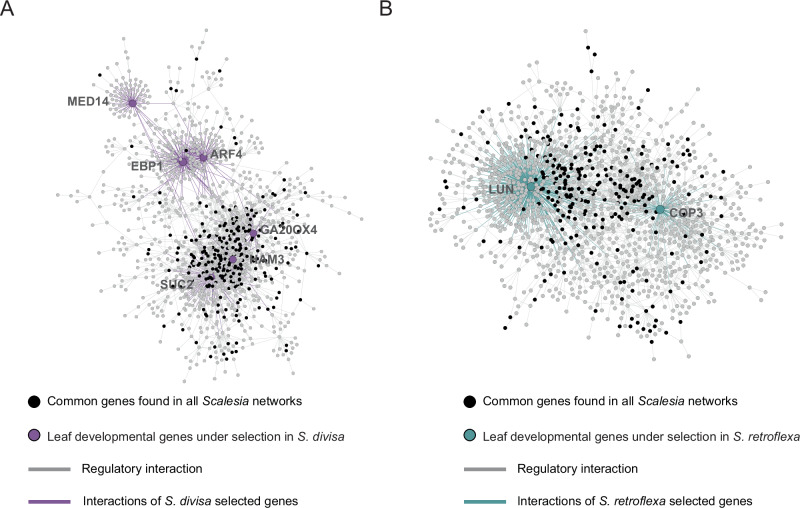
Leaf regulatory networks for lobed *Scalesia* species. Species-specific networks were constructed using known transcription factors overlaid with putatively selected leaf development genes under selection in the given species. **A** Overlaid network for *S. divisa*. **B** Overlaid network for *S. retroflexa*. Colored edges represent species-specific interactions from putatively selected genes connecting to the leaf developmental networks. Leaf developmental genes under selection are labeled with the *A. thaliana* ortholog gene symbol.

Subtle changes in gene expression during leaf development can lead to a remarkable diversity in the shapes of leaves^48^. We used transcriptomic data to investigate networks of gene expression during four leaf developmental stages in seven *Scalesia* species with lobed or unlobed leaves (Fig. 4 and Supplementary Data 7) (see Supplementary Note 4). Several of the genes putatively under selection in lobed-leaf *Scalesia* species are involved in the adaxial/abaxial leaf development regulatory pathway, which is known to control leaf shape in diverse plants^49,50^. For example, the gene *LEUNIG* (putatively under selection in *S. retroflexa*) acts as a co-repressor to regulate leaf blade outgrowth^51^, and *ARF4* (putatively under selection in *S. divisa*) determines adaxial tissue fate^52^ and influences leaf lobing^53^. In comparison to species lacking lobed leaves, these genes are not differentially expressed during leaf development (Fig. S29). However, the genes putatively under selection in lobed species are highly interconnected within the species-specific leaf developmental networks (Fig. 4). Although these genes function as hubs within these networks, they were not identified in an analysis of combined networks, indicating that the regulation of these genes may be temporal and not conserved across *Scalesia* species. Also, these genes are transcription factors, and their family members have been shown to exhibit redundant functions during leaf development. These results fit well with the proposed ‘omnigenic’ model^54^ that major changes in phenotype can be generated by modulating the multitude of peripheral genes in expression networks, rather than a few highly conserved core genes. Thus, each independent evolution of *Scalesia* leaf lobing has achieved species-specific modulations of the leaf-development transcriptomic network by co-opting different genes in the pathway determining leaves’ flattened horizontal orientation.

### Leaf shape as an ecological innovation in a plant radiation

*Scalesia* species inhabit diverse environments from dry lowlands to humid highlands^14,35^ (see Supplementary Note 5). Their lobed leaves are thought to be an adaptation to dry, warm climates, such as those at low elevations on the Galápagos archipelago^14^, in that they can reduce water loss through transpiration and increase heat dissipation^14,30^, although not all species in dry regions produce lobed leaves^13^. Indeed, the GO enrichment analysis of putatively selected genes that were previously identified to be associated with leaf development^55–57^ (Supplementary Data 10) showed an enrichment for involvement in heat response (GO:1900036) (Fig. S25). Moreover, all but one lobed population (SLAM27, transition zone, *S. baurii* ssp. *hopkinsii*) in this study inhabit dry zones, whereas the unlobed species are found in the dry, transition, and humid zones (Supplementary Data 1). The other *S. baurii* ssp. *hopkinsii* population included in our analyses (SLAM20) occurs in a dry zone on the same island (Pinta). Thus, the lobed phenotype may be ancestral for this taxon, and it persisted with colonization of the transition zone.

Lobed leaves may also be a defense adaptation. Feeding experiments including a plant species with lobed young leaves that develop into unlobed adult leaves (leaf heteroblasty) showed that tortoises prefer unlobed leaves^58^. On the Galápagos, adult leaves of lobed *Scalesia* species are within the reach of several large browsers, including giant tortoises and land iguanas (Supplementary Data 8)^14,59–61^. Intriguingly, *S. divisa*, the tallest lobed species^14^, has the least extreme lobed phenotype. It is thus possible that it needs less protection from herbivores.

While leaf morphology appears to have contributed to an adaptive radiation in *Scalesia*, lobed and unlobed species also differ in their floral structures. Most *Scalesia* species produce only disc florets from the centers of their composite flower heads. However, all but one lobed species (*S. divisa*) also occasionally produce ray florets along the circumference of the flower head (partially heterogamous)^13^. The environment in which lobed species are found thus may select for both lobed leaves and a heightened ability to attract pollinators^62^. Another possibility is that both traits are linked and selection only acts on one of them. However, *S. affinis*, the only *Scalesia* species that always produces disc and ray florets in the same capitulum, also produces entire leaves^13^, indicating that these two traits are not fully linked within the genus. Future studies should investigate selective pressures influencing *Scalesia*’s floral evolution. Based on this evidence, we tentatively suggest that differences in selective pressure regimes—whether driven by climate adaptation, defense against herbivores, selection linked to flower morphology, or a combination of these factors—could in part explain why different sets of genes are utilized to facilitate this lobed phenotype.

### Concluding remarks and implications for conservation

Our results suggest that a set of particular leaf-development genes have likely been under selection in *Scalesia*. They further indicate that the plants’ enormous phenotypic diversity in leaf lobing is at least partly adaptive, reflecting past work that has shown positive selection to be more common in rapidly radiating plant lineages^36,37^. The lobed leaf phenotype in *Scalesia* evolved independently within three different subclades via distinct genetic routes, rather than hybridization as would be predicted from the ‘hybrid swarm’ theory of adaptive radiation^63^ and in contrast to the extensive interspecies gene flow reported in Galápagos finches^10^. However, hybridization may have played a conceptually different role in the *Scalesia* adaptive radiation, as the ancient and divergent alleles potentially exploited by natural selection have been preserved via the allopolyploidization event early in *Scalesia*’s history^41^. The Hawaiian silversword radiation was similarly initiated by an early allopolyploidization event^64^. Only future genomic studies of other taxa can determine if this is a universal feature of plant adaptive radiations on oceanic archipelagos^9^. These insights into the specific components of the developmental pathway under selection early in the evolutionary history of a classic plant adaptive radiation illustrate how a novel phenotype can drive rapid ecological diversification.

Of critical relevance for the future existence of these iconic plants, our study has revealed limited inter-island gene flow between *Scalesia* populations that are currently considered conspecific. In many cases, this long-term isolation, along with intra-specific differentiation in leaf morphology, suggests incipient speciation that should be considered in nature management planning. We therefore suggest that every isolated population should be managed as a separate conservation unit, and the taxonomic status of several *Scalesia* species should be re-evaluated, which may have implications for their IUCN status. For example, *Scalesia affinis* is the only species currently IUCN categorized as lower risk, but if the divergent populations are split into two different species, their conservation status should be updated. This will have direct implications for ongoing Galápagos biodiversity conservation efforts.

## Methods

### Sampling of leaf materials for genomic sequencing

Sampling of fresh leaf material from 40 naturally occurring *Scalesia* populations from 14 of the 15 accepted species, two subspecies, as well as three potential hybrid species (Supplementary Data 2) was performed under permit number PC-001/98 PNG from the Ecuadorian Institute of Forestry, Natural Areas, and Wildlife. From each population, two young leaves were collected from 30 randomly selected individuals during the years 1998-2004 and stored in silica gel until further processing. If fewer than 30 individuals were available, the entire population was sampled. In 2020, a subset of 5–10 samples per population were randomly selected for DNA isolation (Supplementary Data 1 and 2). To supplement these collections and realize population samples from all *Scalesia* species, small leaf samples from pressed and dried herbarium specimens in the Charles Darwin Research Station Herbarium (CDS) were collected using sterile forceps. The subsequent genetic research was performed under research permit MAAE-DBI-CM-2021-0213 from the Ecuadorian Ministry of the Environment, Water, and Ecological Transition. For each sampled population as well as for the herbarium specimens, the habitat and climate (dry, transition zone, humid) was assessed in a previous study^65^ (Supplementary Data 1).

### DNA extraction, library building, and genomic resequencing

Genomic DNA was extracted with a DNeasy 96 Plant Kit (Qiagen) or the DNeasy Plant Mini kit (Qiagen) according to the manufacturer’s protocol with the following minor modifications. For the DNeasy 96 Plant extraction, dried leaf tissues were ground in a Qiagen collection microtube with a 3-mm tungsten carbide bead and mixed with 400 μl of lysis solution (Buffer AP1), 1 μl RNase A (100 mg/ml) and 1 µl reagent DX. The mixture was incubated at 65 °C for 10 min and the microtubes were inverted two times to homogenize the content. To precipitate detergent, proteins and polysaccharides, 130 µl of buffer P3 was added and microtubes were incubated for 10 min in the freezer at −20 °C. The plates containing microtubes were centrifuged at 3213×*g* (5500 rpm) with Sigma 4K15, and the supernatant was transferred into a new collection microtube and mixed with 600 μl of buffer AW1. All mixture was transferred to the DNeasy 96 plate assembled on top of the S-block to precipitate the DNA to the membrane, and the sandwiched plates were centrifuged for at least 15 min at 3213×*g* (5500 rpm). The membranes were washed with 800 µl of buffer AW2. To remove the greenish color from the membranes, an additional washing with 800 µl of buffer AW2 was applied. The DNA from the membranes was eluted with 50 µl of buffer AE to obtain a high-concentration DNA yield. A second elution with 70 µl buffer AE was also performed to collect DNA for additional analyses. For the DNeasy Plant Mini extraction, leaf tissue was disrupted using a TissueLyser and two stainless steel beads. For the herbarium samples, an overnight incubation step with 20 µL proteinase K (20 mg/mL) was added after the initial cell lysis step. The DNA extracts were sent to the commercial provider Novogene UK for shearing to a target length of 350 bp, followed by double-stranded DNA genomic library preparation, indexing and amplification via PCR, and 150-bp PE sequencing on the Illumina NovaSeq 6000 platform. An average of 35.5 Gbp (min: 3.1 Gbp, max: 219 Gbp, median: 30.9 Gbp) of raw sequencing data were produced per sample.

### Mapping to the reference genome

*Scalesia* is an allotetraploid genus with the two subgenomes being fully resolved in the reference genome used for mapping^41^. Thus, it was effectively treated as diploid in downstream analysis, as is common for polyploids with fully resolved subgenomes^66^. For raw read processing and mapping, the paleomix v1.2.14^67^ pipeline was used. Within paleomix, residual adapters were removed using AdapterRemoval v2.3.1^68^ and paired reads with at least 11 bases of overlap were collapsed into one read. BWA v0.7.17 mem^69^ was used to map the reads against the *Scalesia atractyloides* reference genome^41^. PCR duplicates were removed using PicardTools v2.25.5 MarkDuplicates (http://broadinstitute.github.io/picard). Mapping statistics were obtained from the paleomix summary files.

### Functional annotation of genes

For the functional annotation, the annotated *S. atractyloides* reference genome^41^ was used. Coding regions from 43,093 gene models were compared with *Arabidopsis thaliana* annotations (TAIR10 representative gene model proteins)^70^ using the blastx command in BLAST+ v2.9^71^ using an E-value threshold of 1 × 10^−6^. About 39,279 (91.1%) genes matched a TAIR10 annotation. The “org.At.tair.db”^72^ package was then used in R v4.3.1 to get the gene symbols associated with the TAIR locus IDs.

### Genotype likelihood estimates

Most samples in this study were sequenced to low sequencing depth (mean: 4.8X, SD: 3.24, Supplementary Data 1). Therefore, analyses including all samples are based on genotype likelihood methods suitable for low-genome coverage datasets. For some analyses, higher sequencing depths were required. We thus sequenced one sample from most populations to higher depth (8X to 17.6X, Supplementary Data 1).

CallableLoci from GATK v3.7-0^73^ was used to estimate which parts of the nuclear reference genome were reliably mappable based on mapping quality and sequencing depth distribution as described below. For each sample with sequencing depth >8X (see Supplementary Data 1), callableLoci was run with a minimum base quality of 20, a minimum mapping quality of 30, a minimum depth of one-third of the average depth, and a maximum depth of three times the average depth. Regions with excessive sequencing depth, no coverage, and with low mapping quality were extracted from the resulting bed files, the bed file for all samples with sequencing depth >8X were combined, and overlapping regions were merged with bedtools v2.30^74^ *merge*. To generate the final bed file excluding regions with excessive coverage, no coverage, and low mapping quality in any of the samples with sequencing depth >8X, bedtools *complement* was used.

Genotype likelihoods were estimated for the nuclear genome of all samples with sequencing depth >0.4X (Supplementary Data 1) with angsd v0.935^47^ with the options -GL 2 -doGlf 2 -doPlink 2 -doMajorMinor 1 -SNP_pval 1e-6 -doGeno -1 -doPost 1 -minMapQ 30 -minQ 20 -minMaf 0.05 -doCounts 1 -doMaf 3 -geno_minDepth 2 -setMinDepthInd 2 -postCutoff 0.95 -remove_bads 1 -uniqueOnly 1. Only sites with sequence data for at least half of the individuals were considered. Variant sites that were in regions with low mapping quality, as well as those within regions of excessive sequencing depth identified with CallableLoci as described above, were removed, as these regions might originate from the mitochondrial or the plastid genomes, or are gene duplications and thus violate the assumption of a diploid site in the genotype likelihood estimation. For the admixture and PCA analyses, sites in linkage disequilibrium (LD) ≥0.3 were removed. LD was estimated using Plink v1.9^75^ with a window size of 50 and a step size of 3. The final dataset after filtering contained 5,632,487 variable sites. In addition, LD decay was estimated for all samples together and for each taxon separately using Plink v1.9^75^ with the --r2 gz, --ld-window-r2 0, --ld-window 100, and --ld-window-kb 10,000 options, using only individuals with a genotype rate of 0.5 (--mind 0.5) and a minimum per-site genotype rate of 0.1 (--geno 0.1). As the input files contained a large number of sites, the dataset was thinned, giving each site a 10% chance of being retrained (--thin 0.1). The distance between each SNP in base pairs was calculated using a custom awk script, and the mean value for each distance was used for plotting with ggplot2^76^ in R v4.3.1^77^, showing rapid LD decay (Figs. S30–54).

### Population structure estimates and selection scanning

PCangsd v0.98^78^ was used to generate a kinship matrix for the entire dataset. Samples that were first- or second-degree related were excluded from the analysis. For each pair of related samples, the sample with the highest mean sequencing depth was kept. PCAngsd v1.10^78^ was used to generate the covariance matrix on the reduced dataset. The analysis was run until convergence to a minor allele frequency tolerance of 0.0001. For the PCA, the R function prcomp was used with the covariance matrix. NGSadmix^79^ was run on the reduced dataset with up to 20 ancestral populations (*K*). Ten independent runs with different seeds were performed for each *K* value. The run with the highest likelihood for each *K* value was used for plotting.

To estimate within- and between-species population statistics at neutral sites, fourfold degenerate sites (i.e., sites that result in no amino acid change; silent sites) were used. To generate a list of fourfold degenerate sites from the *S. atractyloides* reference genome and genome annotation^41^, Degeneracy (https://github.com/tvkent/Degeneracy) was used with bedtools v2.26.0^74^. The resulting bed file was then converted into an angsd sites file following the angsd documentation and indexed with angsd v.0.941^47^. To estimate Tajima’s *D* and theta, first the site allele frequency likelihood (SAF) was estimated for each species/population at fourfold degenerate sites with a minimum base quality of 20 (-minQ 20), a minimum mapping quality of 30 (-minMapQ 30), removing reads with multiple best hits (-uniqueOnly 1), removing primary, failure and duplicate reads (-remove_bads 1), using only sites that were covered in at least half of the individual (-minInd n/2), performing BAQ computation (-baq 1), adjusting the mapping quality for excessive mismatches (-C 50), using the reference genome as the ancestral state and only using fourfold degenerate sites (-sites) using angsd v0.940^47^. The resulting SAF was then used to generate a folded site frequency spectrum (SFS) with *realSFS*, followed by *realSFS saf2theta* and *thetaStat print* from angsd v0.940^47^ to generate per-site theta estimates. The per-site estimates were then used to calculate genome-wide Tajima’s *D* and nucleotide diversity in R^77^. To estimate 95% confidence intervals (CI), 1000 bootstraps of the per-site estimate files were performed and the resulting bootstrap files used to calculate Tajima’s *D* and nucleotide diversity. To estimate *d*_*xy*_ between species pairs, first angsd v0.940 was run as for the genotype likelihood estimation (see above) with -skipTriallelic and only using fourfold degenerate sites (-sites). The recovered sites were then used to generate allele frequency files (mafs) per species using angsd v0.940^47^ with the same options as before, but removing the -SNP_pval flag. This ensures the inclusion of sites that are fixed in one population. The resulting mafs files were then used to calculate global per-site *d*_*xy*_ between species pairs using a custom R script based on one by Joshua Penalba (https://github.com/mfumagalli/ngsPopGen/blob/master/scripts/calcDxy.R).

*F*_*ST*_ was estimated using angsd v.0.937. For each species, first the SAF was estimated using the *-dosaf 1* option, a minimum mapping quality of 30 and a minimum base quality of 20, and only using sites that are covered in at least half of the individuals. The 2D SFS was then calculated for each pairwise comparison, followed by the actual *F*_*ST*_ estimation using realSFS from angsd v0.937 with a folded spectrum. In addition to calculating the global estimate, *F*_*ST*_ was also estimated in non-overlapping 10-kbp sliding windows for species comparisons containing one species with lobed leaves and the unlobed species within clade *c,* which has the lowest *F*_*ST*_ to the lobed species (*S. crockeri* in every case) (Supplementary Data 9) in order to identify genes that might be responsible for the lobed leaf phenotype. Each group contains between 10 and 29 individuals (Supplementary Data 9). For the sliding window analysis, only windows with at least 100 SNPs were considered. In addition to *F*_*ST*_, Fay and Wu’s *H*^46^ was calculated in the same 10-kbp windows (see above) for each species using angsd v.0.937. Only sites that are covered in at least half of the samples were considered. realSFS saf2theta followed by thetaStat do_stat was then used to calculate genome-wide statistics for several neutrality tests, including Fay and Wu’s *H* in non-overlapping 10-kbp sliding windows. To polarize the SFS, the ancestral state was estimated using one sample with sequencing depth >8X from two early branching *Scalesia* species (*S. stewartii*: HALM35_20, *S. villosa*: HAMP28_35). First, bam files were downsampled to the same sequencing depth (13X after MAPQ30 filtering). Then angsd v.0.940^47^ was used with the -doFasta 2 option, a minimum mapping quality of 30, a minimum base quality of 20, -remove_bads 1, -uniqueOnly 1, -explode 1, and -C 50 to generate an ancestral state fasta file. *F*_*ST*_ values were *Z*-transformed, and windows with a *Z-*value above 4 and a negative Fay and Wu’s *H*, indicating positive selection, for the lobed species were considered as outlier windows putatively under selection. To investigate if any of these outlier regions are likely related to differences in leaf morphology, we examined genes within these regions that had been previously identified to be involved in the regulation of leaf growth and development in *A. thaliana*. For this we used multiple sources^55–57^ to compile a list of 716 candidate *A. thaliana* genes (LCgenes_2.0, Supplementary Data 10), corresponding to 1636 *Scalesia* genes (Supplementary Data 11).

A GO enrichment analysis was performed on the combined list of putatively selected LCgenes_2.0 genes, using GO terms from *A. thaliana* TAIR10 (89) BLAST results (see functional annotation). The *R* package topGO^80^ was used with Fisher’s exact test and the weight01 algorithm, using the LCgenes_2.0 list (Supplementary Data 10) as a null list. A significance threshold of *p* < 0.05 was used, and only GO terms describing biological processes were considered.

For closely related lobed and unlobed species of clade c, we ran a GWAS analysis based on genotype likelihoods. For this, first angsd v.0.940^47^ was used to generate genotype likelihoods with the options -GL 2, -doGlf 2, -doPlink 2, -doMajorMinor 1, -SNP_pval 1e-6, -doGeno -1, -doPost 1, -minMapQ 30, -minQ 20, -minMaf 0.05, -doCounts 1, -doMaf 3, -geno_minDepth 2, -setMinDepthInd 2, -postCutoff 0.95, -remove_bads 1, -uniqueOnly 1, -C 50. To estimate associations with leaf phenotype, angsd v0.935^47^ was used on the previously generated genotype likelihoods file using the -doAsso 2, -doMaf 4, -minHigh 30, -minCount 30 options. To account for population structure, a PCA was generated by first removing SNPs in LD and then running PCangsd v1.10^78^ as described above, and the first 20 PCs were used as covariates. Sites that were in regions with low mapping quality, as well as those within regions of excessive sequencing depth identified with CallableLoci as described above, were removed. Additionally, sites with a negative likelihood ratio test (LRT) value, those with missing data and those very few (0.42%) with a minor allele frequency above 0.5 were removed. LRT values were converted to *p* values using the pchisq() function in *R* v4.3.1^77^ with 1 degree of freedom and lower = F. Outlier SNPs were defined as those with a *p* value below or equal to the widely accepted value of 5e-08^81^. Overlap with gene regions was assessed using the GenomicRanges R package^82^.

### SNP discovery and genotyping

*Scalesia* is a tetraploid genus, while the closest relatives are diploid^23^. The reference used in this study is from a *Scalesia* species and contains both subgenomes. Therefore, the outgroups were mapped against each subgenome separately to avoid preferential mapping to one of the subgenomes. For the SNP calls, only samples with sequencing depth above 8X (see Supplementary Data 1) and outgroup samples were used (44 in total). For each sample, a g.vcf file was created with GATK v4.2.3^73,83^ HaplotypeCaller with a ploidy of 2, minimum base quality of 20, minimum mapping quality of 30 and stand-call-conf of 30. GATK GenomicsDBImport was used to create a genomics database using all 44 samples, followed by a joined SNP call using GATK GenotypeGVCFs with a stand-call-conf of 30. Afterward, only SNP positions were extracted and hard-filtered using the GATK best practice recommendations (QD >2.0, QUAL <30.0, SOR >3.0, FS >60.0, MQ <40.0, MQRankSum <−12.5, ReadPosRankSum <−8.0). Due to computational restraints, these steps were done on each chromosome separately, and the resulting vcf files were combined using Picard tools v.2.25.5 (http://broadinstitute.github.io/picard) GatherVcfs. Genotypes supported by fewer than six reads and with a genotype quality below 30 were set to missing using vcftools v0.1.17^84^.

### Phylogenetic analysis

For the phylogenetic analysis, only samples with sequencing depth above 8X and outgroup samples (see Supplementary Data 1) were used for gene tree and species tree estimations. A custom Python script was used to generate alignments for each *Scalesia* gene based on the called SNP variants (see above) using only genotypes with a minimum local coverage depth of 6 and a minimum genotype quality of 30. As no sample with high enough sequencing depth for *S. atractyloides* was available, the gene sequence for this species was extracted from the *S. atractyloides* reference genome^41^. For 2000 randomly selected genes, individual gene trees were estimated with IQ-Tree 2^85^ using models with optimal BIC scores. These gene trees were then used to generate a species phylogeny with ASTRAL^86^, which has been shown to be statistically consistent in analyses with variation among gene tree topologies due to incomplete lineage sorting^87^. To evaluate the robustness of the resulting phylogeny, the ASTRAL analysis was repeated three times with a different set of 2000 randomly selected genes and all replicates recovered the same resolution for all nodes with a posterior probability >0.9 in the replicate shown in Fig. 1A.

### Introgression analysis

For the introgression analysis, only samples with sequencing depth above 8X were used (see Supplementary Data 1). Quality-filtered, discrete genotypes were used as described under “SNP discovery and genotyping”. Dsuite DtriosParallel^88^ was run with the filtered vcf file using the ASTRAL tree that was obtained as described under “Phylogenetic analysis” to generate the D-statistics, followed by *F*_*branch*_ estimation using Dsuite Fbranch^88^. Results were plotted using the dtools.py script included in Dsuite.

### Pairwise sequentially Markovian coalescent (PSMC)

Changes in the effective population size over time were inferred for all *Scalesia* samples with sequencing depth above 12X (see Supplementary Data 1) using the pairwise sequentially Markovian coalescent (PSMC) model^89^. First, a diploid consensus sequence was generated for each chromosome using samtools v. 1.10 mpileup^90^ with a minimum mapping quality of 30 and a minimum base quality of 20, followed by bcftools v.1.10 call^91^. The resulting vcf file was converted to fastq with vcfutils.pl vcf2fq included in bcftools^90^ using a sequencing depth filter of minimum ⅓ the maximum depth and maximum 2x the maximum depth. The resulting files for each chromosome were combined into one input file for the *PSMC* analysis. *PSMC* was run with a “4 + 25*2 + 4 + 6” pattern (-p), and robustness was inferred by doing 100 bootstrap replicates. Demographic history was plotted using the mutation rate of sunflower (*Helianthus annuus*, 6.1 × 10^−9^ substitutions/site/year^92^) and a generation time of 3 years. This value was chosen because all shrubby species, as well as *S. microcephala* flowered within one year of germination under greenhouse conditions^14,18,19^. However, one species (*S. pedunculata*) did not flower within 3 years of germination in the greenhouse^14^. In the wild, it was found to be able to reach flowering within two years, but often takes 3 to 5 years to reach flowering^19^. This indicates that the generation times of *Scalesia* differ between species and even within species depending on the environmental conditions. Moreover, the total lifespan of a species can also influence generation time. Thus, the timing of effective population size in our PSMC analysis should be taken with caution.

### Leaf developmental transcriptomics

For the study of leaf developmental transcription in *Scalesia*, we used materials from the ex situ Galápagos plants living collection at the University of Copenhagen Botanical Garden greenhouses. From 14 living *Scalesia* individuals representing five different species (*S. pedunculata*, *S. atractyloides*, *S. divisa*, *S. retroflexa*, *S. gordilloi*) (Supplementary Data 7), healthy leaf tissues representing three developmental stages (young leaf early in development, young leaf late in development, mature leaf) were sampled into aluminum foil packets and immediately frozen in liquid N_2_. The samples were transferred to a −80 °C freezer for storage until they were shipped to the laboratory facility on frozen CO_2_. The tissues were crushed into a fine powder using the TissueLyser II, after which total RNA was extracted using a Spectrum Plant Total RNA Kit (Sigma, USA) with on-column DNA digestion following the manufacturer’s protocol. The extracted RNA samples were sent to the commercial NGS supplier Novogene UK, where a total amount of 1 µg RNA per sample was used as input for library preparation. A directional library was prepared using NEBNext® UltraTM Directional RNA Library Prep Kit for Illumina® (NEB, USA) following the manufacturer’s protocol. Indices were included to multiplex multiple samples. Briefly, mRNA was purified from total RNA using poly-T oligo-attached magnetic beads. After fragmentation, the first strand cDNA was synthesized using random hexamer primers followed by the second strand cDNA synthesis. The strand-specific library was ready after end repair, A-tailing, adapter ligation, size selection, and USER enzyme digestion. After amplification and purification, the insert size of the library was validated on an Agilent 2100 Bioanalyzer and quantified using quantitative PCR. Libraries were then sequenced on the Illumina NovaSeq 6000 platform with PE150. Approximately 6 Gbp of raw sequencing data were generated for each library.

We converted the annotation of the *Scalesia atractyloides* genome from the general feature format (GFF3) to a coding sequence (CDS) file using a parsing script (gff2fasta_v3.pl) from Bitacora v1.3^93^. To identify adapter residual adapter sequences, we utilized AdapterRemoval v2.3.2 and then trimmed these adapter sequences using Trimmomatic v0.39. Then, we employed the pseudoalignment tool Kallisto v0.48.0^94^ to align the RNA to the CDS file generated from the *Scalesia atractyloides* genome assembly. This involved constructing an index with the CDS as the reference (algorithm: “kallisto index”) and performing pseudoalignment (“kallisto quant”) with 100 bootstraps. These raw alignment count metrics were utilized for the downstream transcriptomic analyses of leaf development. PCA was conducted using the pcaExplorer package v2.24.0^95^ within the R v4.2.2 environment to differentiate the transcriptional profiles across four developmental stages. Gene expression visualization was achieved with ggplot2 v3.4.2. After excluding the mature leaf data from further analysis, regulatory network inference was performed using GENIE3 (GEne Network Inference with Ensemble of trees) v1.20.0^96^. We constructed species-specific leaf developmental networks (referred to as L1) to identify interactions among genes involved in leaf development, and then focused on interactions between genes under selection in lobed species and their potential targets (referred to as L2). The L1 network was constructed using the top 2000 GENIE3 links, with all *Arabidopsis thaliana* transcription factor orthologs from PlantTFDB 5.0^97^ as regulators, and combined transcriptomic data from early, young, and late leaf stages as potential targets. The L2 network was constructed using the top 500 GENIE3 links, specifically targeting literature-curated genes under selection in lobed species. The resulting networks were visualized using Cytoscape v3.10.1^98^ to map the intricate relationships between key regulatory genes and their targets. Finally, we combined L1 and L2 networks using GENIE3’s merge function (merge > networks > intersection) and conducted GO enrichment analysis using Cytoscape’s stringApp 2.1^98^.

### Leaf morphometrics

Sampling of leaf material for morphometric analysis was performed under permit number PC-001/98 PNG from the Ecuadorian Institute of Forestry, Natural Areas, and Wildlife during the years 1999–2004. From 29 naturally occurring *Scalesia* populations covering 13 species, including both *S. baurii* subspecies, and three potential hybrid populations (Supplementary Data 2), five mature leaves per individual, judged to be of approximately the same age, were collected and pressed. In 2021, three leaves per individual and 10–12 individuals per population were randomly chosen for morphological analysis. Leaves that appeared not to be well pressed (with overlapping folds) were shortly submerged in hot water inside a glass beaker to rehydrate each leaf. Hereafter, it was flattened as much as possible and pressed again. Populations used for leaf morphological analyses corresponded to the ones used for population genomic analysis, but in some cases, no pressed leaves were available. When possible, pressed leaves collected at a later sampling year from the same site or close by, were included; otherwise the populations were left out of the morphological analysis. Fourteen characters were determined on digital images of scanned leaves with the software WinFOLIA Pro v2014 (Regnet Instruments Inc., Quebec, Canada), specifically developed for the characterization of leaf morphology. The option “Leaf Morphology Analysis” was applied with default settings, except in cases where the leaf appeared with holes. In such cases, the holes option was chosen to avoid holes being included in the leaf perimeter estimation of the leaf blade. The WinFOLIAleaf morphology analysis requires defining the junction between the petiole and lamina (leaf blade). We defined the lamina as the wide part of a leaf, which accounts for photosynthesis. The 16 measured characters were: leaf area, perimeter (without petiole), vertical length, horizontal width, average horizontal width, aspect ratio, form coefficient, blade length, maximal perpendicular width, position of the maximal perpendicular width, perpendicular width1, perpendicular width2, LobeAngle1, LobeAngle2, petiole length, and petiole area. LobeAngle1 and LobeAngle2 were omitted before analysis as these measurements were not comparable across the morphologically very variable species. See supplemental material for further descriptions of the characters (Supplementary Data 12). Only measurements from entire leaves (petiole not broken) were used in further analyses. In addition to the 16 measurements, two ratios were estimated: perimeter/(blade length) and perimeter/(leaf area) (Supplementary Data 4).

The morphological data were analyzed in R v4.3.1^77^ using the MorphoTools2^99^ package. Correlation between measurements was tested with the cormat function using the ‘Spearman’ method. One measurement (horizontal width) was removed due to a high correlation (*r* > |0.95|) with maximal perpendicular width. A PCA was performed using the pca.calc function. Per-species and per-population centroids were obtained from the pca.calc output. Per-species centroids were used to estimate the distance of each individual datapoint to its species mean with the following formula:

1 $$\sqrt{{({\,{PC}1}_{i}-\,{{PC}1}_{{centroid}})}^{2}+{({\,{PC}2}_{i}-\,{{PC}2}_{{centroid}})}^{2}}$$

### Reporting summary

Further information on research design is available in the Nature Portfolio Reporting Summary linked to this article.

## Supplementary information

Supplementary Information

Description of Additional Supplementary Files

Supplementary Data 1

Supplementary Data 2

Supplementary Data 3

Supplementary Data 4

Supplementary Data 5

Supplementary Data 6

Supplementary Data 7

Supplementary Data 8

Supplementary Data 9

Supplementary Data 10

Supplementary Data 11

Supplementary Data 12

Reporting Summary

Transparent Peer Review file

## Data Availability

DNA sequencing data generated for this study have been deposited in the European Nucleotide Archive under the study accession code PRJEB70770. The RNA sequencing data generated for this study have been deposited in the European Nucleotide Archive under the study accession code PRJEB74314. A complete list of accession codes for each sample can be found in Supplementary Data 1 (DNA) and Supplementary Data 7 (RNA). The previously published reference genome of *Scalesia atractyloides*^41^ used in this study can be found on Dryad (10.5061/dryad.8gtht76rh). Source data for figures are available on Dryad (10.5061/dryad.j9kd51cr0).

## Code availability

The programs, R packages, and functions used are described in detail and cited in the Methods section. The scripts and commands that call these tools, some example input and output files, and the Cytoscape analysis file are available on Dryad (10.5061/dryad.j9kd51cr0) and the associated Zenodo repository (10.5281/zenodo.18865669).
