# Supplementary material for: The genomic basis of adaptive leaf variation in the Galápagos giant daisies
Source: Nat Commun. 2026 Apr 16;17:5319. doi: 10.1038/s41467-026-71865-3 (PMC13273160; doi:10.1038/s41467-026-71865-3)
Supplement: Supplementary file 1 — Supplementary Information [file 41467_2026_71865_MOESM1_ESM.pdf]

Supplementary material for: The genomic basis of adaptive leaf variation in the Galápagos giant daisies

Authors

Vanessa C. Bieker[1,2]*, Siyu Li[3], José Cerca[1,4,5,6,], Paul Battlay[7], Mohsen Falahati Anbaran[1], Amit Sharma[8], Patricia Jaramillo Díaz[9,10,11], Mario Fernández-Mazuecos[12,13,14], Jazmín Ramos-Madrigal[15], Sarah L. F. Martin[1], Luisa Santos-Bay[15], Gitte Petersen[16], Ole Seberg[17], Pablo Vargas[14], Rasmus Nielsen[18], M. Thomas P. Gilbert[1,14], Gonzalo Rivas-Torres[19,20], James Leebens-Mack[21], Loren H. Rieseberg[22], Lene R. Nielsen[23], Neelima Sinha[3], Michael D. Martin[1]*

SI Notes

Supplementary Note 1 Within-island gene flow

High levels of gene flow on Santiago Island

Within clade *b*, no separation between *S. atractyloides* and *S. stewartii* can be made based on the phylogeny and the admixture analysis (Fig. 1A, Fig. S4). In addition, they show low pairwise F_{ST} values (Fig. S2) and cluster together in the PCA (Fig. 2A). This is in contrast to previous findings based on GBS data where *S. atractyloides* and *S. stewartii* formed strongly supported monophyletic groups[1]. However, only *S. stewartii* samples from Bartolomé were included in their analysis. We found that the *S. stewartii* population from Bartolomé clustered a little apart from the *S. stewartii* and *S. atractyloides* samples from Santiago on the PCA (Fig. 2A). PSMC suggests that the *S. stewartii* populations from Santiago and Bartolomé split around 30,000 years ago (Fig. S3). Although F_{ST} between *S. stewartii* from Bartolomé and Santiago is relatively low (0.063696, Table 1), it is higher than that between *S. stewartii* and *S. atractyloides* var. *darwinii* and similar to *S. stewartii* vs *S. atractyloides* var. *atractyloides* (Fig. S2). Moreover, an admixture analysis of including only *S. stewartii* and *S. atractyloides* samples shows that for *K*=2, samples cluster based on island rather than species (Fig. 55). Due to the limited gene-flow between islands observed for other species within the genus, the plants' limited ability for long-distance dispersal[2], and their separation on the PCA, we conclude that these populations are well on the way to speciation. However, within the island of Santiago low genetic diversification between *S. atractyloides* and *S. stewartii* was found, suggesting that these two taxa are indeed one species.

Ancient interspecific hybridization on San Cristóbal Island

Clade *c-4* contains three taxa, *S. divisa*, *S. incisa*, and the potential hybrid *S. divisa* x *incisa*, where only *S. incisa* forms a monophyletic group in the phylogeny (Fig. 1A). In addition, they cluster together on the PCA when considering the first two PCs (Fig. 2A), have very low pairwise F_{ST} (Fig. S2), and almost identical PSMC curves (Fig. S3). In the admixture analysis, they are assigned to one main cluster up until *K*=19 with *S. divisa* and *S. divisa* x *incisa* showing low levels of ancestry from several other clades (Fig. S4) that may stem from incomplete lineage sorting during the rapid diversification of clade *c*. For *K*=20, two main genetic clusters are found, with *S. incisa* being fully assigned to one and two of the three *S. divisa* populations being fully or mainly assigned to the other. The third *S. divisa* population and *S. divisa* x *incisa* are partly assigned to both genetic clusters, with low levels of ancestry from other clades showing a gradient rather than a clear separation between the taxa. Our results agree with a previous study based on leaf morphology, AFLP and plastid microsatellites that found a separation between *S. incisa* and *S. divisa* while proposed hybrids were clustered in a continuum between the two extrema[3]. The two species and the proposed hybrids only occur on the island San Cristóbal in the Galápagos[2]. There is geological evidence that this island is composed of two separate volcanoes, one in the north-east and one in the south-west. It is likely that these volcanoes were once separated by a sound and later merged into one single island[4]. The timing of the merge is unknown. However, the youngest lava flows are likely less than 1,000 years old[4]. Only *S. divisa* is currently found on the south-west part of the island, right at the border to the younger lava flow that connects the south-west and the north east parts[3,4]. Based on the current distribution of the species, Nielsen et al.[3] suggested

that *S. divisa* and *S. incisa* diversified on these two separated volcanoes and once the volcanoes merged, came into secondary contact and were able to form hybrids. When species come into secondary contact following allopatric speciation, reproductive isolation may be incomplete, leading to occasional or extensive hybridisation. If hybridisation is extensive and the hybrids are fit, it may lead to the merging of the two species into one hybrid species. If the hybrids are less fit, it could lead to selection against hybridisation and the completion of reproductive isolation (reinforcement)[5]. Incomplete reproductive isolation and thus hybridisation may be common in recently-diversified clades and is therefore especially of interest in adaptive radiations[6]. It is however also possible that *S. divisa* x *incisa* is a remnant ancestral population from which *S. incisa* and *S. divisa* diversified rather than a recent hybrid population, which would indicate an ongoing speciation event. Future studies should use simulation approaches to infer if these taxa evolved separately and came into secondary contact with incomplete reproductive isolation.

Supplementary Note 2 Taxonomic revision is needed for genus *Scalesia*

We generated an ASTRAL phylogeny using only the high-depth (> 8 X) samples based on called genotypes (Fig. 1A). The phylogeny shows high node support values for the major clades and is largely congruent with a previously published phylogeny based on ca. 10,000 genomic loci[1]. The ASTRAL phylogeny revealed three major clades (*a-c*) and clade *c* can further be subdivided into five subclades (clade *c-1* - *c-5*). Our phylogeny and the previously published one differ in the placement of *S. affinis*: in the phylogeny by[1], it forms a clade with *S. microcephala* and *S. cordata*, whereas our phylogeny, it forms a monophyletic group within clade *c-1.* This placement was also found in previous coalescent-based analysis, albeit with low support[1]. This discrepancy in the phylogeny may be due to the fact that no *S. affinis* samples from Isabela are included in our phylogeny as this population clusters closer to *S. microcephala* and *S. cordata* in the PCA (Fig. 2A) and shares some ancestry with these species in the admixture analysis (Fig. S4).

The main difference between ours and the previously published phylogeny[1] is the placement of *S. gordilloi*. In the previously published phylogeny[1], *S. gordilloi* forms a clade with *S. incisa* and *S. divisa*, whereas in our phylogeny, *S. gordilloi* groups with *S. pedunculata*, making *S. pedunculata* non-monophyletic. However, this relationship was found in some of the coalescent-based analysis by[1]. *Scalesia gordilloi* is the most recently described species of the genus[7] and is endemic to the island of San Cristobal. On this island, *S. incisa*, *S. divisa*, and *S. pedunculata* also occur. However, their present distributions do not overlap[7,8]. The discordance in the placement of *S. gordilloi* may be due to an ancient hybrid origin of this species and the different provenance of the genomic markers used to estimate the phylogenies, e.g. the genotyping by sequencing (GBS) study[1] lacked a reference genome that would enable separate analysis of homeologous chromosomes during SNP discovery. Indeed, we found high levels of allele sharing of *S. gordilloi* with clade *g* and clade *d* based on F_{branch} statistic (Fig. S5) and admixture analysis (Fig. S4).

Supplementary Note 3 Two widespread *Scalesia* species form species complexes

One of the most widespread *Scalesia* species is *S. pedunculata*, which is found on Santiago, Santa Cruz, San Cristobal, and Floreana. We here included populations from all four islands. Due to the placement of *S. gordilloi* in the phylogeny (Fig. 1A), this species does not form a monophyletic group but is rather split into two clades that include populations from San Cristobal and Floreana (*S. pedunculata* 1) and populations from Santiago and Santa Cruz (*S. pedunculata* 2), except for one Santa Cruz individual that groups together with the Floreana population. However, F_{branch} (Fig. S5) indicates high levels of shared alleles between the two groups, potentially due to incomplete lineage sorting during the rapid diversification of clade *c*. The two *S. pedunculata* groups also segregate in the PCA (Fig. 2A) and show strong population structure in the admixture analysis by forming two different genetic clusters already for *K*=12, with all *S. pedunculata* 2 samples from Santa Cruz showing low levels of ancestry from *S. pedunculata* 1 up until *K*=19 (Fig. S4). For *K*=19-20, *S. pedunculata* 2 is further split into two genetic clusters separated by island. Moreover, populations from different islands show moderate ($0.05 < F_{ST} < 0.15$) to high ($0.15 < F_{ST} < 0.25$) genetic differentiation (Table 1). PSMC analysis indicates that the Santa Cruz and Santiago populations split around 100,000 years ago, whereas the Floreana and San Cristobal populations split around 60,000 years ago (Fig. S3). Together, this evidence indicates limited gene-flow between islands and calls for a re-evaluation of the species *S. pedunculata* that integrates genomic data with morphological characters. This also has implications for conservation efforts. The species is currently listed as "vulnerable" by the IUCN[9]. However, the status may change if the two *S. pedunculata* clades are assessed independently.

The PCA (Fig. 2A) and admixture (Fig. S4) analysis indicates strong structure within *S. affinis* populations from Isabela and those from Floreana and Santa Cruz. In addition, there is high F_{ST} between the *S. affinis* Isablea population and the Santa Cruz (F_{ST} = 0.24) and Floreana (F_{ST} = 0.27) populations, whereas the Floreana and Santa Cruz populations show moderate differentiation (F_{ST} = 0.08) (Table 1). Unfortunately, the Isabela population was not included in the phylogeny due to too low sequencing depth. The structure within *S. affinis* is in accordance with previous findings based on amplified fragment length polymorphism (AFLP) and chloroplast markers in *S. affinis*[10] where two unities were found, one consisting of samples from Isabela and the other from samples from Santa Cruz and Floreana. Interestingly, *S. affinis* was first described as two different species (*S. affinis* Hooker and *S. gummifera* Hooker) and later merged into one species[2]. The material used to describe *S. gummifera* was from Isabela and the one for *S. affinis* from Floreana. Our genetic results support the original splitting into two different species: *S. affinis* for the Santa Cruz and Floreana populations and *S. gummifera* for Isabela populations. Despite the Santa Cruz and Floreana *S. affinis* populations being assigned to the same genetic cluster in the admixture analysis for up to *K*=20, the two populations likely diversified around 100,000 years ago based on PSMC (Fig. S3). The landmasses of today's Floreana and Santa Cruz were connected around 1 Ma and had separated by the time of the last glacial maximum 21,000 years ago. However, the exact date when the landmasses became separated is unknown[11]. It is thus possible that the split time between the two *S. affinis* populations indicates the time the landmasses became separated rather than a colonization event. Together with data from other species occurring on several islands, future studies may be able to more precisely date the emergence and separation of major landmasses on the Galápagos.

Supplementary Note 4 Transcriptomics of leaf development

Five *Scalesia* species, two with lobed leaves (*S. retroflexa*, *S. divisa*) and three with unlobed leaves (*S. atractyloides, S. pedunculata, S. gordilloi*), were subjected to comprehensive transcriptomic analyses to elucidate the genetic basis of leaf lobing during development. Leaves from four developmental stages per species—early development, late development, young leaf, and old leaf—were collected for this analysis (Supplementary Data S8). The RNA mapping to the reference was conducted using kallisto[12]. The average number of reads processed per sample was 22,309,602 (standard deviation: 1,678,664) and the average number of reads pseudoaligned was 13,649,155 (61% of reads were pseudomapped; standard deviation: 1,140,916) (Supplementary Data S8).

Principal component analysis (PCA) was employed to contrast the transcriptomic profiles across these stages (Figure S56-S57). Early and late developmental stages clustered closely, suggesting a conserved phase of leaf morphogenesis across *Scalesia*, akin to patterns observed in other plant species[13]. Young leaf samples predominantly contributed to variance along the first principal component (PC1), with species-specific groupings emerging at this juncture, likely reflecting the onset of morphological differentiation. Consequently, it appears that the process of leaf lobing initiates between the late development and young leaf stages. Intriguingly, old leaf samples were distinctly separated from the earlier stages, implying substantial transcriptomic divergence (Figure S56). To concentrate on the genetic architecture of leaf lobing, the old leaf stage was excluded from subsequent analyses.

To understand the gene interactions regulating leaf development, we constructed species-specific leaf developmental networks using the GENIE3 algorithm, a machine learning-based algorithm that infers gene regulatory networks by taking gene expression data as input and outputting a ranked list of regulatory links between potential regulators and targets. In GENIE3, regulators are typically transcription factors or other upstream genes that influence the expression of other genes, while targets are the downstream genes whose expression is potentially regulated by these regulators through direct or indirect interactions. The algorithm aims to predict and rank these regulatory links based on patterns observed in gene expression data. We used all *Arabidopsis thaliana* transcription factor orthologs (PlantTFDB 5.0[14]) as regulators, and early, late, and young leaf transcriptomic data were combined and imported as potential targets. GENIE3 identified 82,459,889 potential regulatory links in *S. atractyloides*, 82,459,221 in *S. divisa*, 82,459,816 in *S. gordilloi*, 82,459,795 in *S. pedunculata*, and 82,459,679 in *S. retroflexa.* We constructed GENIE3 regulatory networks using the top 500, 1000, 2000, 3000, and 4000 predicted links. As the number of top links increased, the number of common genes across the five species also increased proportionally. However, the proportion of literature-curated genes among the common genes decreased from 50% (intersection of top 500 links) to 13% (intersection of top 2000, 3000 and 4000 links). There is no universally defined threshold for selecting links in GENIE3, as it is a tree-based method, and the appropriate threshold depends on data size and research objectives. Researchers typically select the top 0.5% to 1% of links when working with large-scale datasets[15,16]. To reduce false positives and noise in, and to focus on the most important leaf regulatory links, we selected the top 2000 links (~0.0024% of the total links) for network visualization. The resulting networks across the five species revealed 184 common genes (131 regulators and 53 targets) shared among all species, although no common edges were identified. This is not unexpected for a complex trait under polygenic selection[17]. The common genes include many that are known to regulate leaf development, such as *BEL1* (AT5G41410), *BEL2* (AT4G36930), and *AS1* (AT2G37630). Cytoscape

stringApp 2.1.1[18] was used for functional enrichment analysis. All five species-specific leaf developmental networks (network level L1) contain genes enriched for gene ontology (GO) terms such as regulation of gene expression (GO:0010468), gibberellic acid-mediated signaling pathway (GO:0009740), shoot system development (GO:0048731), and photosynthesis (GO:0015979). This suggests that the networks have multiple functions, involving hormones and shoot development that captures early leaf development.

To assess how selection impacts these networks, we identified literature-curated leaf developmental genes (LC genes) under selection based on F_{ST} outlier analysis, and examined their expression patterns (Fig. S22). However, none of these genes exhibited significantly different expression patterns between the lobed species with the gene under selection and all other species, at any time point, using Tukey HSD test (adjusted p < 0.001). Thus we hypothesize that these genes may influence leaf shape through interactions between genes (network structure) rather than through differences in expression levels. To test this hypothesis, we identified the regulatory interactions of these selected LC genes (L2) in each lobed-leaf species, retaining only those targets that were present in their corresponding leaf gene regulatory networks (L1) using Cytoscape merge function (merge>networks>intersection). We use the top 500 links (248,696 links in *S. divisa,* 106,584 in *S. retroflexa*) to avoid the false positive. Different LC gene sets were present in the intersected networks (L1+L2) of the two lobed species (Figure 4). In *S. retroflexa*, *LEUNIG* (*LUG*) and *CONSTITUTIVE PHOTOMORPHOGENIC 3* (*COP3*) are highly connected within the L1+L2 networks. In *S. divisa*, six LC genes under selection—ARABIDOPSIS MEDIATOR COMPONENT 14, A. THALIANA ERBB-3 BINDING PROTEIN 1, AUXIN RESPONSE FACTOR 4 (*ARF4*), GIBBERELLIN 20-OXIDASE 4 (*GA20OX4*), LOST MERISTEMS (LOM2), and A. THALIANA SUCROSE-PROTON SYMPORTER 2 (SUC2)—that are highly connected within the L1+L2 network. While mutants of these corresponding genes in *Arabidopsis* exhibit altered leaf morphology[19,20], only the *ARF4* and *GA20OX* mutants show differences in leaf lobing[21,22].

The omnigenic model[23–25] suggests that most complex traits (such as leaf morphology in this case) are influenced not only by a few core genes but also by a vast number of peripheral genes throughout the genome. The core genes have direct and strong effects on the trait. The peripheral genes, which often have effects on different biological processes or phenotypes (pleiotropic), also contribute to the overall phenotype through their interactions and regulatory influence, likely in subtle ways. Under the framework of the omnigenic model and network structure, we hypothesize that *Scalesia* leaf lobing was generated through alterations in gene regulatory networks (Fig. 4). The leaf developmental networks share a small number of core genes—hypothesized to be the 184 common genes in all four *Scalesia*—are directly responsible for leaf phenotype and are highly pleiotropic. These core genes are influenced by many upstream peripheral genes and are crucial for multiple developmental processes throughout leaf development. Thus, the leaf developmental network is decentralized, highly redundant, and stable enough to withstand negative selection[26]. Peripheral genes are more likely to be subject to directional selection[26]. In *S. divisa*, the L1 network showed a heterogeneity of 0.956 and a centrality of 0.026, while the combined L1+L2 network displayed a heterogeneity of 1.490 and a centrality of 0.127. In *S. retroflexa*, the L1 network had a heterogeneity of 1.111 and a centrality of 0.030, whereas the L1+L2 network had a heterogeneity of 3.583 and a centrality of 0.294. The increase in heterogeneity and centrality in the L1+L2 networks compared to the L1 networks indicates higher modularity in the leaf developmental networks and the presence of stronger hubs, enhancing the network's complexity and connectivity. This change could explain how *Scalesia* can generate such

morphological diversity within a short evolutionary time. Given that *ARF4*, gibberellic acid, and *LEUNIG* are all involved in the abaxial/adaxial leaf polarity pathway[27–30] and that these polarity identity genes are under selection in both lobed species, we conclude that the polarity regulation pathway is involved in the development of lobing in *Scalesia* leaves.

Supplementary Note 5 Ecology of *Scalesia*

These species may inhabit the three altitudinal zones of the islands: highlands, transition, and lowlands. The highlands are characterized by greater cloud cover and higher humidity (greater annual precipitation) compared to the sunnier and drier transition and lowland zones. The lowlands represent the altitudinal zone closest to the coast, featuring drier climates and more pronounced seasonal rainfall patterns compared to the higher zones. The lowlands also experience higher annual temperatures, leading to the presence of drought-adapted species such as cacti and various *Scalesia* species with tougher leaves than their high-altitude counterparts. Finally, the transition zone lies between the lowlands and highlands and features species from both zones. Typically, it experiences a more humid climate than the lowlands but with less precipitation than the highlands. Twelve of the 15 Scalesia species occur on only one or two islands. Of the 15 extant species, three (*S. cordata*, *S. pedunculata*, and *S. microcephala*) are recorded mainly in highlands, with some individuals and/or island populations observed in the transition zone. The remaining species (*S. baurii*, *S. villosa*, *S. stewartii*, *S. retroflexa*, *S. incisa*, *S. hellerii*, *S. gordilloi*, *S. divisa*, *S. crockerii*, *S. atractyloides*, *S. affinis,* and *S. aspera*) occur mostly in the lowlands, and in rare cases are observed up to the transition zone. Lobed leaves can reduce water loss through transpiration and have an increased heat dissipation rate[2,31]; thus they are thought to be an adaptation to dry, warm climates such as those at low elevations on the Galápagos archipelago[2].

Lobed leaves may also be a defense adaptation. Typical of other remote islands, the faunal composition of Galápagos differs substantially from the mainland, with more toothless herbivores such as birds, iguanas and tortoises, and less mammalian herbivores on islands[32,33]. Some island plant species display leaf heteroblasty, where juvenile and adult leaves may differ in shape, size and color[33]. Feeding experiments of Mascarene island plants that included one species (*Turraea casimiriana*) with lobed juvenile leafs and unlobed adult leaves showed that the Aldabra giant tortoise (*Geochelone gigantea*) prefers adult leaves[32]. As adult leaves of these plants are often outside the reach of tortoises and other flightless browsers[32], they may not need the same defenses, leading to leaf heteroblasty. Several large browsers are known from the Galápagos, including giant tortoises with 'saddleback' shells that allows them to reach vegetation up to 2 m high[33,34] and land iguanas that can stand on their hind legs to reach higher growing vegetation[35]; thus adult leaves of lobed leaf *Scalesia* are often within their reach (Supplementary Data S8)[2]. Intriguingly, *S. divisa* is the tallest lobed species, reaching up to 4 m[2] has the least extreme lobed phenotype. It is thus possible that it needs less protection from giant tortoises, land iguanas, and other flightless browsers. Diet analysis revealed that at least some *Chelonoidis* spp. feed on unlobed *S. pedunculata*[36], and land iguanas feed on unlobed *S. affinis*[35]; however it is unknown whether they also feed on other *Scalesia* species and if they prefer unlobed over lobed leaves. With this evidence, we tentatively suggest that differences in selective pressure regimes, with some species evolving a lobed phenotype due to climate adaptation and others as a defense adaptation to herbivores, could in part explain why different sets of genes are utilized to facilitate this phenotype.

While *Scalesia* leaf morphology appears to have contributed to the plants' adaptive radiation, lobed and unlobed species also differ in their floral structures. Most *Scalesia* species have homogamous capitula (with disc florets only), and only one species (*S. affinis*) has fully heterogamous capitula (with disc florets and ray florets)[1]. Despite these differences in floral structure, reproductive studies in several *Scalesia* species (*S. cordata, S. affinis, S. divisa*) showed that they are outcrossers with a self-incompatibility system that only allows for low rates of selfing[37–39]. All but one lobed species (*S. divisa*) have partially heterogamous capitula,

and this trait is only found in lobed *Scalesia* species[1]. *S. divisa*, the only lobed species with homogamous capitula, also has the weakest lobed leaf phenotype (Fig. 3, Fig. 1B). The environment in which lobed species are found thus may select for both lobed leaves and heterogamous capitula. Another possibility is that both traits are linked and selection only acts on one of them. Future studies should investigate *Scalesia*'s floral evolution.

SI figures

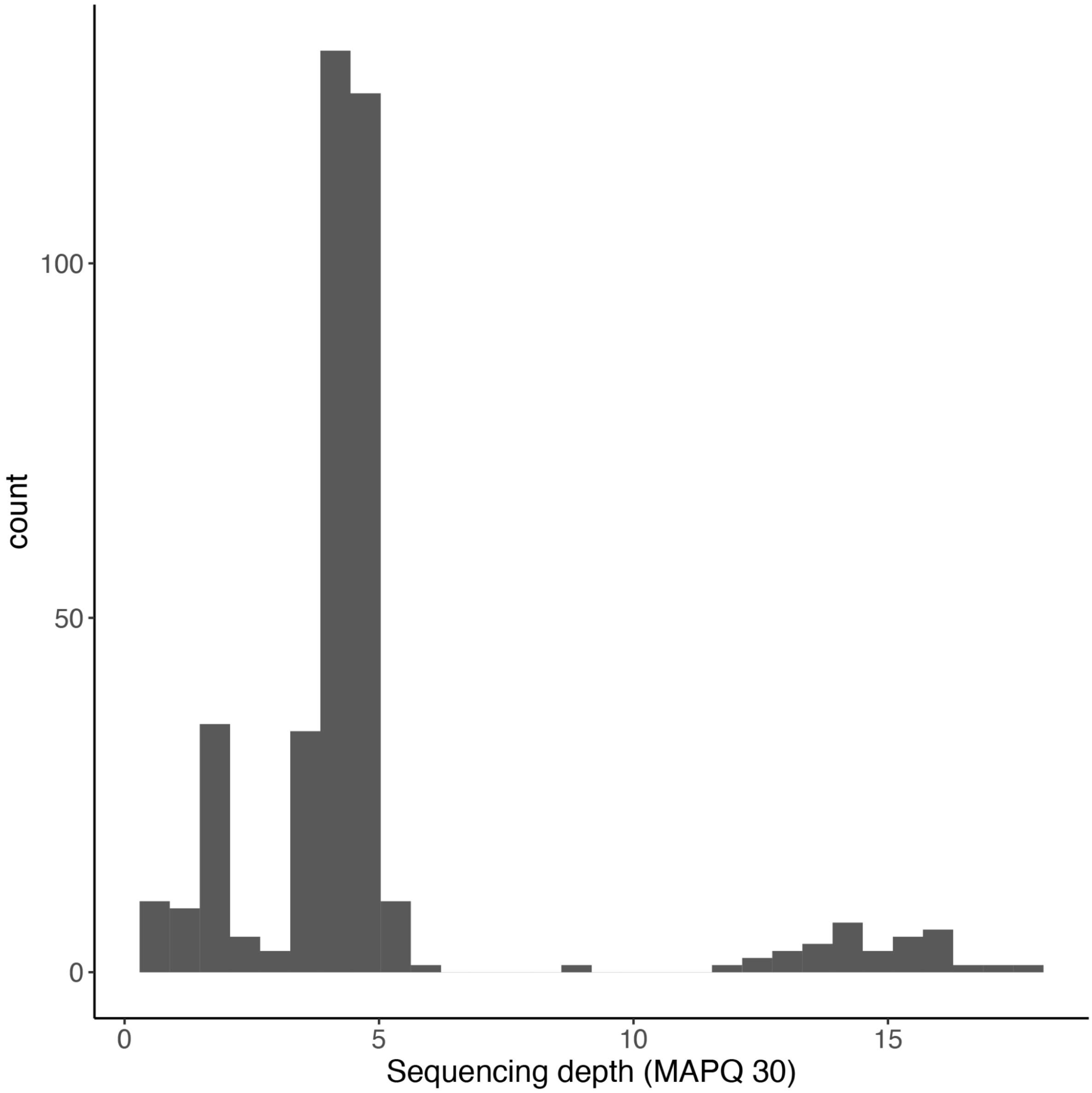

Fig. S1. Sequencing depth after MAPQ ≥ 30 filtering. Source data are provided on Dryad (https://doi.org/10.5061/dryad.8gtht76rh).

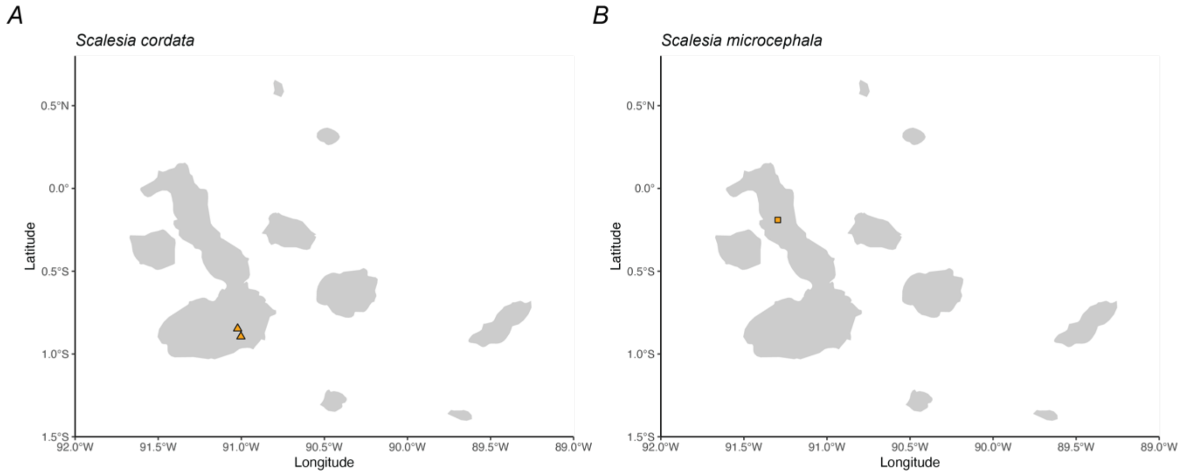

Fig. S2. Sampling locations for clade a species included in the genetic analysis. **A**: *Scalesia cordata*. **B**: *Scalesia microcephala*. Source data are provided on Dryad (https://doi.org/10.5061/dryad.8gtht76rh).

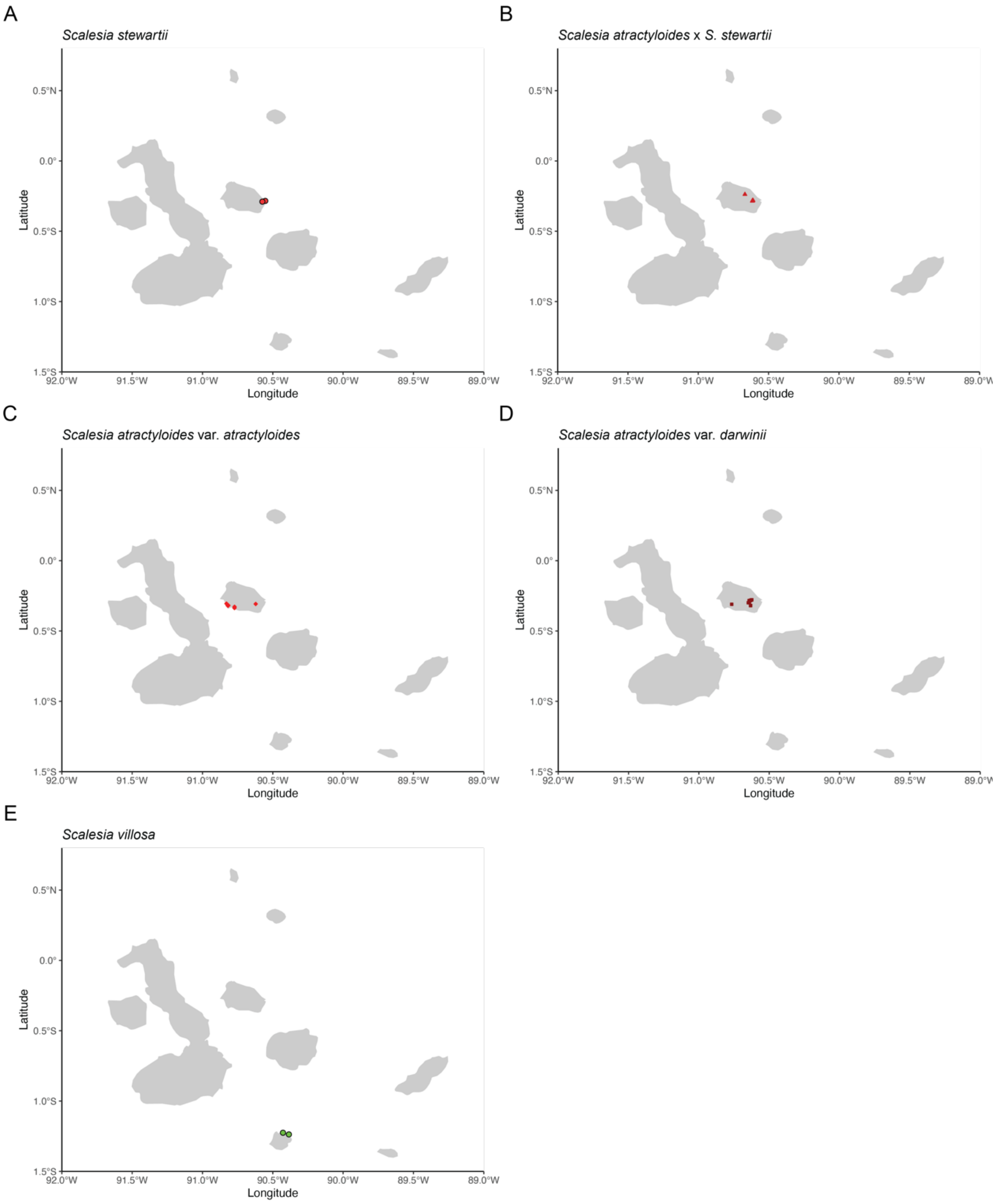

Fig. S3. Sampling locations for clade b species included in the genetic analysis. **A**: *Scalesia stewartii*. **B**: *Scalesia atractyloides* x *stewartii*. **C**: *Scalesia atractyloides* var. *atractyloides*. **D**: *Scalesia atractyloides* var. *darwinii*. **E**: *Scalesia villosa*. Source data are provided on Dryad (https://doi.org/10.5061/dryad.8gtht76rh).

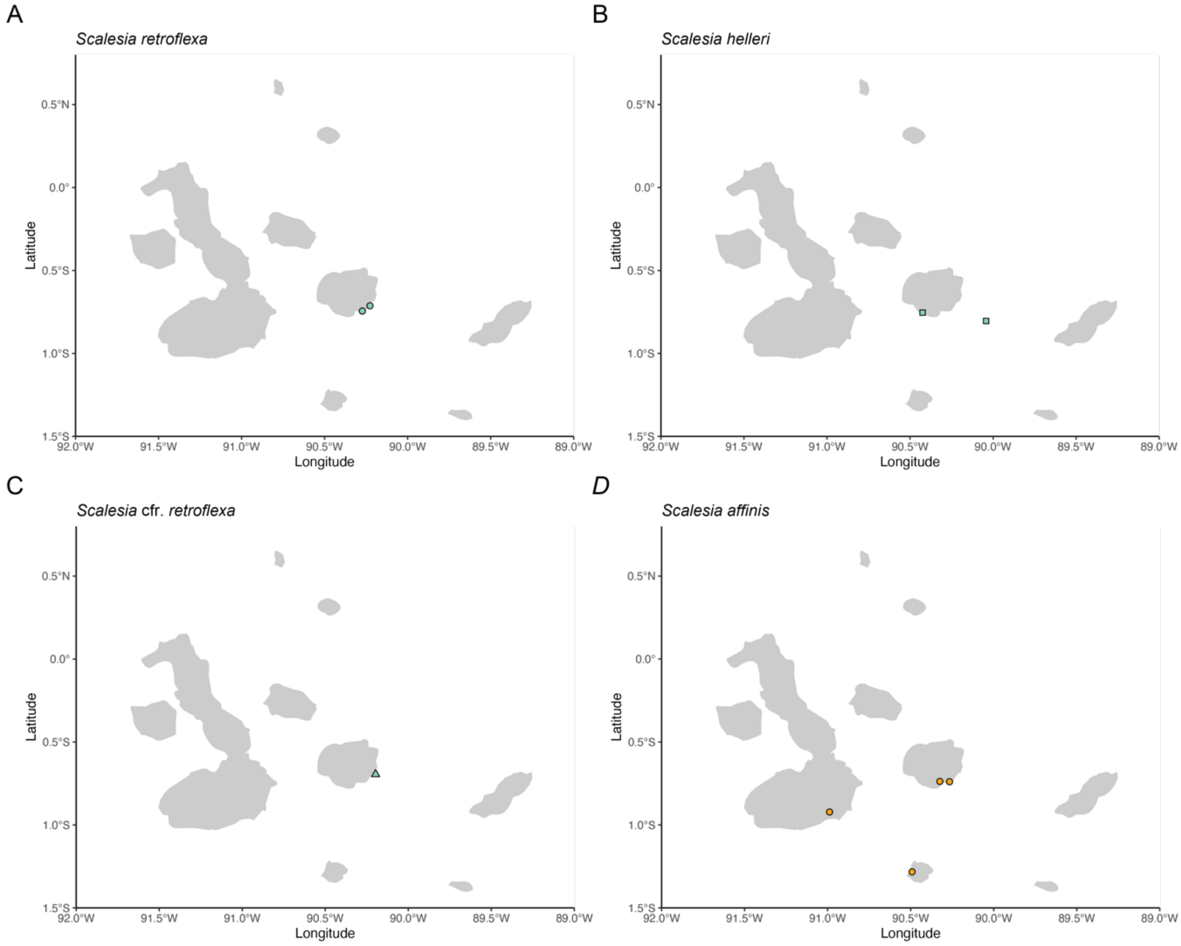

Fig. S4. Sampling locations for clade c-1 species included in the genetic analysis. **A**: *Scalesia retroflexa*. **B**: *Scalesia helleri*. **C**: *Scalesia* cfr. *retroflexa*. **D**: *Scalesia affinis*. Source data are provided on Dryad (https://doi.org/10.5061/dryad.8gtht76rh).

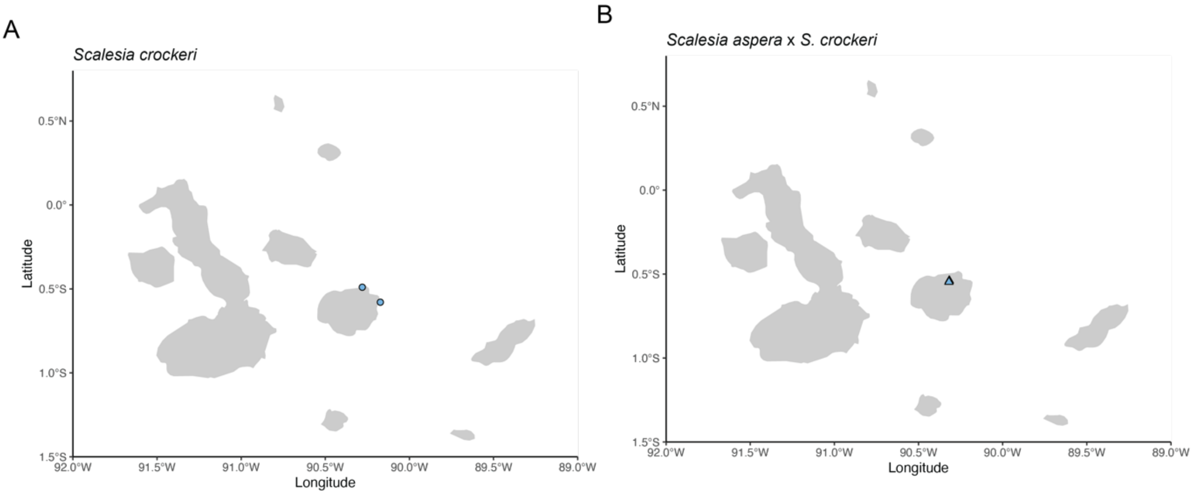

Fig. S5. Sampling locations for clade c-2 species included in the genetic analysis. **A**: *Scalesia crockeri*. **B**: *Scalesia aspera* x *S. crockeri*. Source data are provided on Dryad (https://doi.org/10.5061/dryad.8gtht76rh).

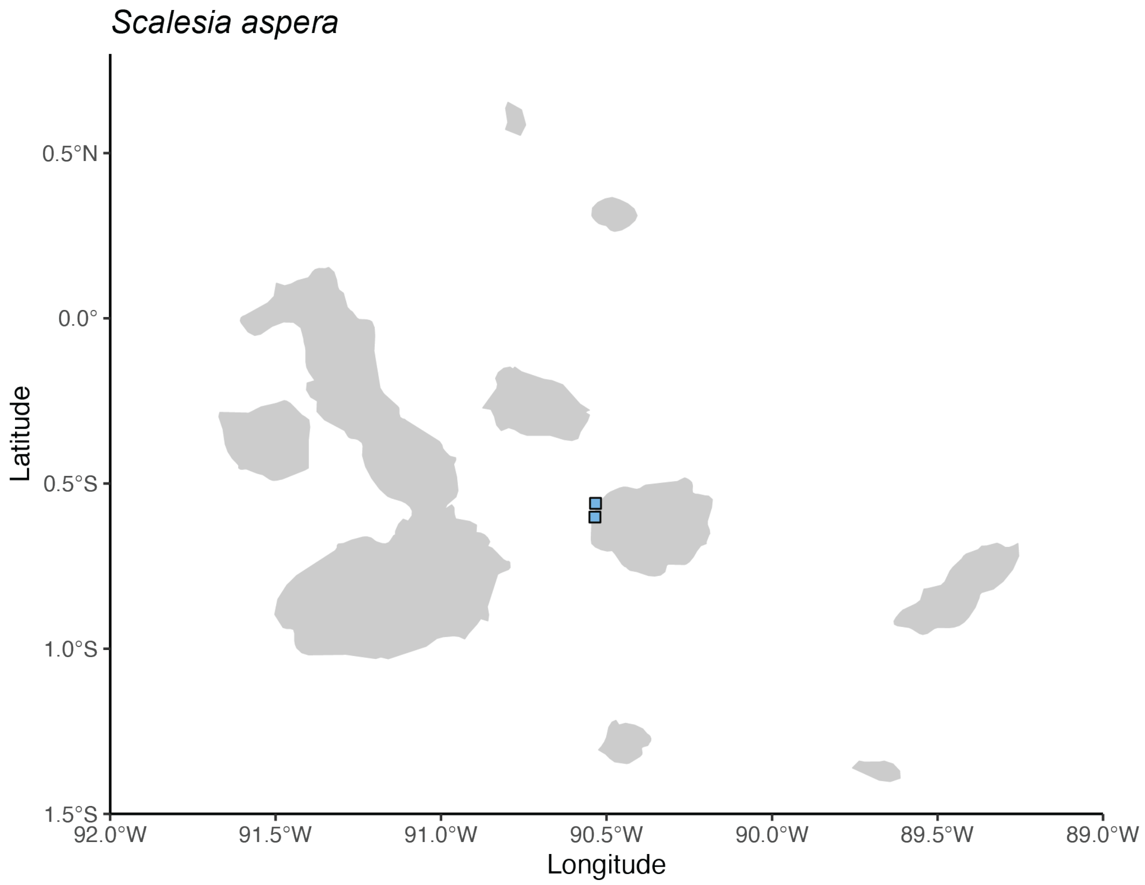

Fig. S6. Sampling locations for the clade c-3 species (*Scalesia aspera*) included in the genetic analysis. Source data are provided on Dryad (https://doi.org/10.5061/dryad.8gtht76rh).

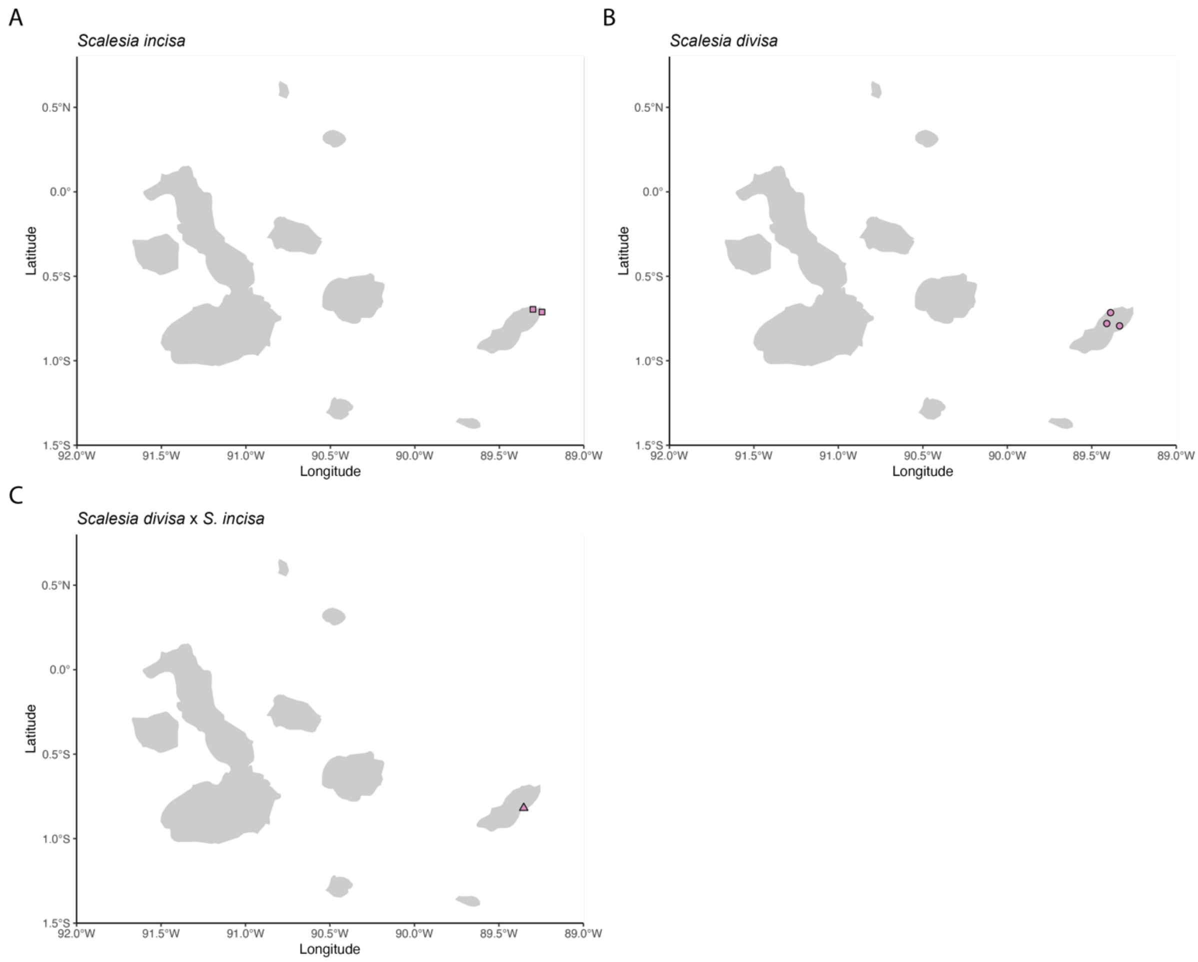

Fig. S7. Sampling locations for clade c-4 species included in the genetic analysis. **A**: *Scalesia incisa*. **B**: *Scalesia divisa*. **C**: *Scalesia divisa* x *S. incisa*. Source data are provided on Dryad (https://doi.org/10.5061/dryad.8gtht76rh).

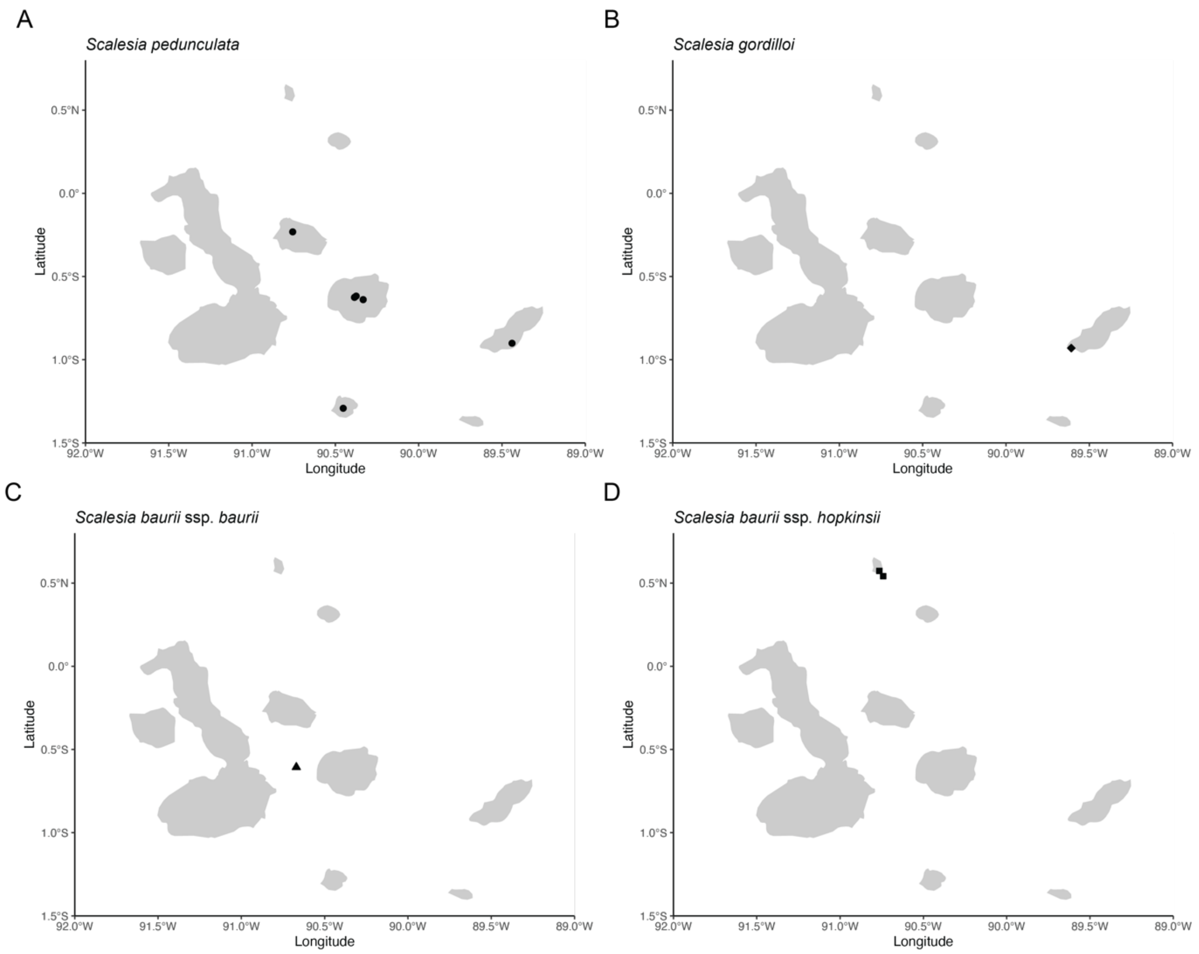

Fig. S8. Sampling locations for clade c-5 species included in the genetic analysis. **A**: *Scalesia pedunculata*. **B**: *Scalesia gordilloi*. **C**: *Scalesia baurii* ssp. *baurii*. **D**: *Scalesia baurii* ssp. *hopkinsii*. Source data are provided on Dryad (https://doi.org/10.5061/dryad.8gtht76rh).

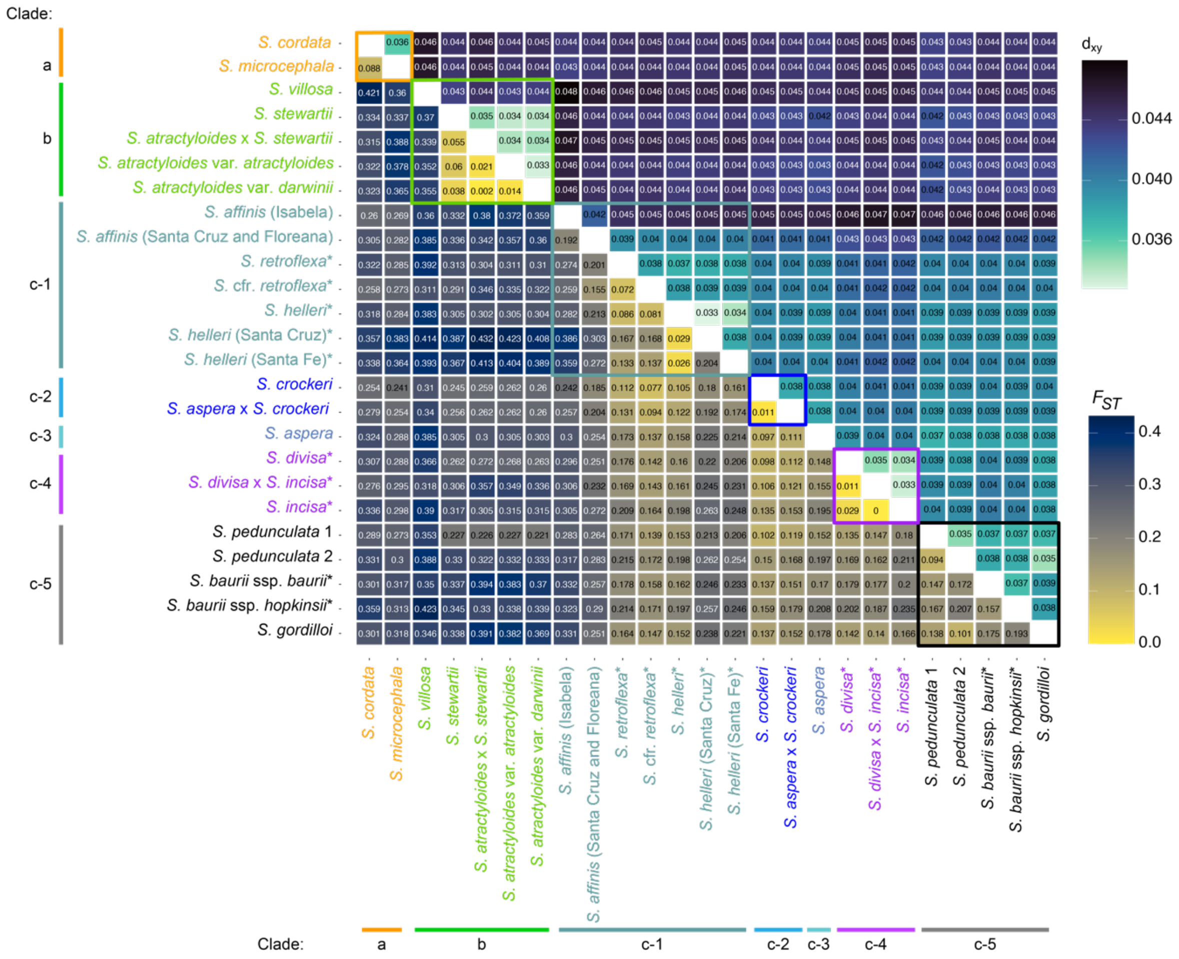

Fig. S9. Divergence between species pairs. Upper diagonal shows d_{xy} at 4-fold degenerate sites and lower diagonal shows genome-wide F_{ST} estimates. Species names are colored according to their clades/subclades (see Fig. 1A). Colored boxes are drawn around comparisons within the same clade/subclade. Tiles are colored according to the F_{ST} or d_{xy} value with yellow or light green being low and dark blue being high values. Species with lobed leaves are marked with an asterisk. Source data are provided on Dryad (https://doi.org/10.5061/dryad.8gtht76rh).

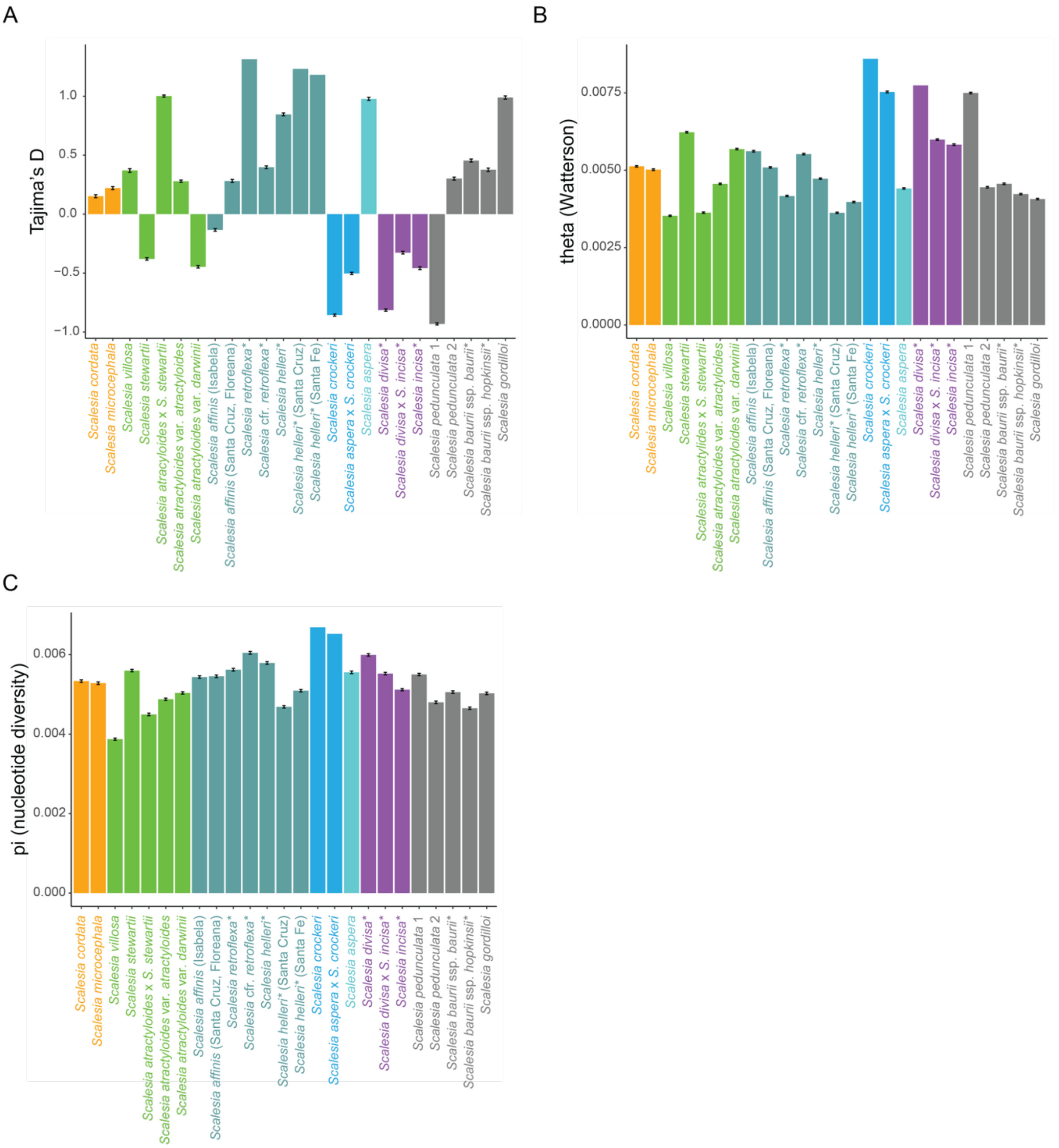

Fig. S10. Tajima's D, theta and nucleotide diversity per species. **A**: Tajima's D. **B**: theta (Watterson). **C**: Nucleotide diversity (pi). **A-C:** Height of the bars indicate the value of the estimators. Bars are coloured based on clade (see Figure 1) with clade a in orange, clade b in green, clade c-1 in petrol, clade c-2 in blue, clade c-3 in turquoise, clade c-4 in purple, and clade c-5 in grey. Error bars indicate the 95% confidence interval (CI) based on 1000 bootstraps. Species with lobed leaves are marked with an asterisk. Source data are provided on Dryad (https://doi.org/10.5061/dryad.8gtht76rh).

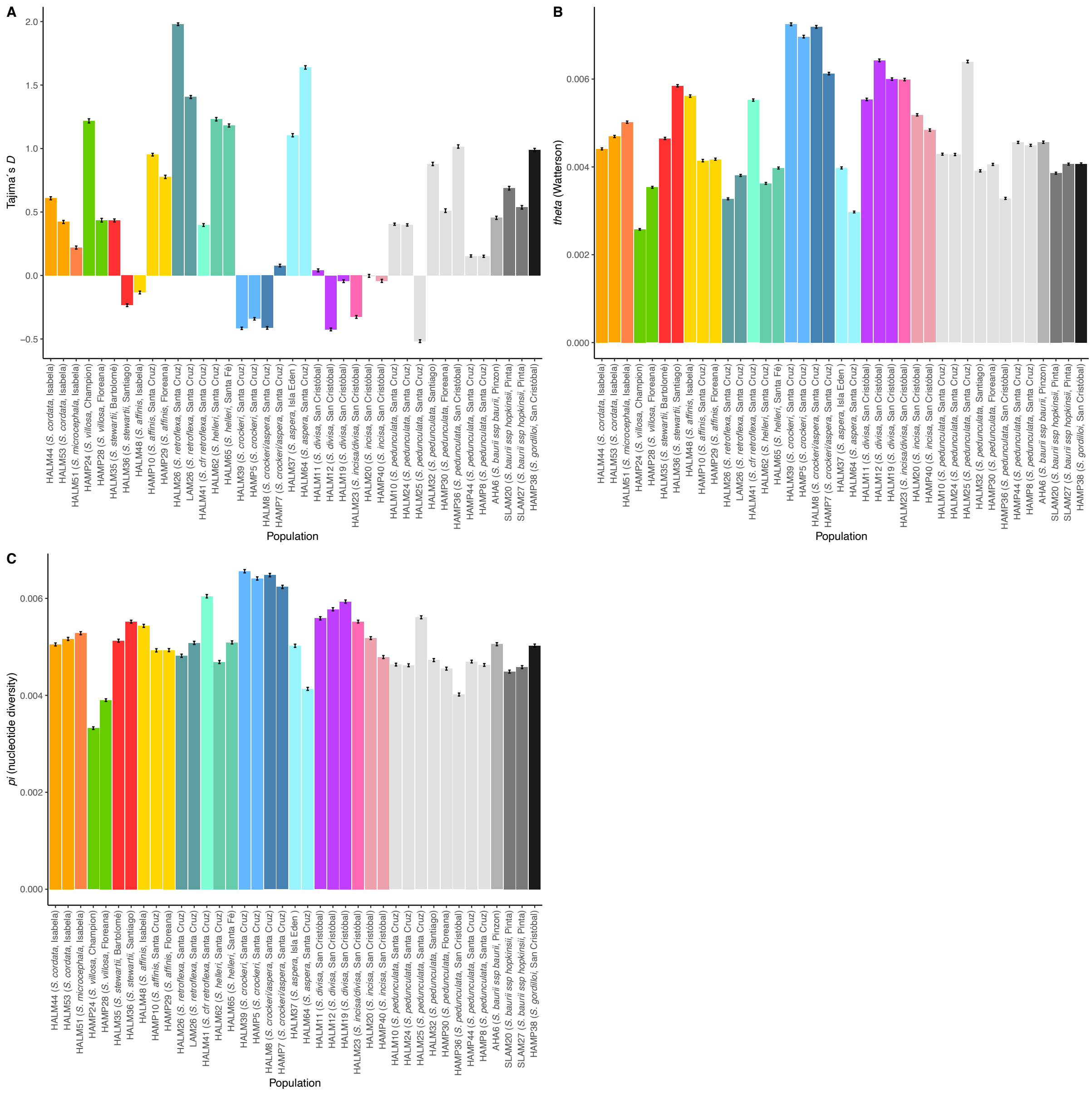

Fig. S11. Tajima's D, theta and nucleotide diversity per population. **A**: Tajima's D. **B**: theta (Watterson). **C**: Nucleotide diversity (pi). **A-C:** Height of the bars indicate the value of the estimators. Bars are coloured based on species. Error bars indicate the 95% confidence interval (CI) based on 1000 bootstraps. Source data are provided on Dryad (https://doi.org/10.5061/dryad.8gtht76rh).

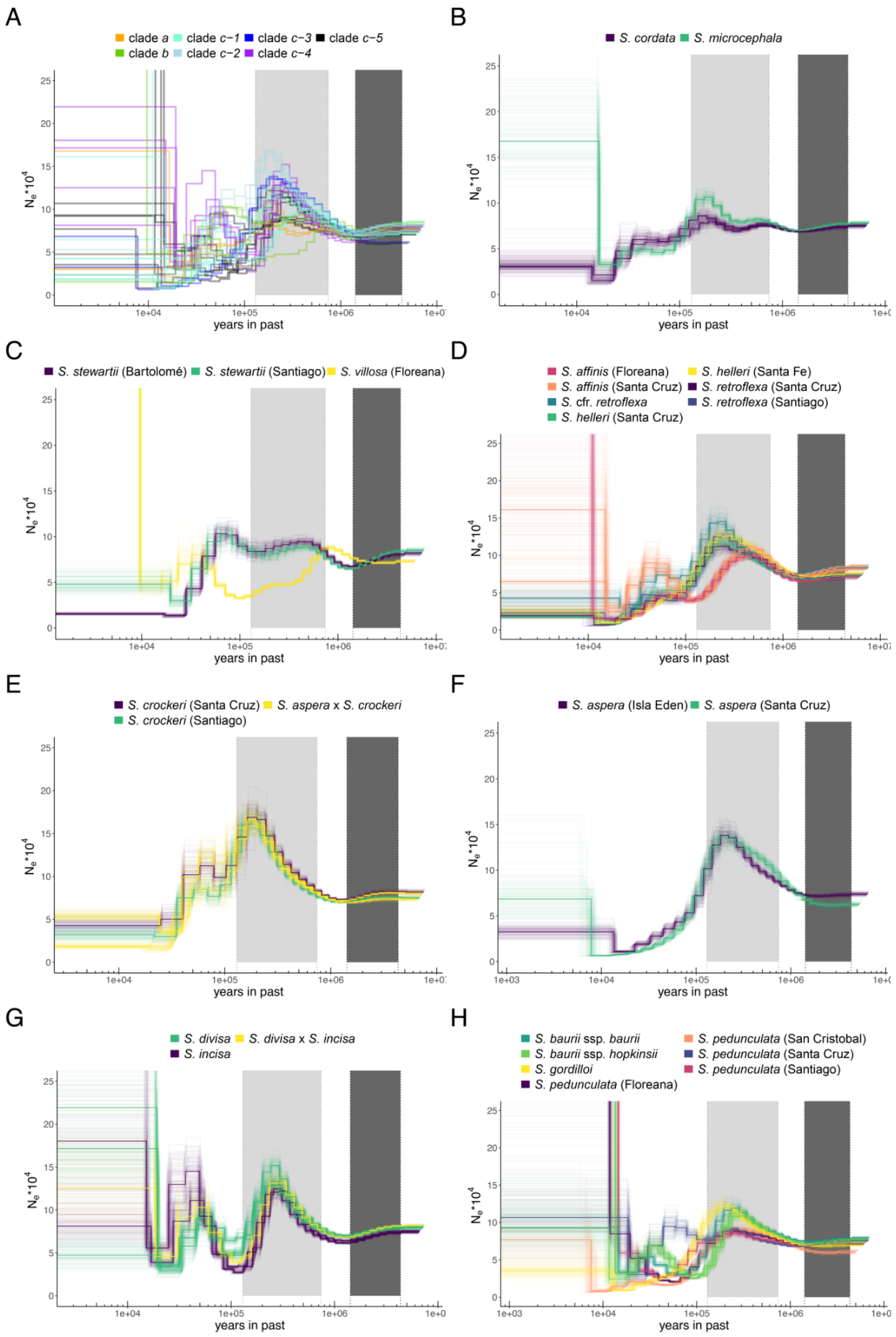

Fig. S12. Demographic reconstructions of past population sizes of higher-coverage (>12 X) *Scalesia* genomes with PSMC. For all samples, a generation time of 3 years and a mutation rate of 6.1×10^{-9} substitutions/site/year[40] * 3 years/generation was used. **A**: all species colored by clade; **B**: clade *a* (*S. cordata, S. microcephala*); **C**: clade *b* (*S. villosa, S. stewartii*); **D**: clade *c-1* (*S. retroflexa*, *S. helleri*, *S.* cfr. *retroflexa, S. aspera*); **E**: clade *c-2* (*S. crockeri, S. aspera* x *S. crockeri*); **F**: clade *c-3* (*S. aspera*); **G**: clade *c-4* (*S. incisa, S. divisa, S.divisa* x *incisa*); **H**: clade *c-5* (*S. baurii* ssp. *baurii*, *S. baurii* ssp. *hopkinsii, S. gordilloi, S. pedunculata*). Dark gray box: estimated age of the *Scalesia* genus, light gray box: estimated time of diversification of extant *Scalesia* species based on[1].

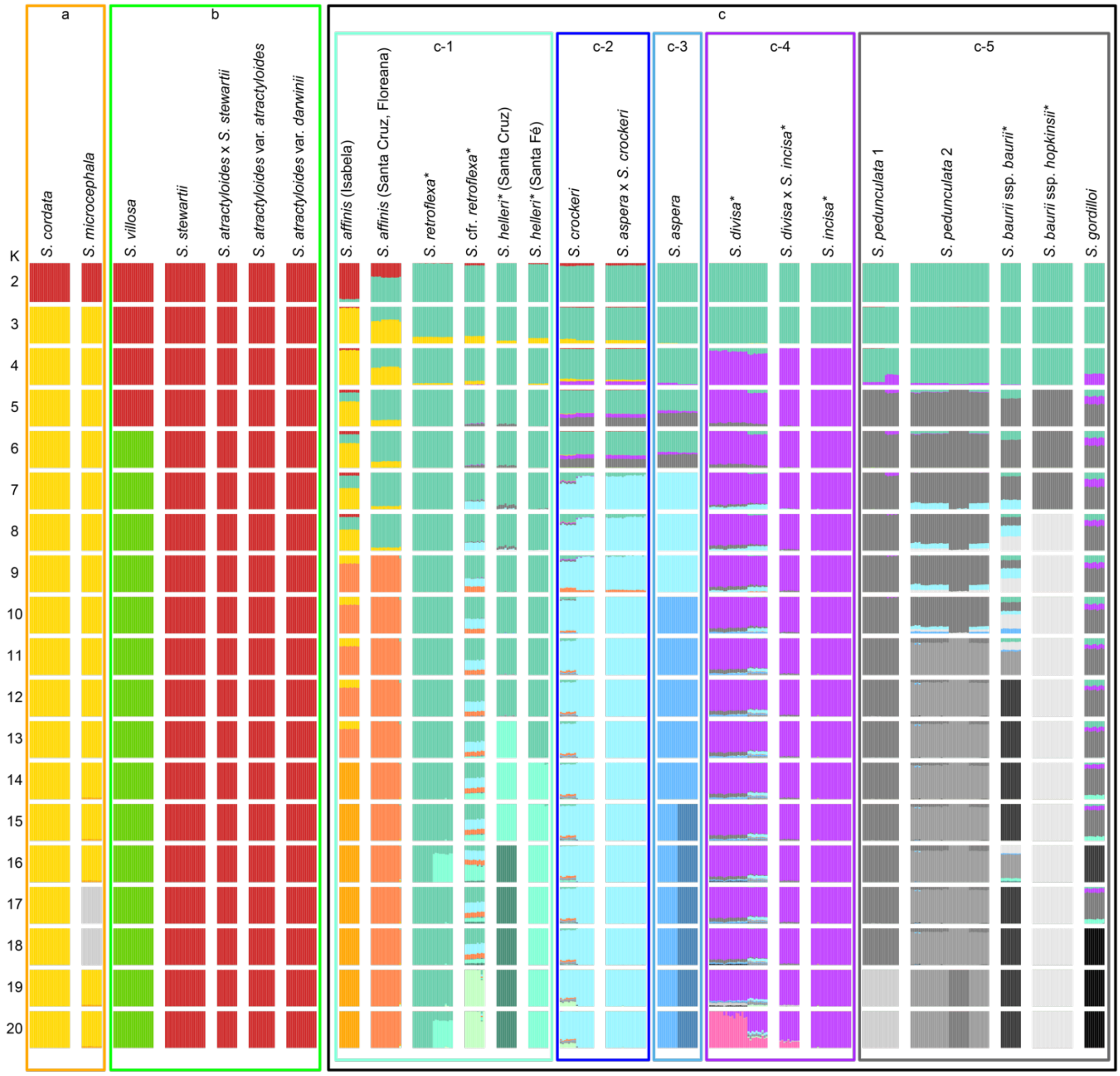

Fig. S13. Admixture barplots for *K*=2 to *K*=20. Each bar represents an individual sample. Colored boxes are drawn around species from the same clade/subclade with the clade name given at the top inside each box. Species with lobed leaves are marked with an asterisk. Source data are provided on Dryad (https://doi.org/10.5061/dryad.8gtht76rh).

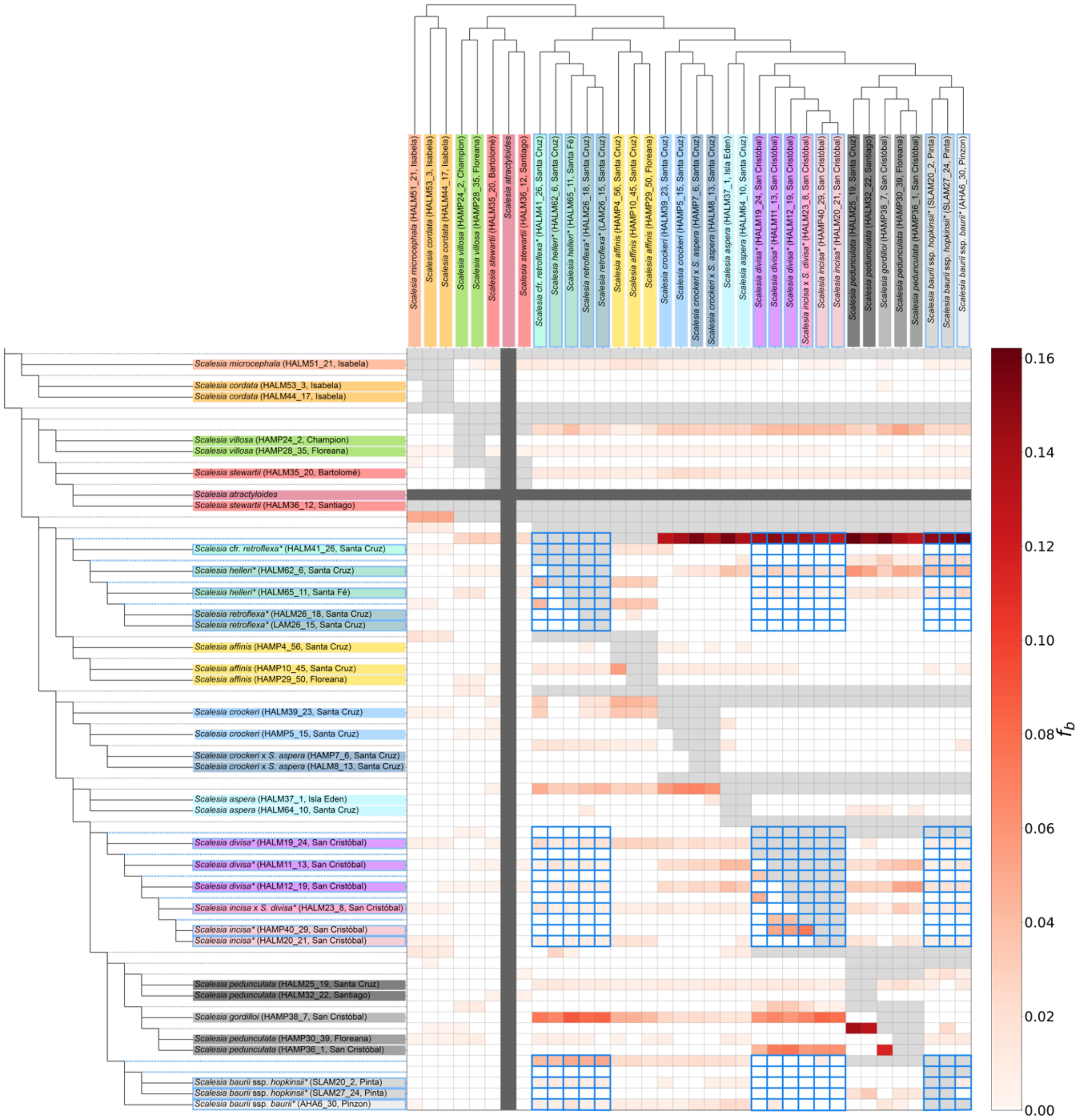

Fig. S14. Presentation of F_{branch} statistics. High values are shown in red and low values in white. Light gray boxes indicate comparisons which are not compatible with the input phylogeny and dark gray boxes indicate comparisons with *S. atractyloides* as it was not included in the F_{branch} analysis. The ASTRAL tree was used as input. Blue boxes indicate comparisons between lobed species. High F_{branch} values indicate excessive allele sharing between two branches. Lobed *Scalesia* species are indicated by blue boxes around the label on the tree. Dotted lines on the left tree indicate ancestral lineages with blue dotted lines indicating those where all descendants are lobed species. Species with lobed leaves are marked with an asterisk. Source data are provided on Dryad (https://doi.org/10.5061/dryad.8gtht76rh).

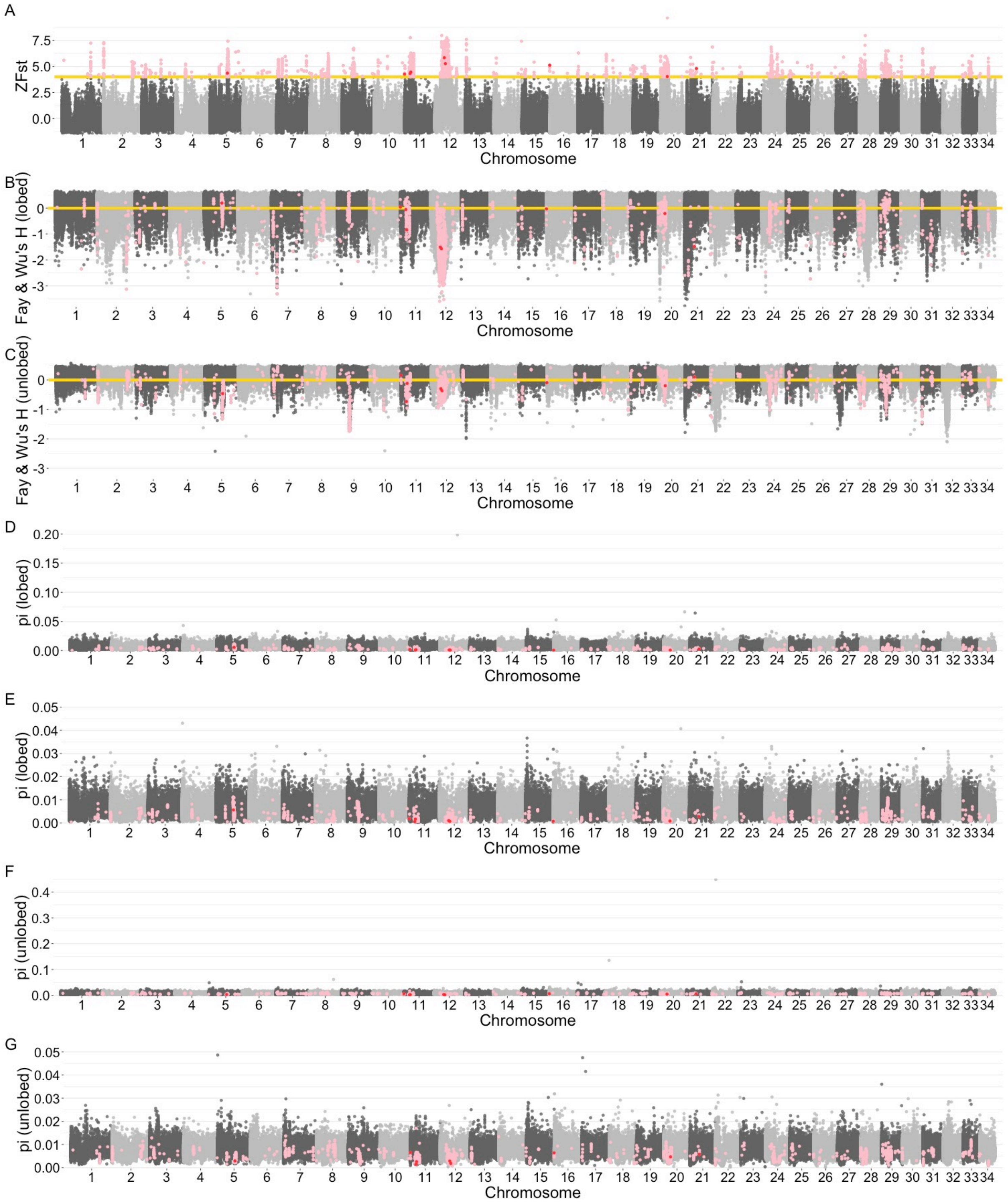

Fig. S15. Manhattan plots of F_{ST} outlier analysis of *S. retroflexa* vs. *S. crockeri* in non-overlapping 10-kbp sliding windows. F_{ST} outlier windows ($ZF_{ST} \geq 4$) are shown in pink. Outlier windows overlapping with a gene associated with leaf development are shown in red. **A**: ZF_{ST} values for each window. Yellow line indicates the threshold for defining outlier windows ($ZF_{ST} = 4$). **B**: Fay and Wu's *H* for *S. retroflexa*. Yellow line: *H*=0. **C**: Fay and Wu's *H* for *S. crockeri*. Yellow line: *H*=0. **D**: Nucleotide diversity for *S. retroflexa*. **E**: Nucleotide diversity for *S. retroflexa* with upper y-axis limited to 0.05. **F**: Nucleotide diversity for *S. crockeri*. **G**: Nucleotide diversity for *S. crockeri* with upper y-axis limited to 0.05. Source data are provided on Dryad (https://doi.org/10.5061/dryad.8gtht76rh).

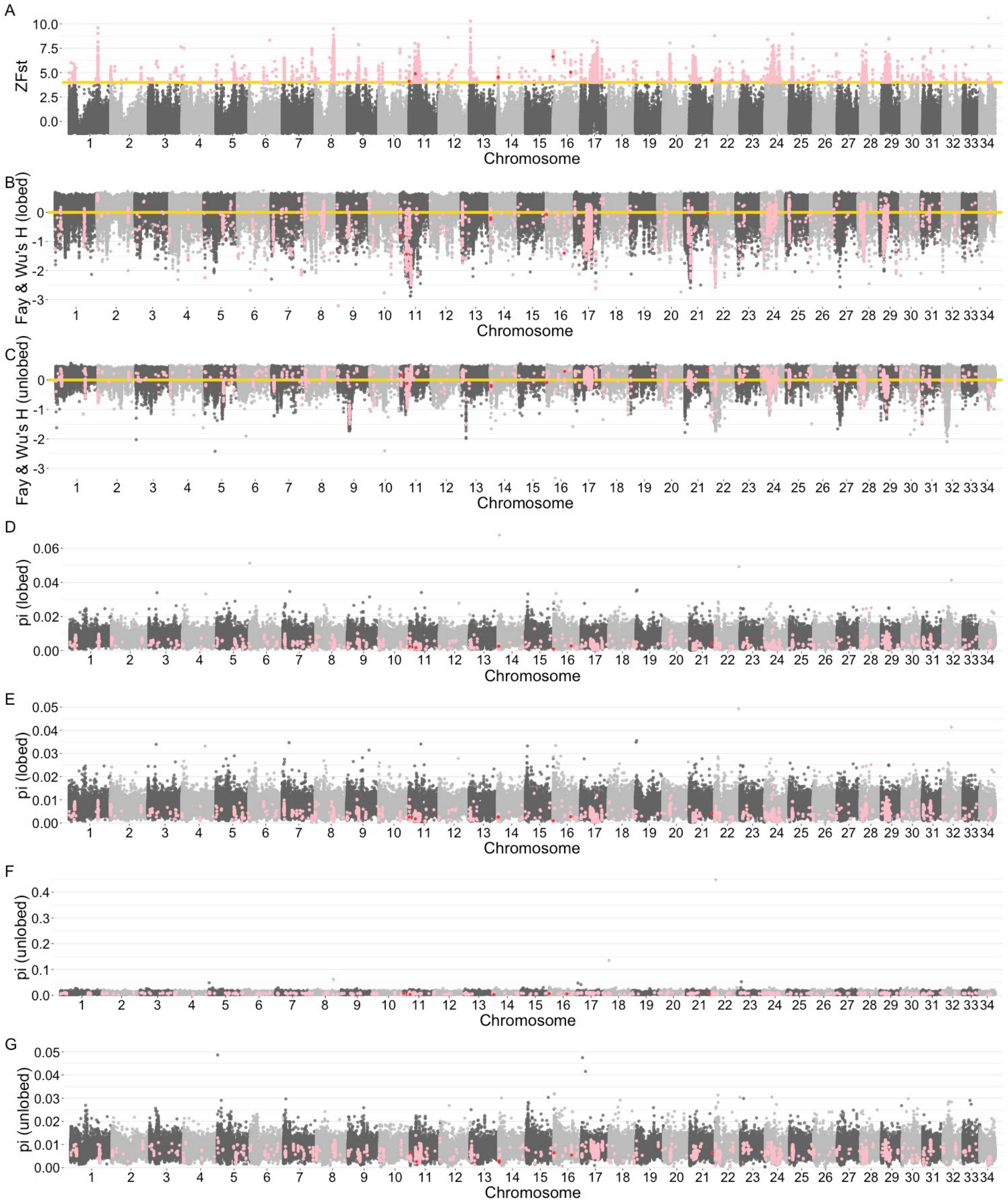

Fig. S16. Manhattan plots of F_{ST} outlier analysis of *S.* cfr. *retroflexa* vs. *S. crockeri* in non-overlapping 10-kbp sliding windows. F_{ST} outlier windows ($ZF_{ST} \geq 4$) are shown in pink. Outlier windows overlapping with a gene associated with leaf development are shown in red. **A**: ZF_{ST} values for each window. Yellow line indicates the threshold for defining outlier windows (ZF_{ST} = 4). **B**: Fay and Wu's *H* for *S.* cfr. *retroflexa*. Yellow line: *H*=0. **C**: Fay and Wu's *H* for *S. crockeri*. Yellow line: *H*=0. **D**: Nucleotide diversity for *S.* cfr. *retroflexa*. **E**: Nucleotide diversity for *S.* cfr. *retroflexa* with upper y-axis limited to 0.05. **F**: Nucleotide diversity for *S. crockeri*. **G**: Nucleotide diversity for *S. crockeri* with upper y-axis limited to 0.05. Source data are provided on Dryad (https://doi.org/10.5061/dryad.8gtht76rh).

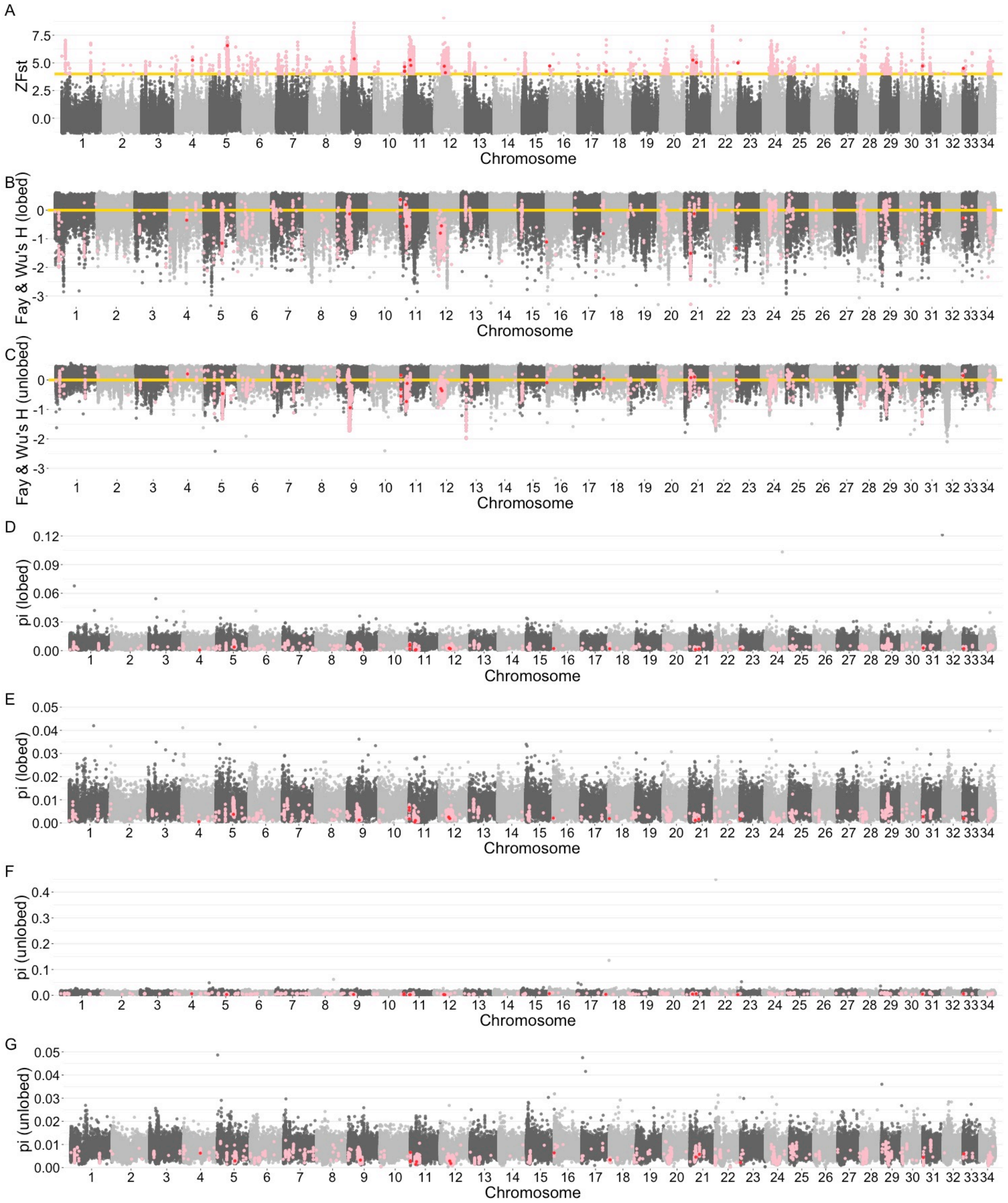

Fig. S17. Manhattan plots of F_{ST} outlier analysis of *S. helleri* vs. *S. crockeri* in non-overlapping 10-kbp sliding windows. F_{ST} outlier windows ($ZF_{ST} \geq 4$) are shown in pink. Outlier windows overlapping with a gene associated with leaf development are shown in red. **A**: ZF_{ST} values for each window. Yellow line indicates the threshold for defining outlier windows ($ZF_{ST} = 4$). **B**: Fay and Wu's *H* for *S. helleri*. Yellow line: *H*=0. **C**: Fay and Wu's *H* for *S. crockeri*. Yellow line: *H*=0. **D**: Nucleotide diversity for *S. helleri*. **E**: Nucleotide diversity for *S. helleri* with upper y-axis limited to 0.05. **F**: Nucleotide diversity for *S. crockeri*. **G**: Nucleotide diversity for *S. crockeri* with upper y-axis limited to 0.05. Source data are provided on Dryad (https://doi.org/10.5061/dryad.8gtht76rh).

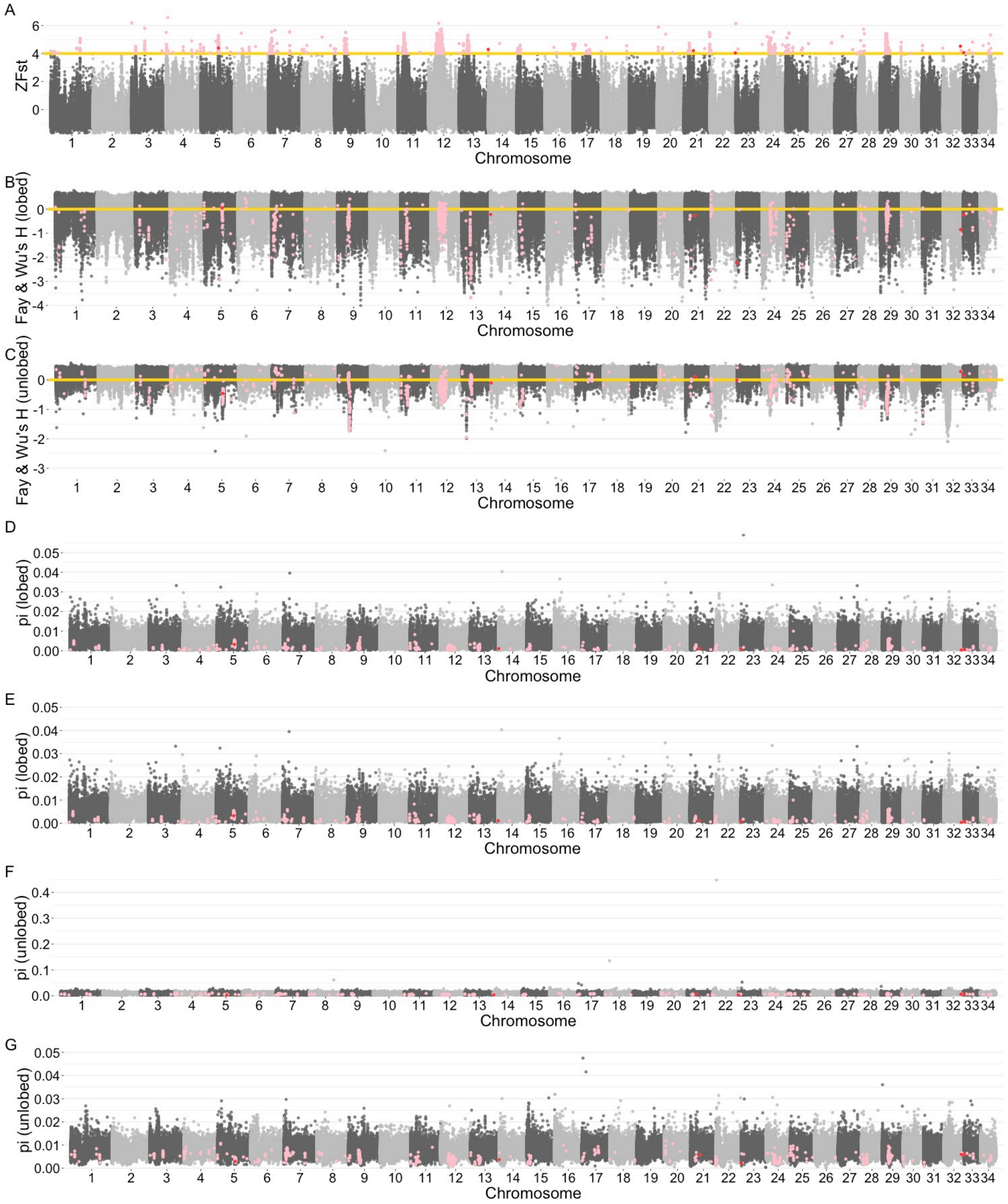

Fig. S18. Manhattan plots of F_{ST} outlier analysis of *S. helleri* (Santa Cruz population) vs. *S. crockeri* in non-overlapping 10-kbp sliding windows. F_{ST} outlier windows ($ZF_{ST} \geq 4$) are shown in pink. Outlier windows overlapping with a gene associated with leaf development are shown in red. **A**: ZF_{ST} values for each window. Yellow line indicates the threshold for defining outlier windows ($ZF_{ST} = 4$). **B**: Fay and Wu's *H* for *S. helleri* (Santa Cruz population). Yellow line: H=0. **C**: Fay and Wu's *H* for *S. crockeri*. Yellow line: H=0. **D**: Nucleotide diversity for *S. helleri* (Santa Cruz population). **E**: Nucleotide diversity for *S. helleri* (Santa Cruz population) with upper y-axis limited to 0.05. **F**: Nucleotide diversity for *S. crockeri*. **G**: Nucleotide diversity for *S. crockeri* with upper y-axis limited to 0.05. Source data are provided on Dryad (https://doi.org/10.5061/dryad.8gtht76rh).

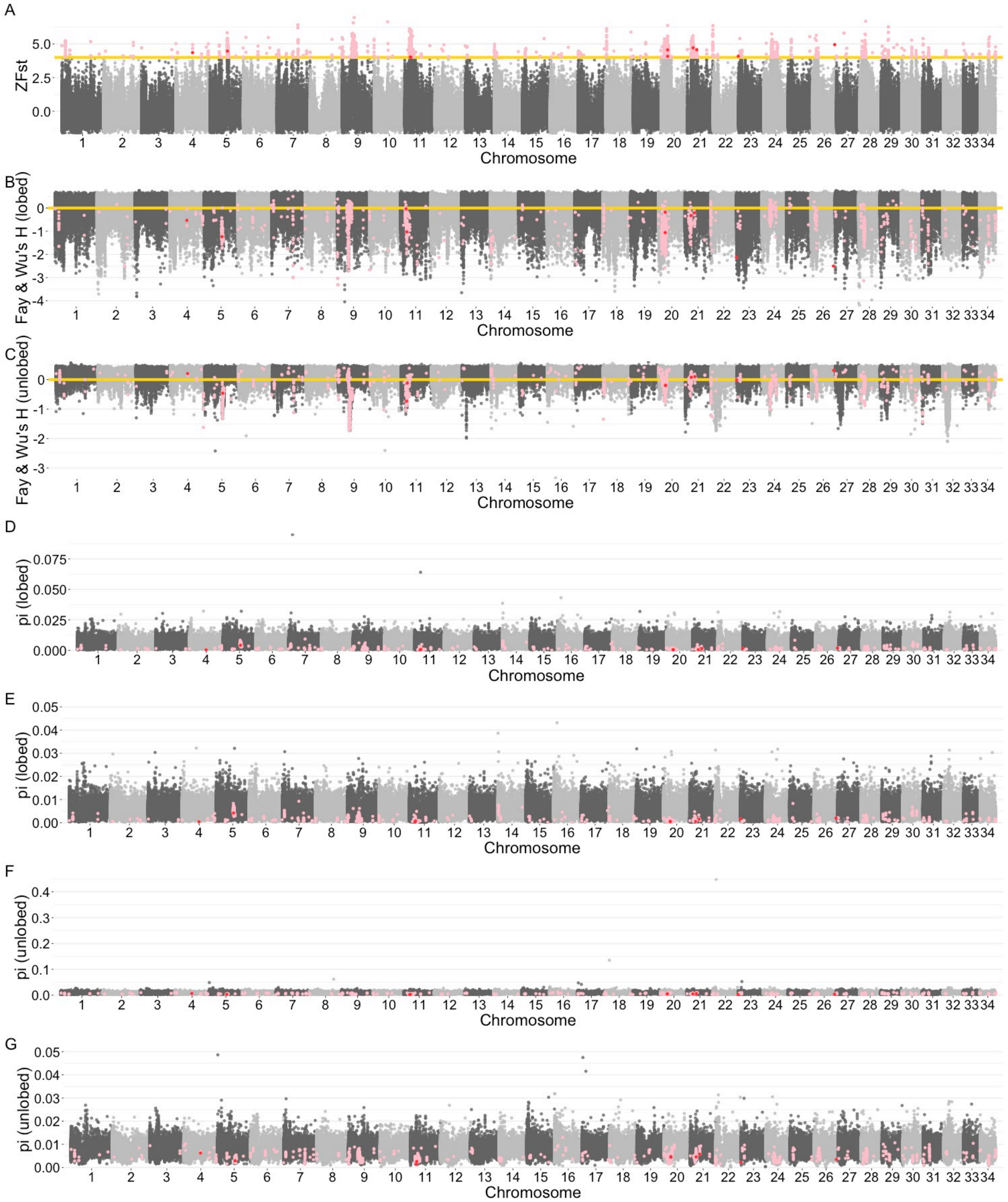

Fig. S19. Manhattan plots of F_{ST} outlier analysis of *S. helleri* (Santa Fe population) vs. *S. crockeri* in non-overlapping 10-kbp sliding windows. F_{ST} outlier windows ($ZF_{ST} \geq 4$) are shown in pink. Outlier windows overlapping with a gene associated with leaf development are shown in red. **A**: ZF_{ST} values for each window. Yellow line indicates the threshold for defining outlier windows ($ZF_{ST} = 4$). **B**: Fay and Wu's H for *S. helleri* (Santa Fe population). Yellow line: H=0. **C**: Fay and Wu's H for *S. crockeri*. Yellow line: H=0. **D**: Nucleotide diversity for *S. helleri* (Santa Fe population). **E**: Nucleotide diversity for *S. helleri* (Santa Fe population) with upper y-axis limited to 0.05. **F**: Nucleotide diversity for *S. crockeri*. **G**: Nucleotide diversity for *S. crockeri* with upper y-axis limited to 0.05. Source data are provided on Dryad (https://doi.org/10.5061/dryad.8gtht76rh).

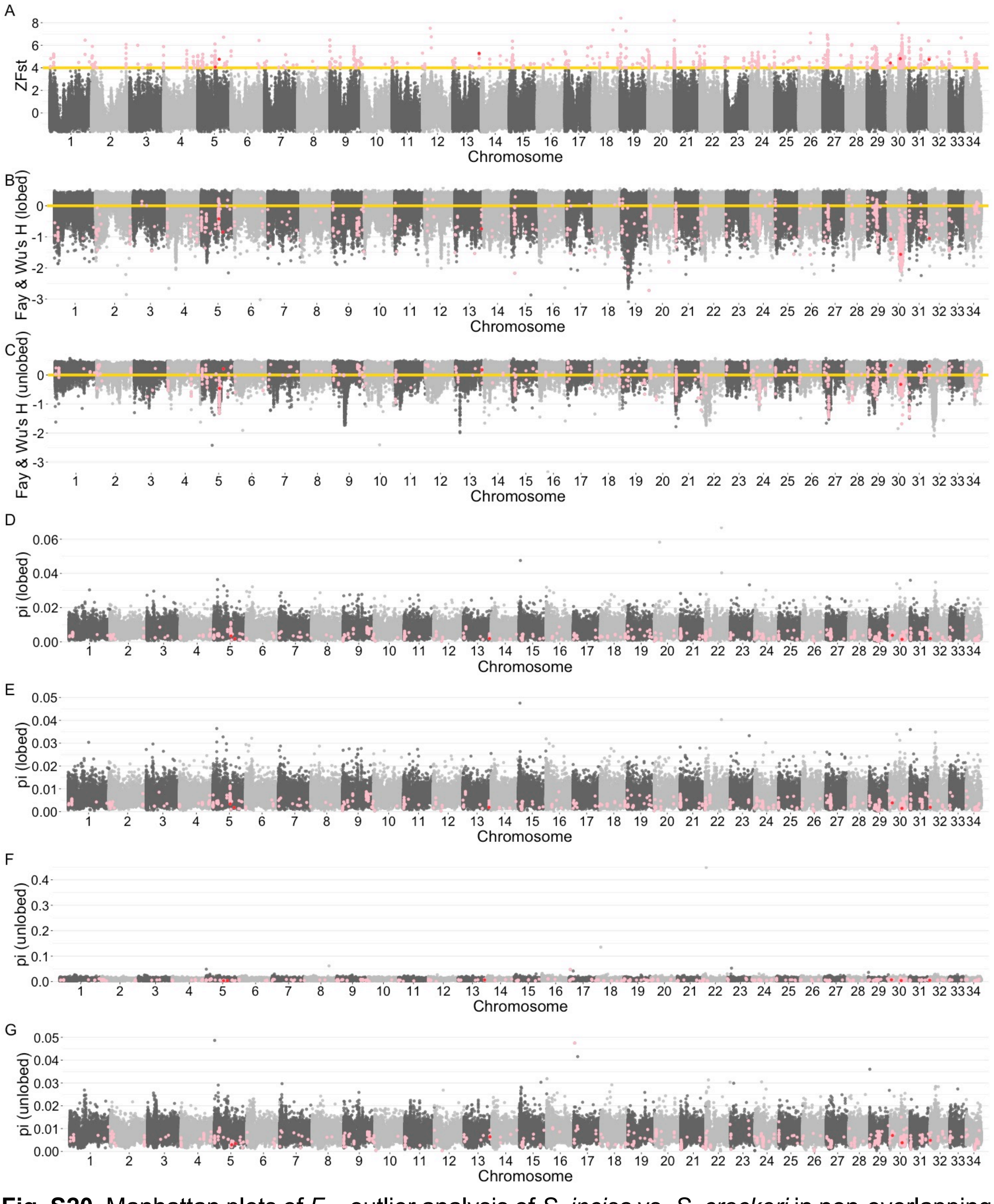

Fig. S20. Manhattan plots of F_{ST} outlier analysis of *S. incisa* vs. *S. crockeri* in non-overlapping 10-kbp sliding windows. F_{ST} outlier windows ($ZF_{ST} \geq 4$) are shown in pink. Outlier windows overlapping with a gene associated with leaf development are shown in red. **A**: ZF_{ST} values for each window. Yellow line indicates the threshold for defining outlier windows ($ZF_{ST} = 4$). **B**: Fay and Wu's *H* for *S. incisa*. Yellow line: H=0. **C**: Fay and Wu's *H* for *S. crockeri*. Yellow line: H=0. **D**: Nucleotide diversity for *S. incisa*. **E**: Nucleotide diversity for *S. incisa* with upper y-axis limited to 0.05. **F**: Nucleotide diversity for *S. crockeri*. **G**: Nucleotide diversity for *S. crockeri* with upper y-axis limited to 0.05. Source data are provided on Dryad (https://doi.org/10.5061/dryad.8gtht76rh).

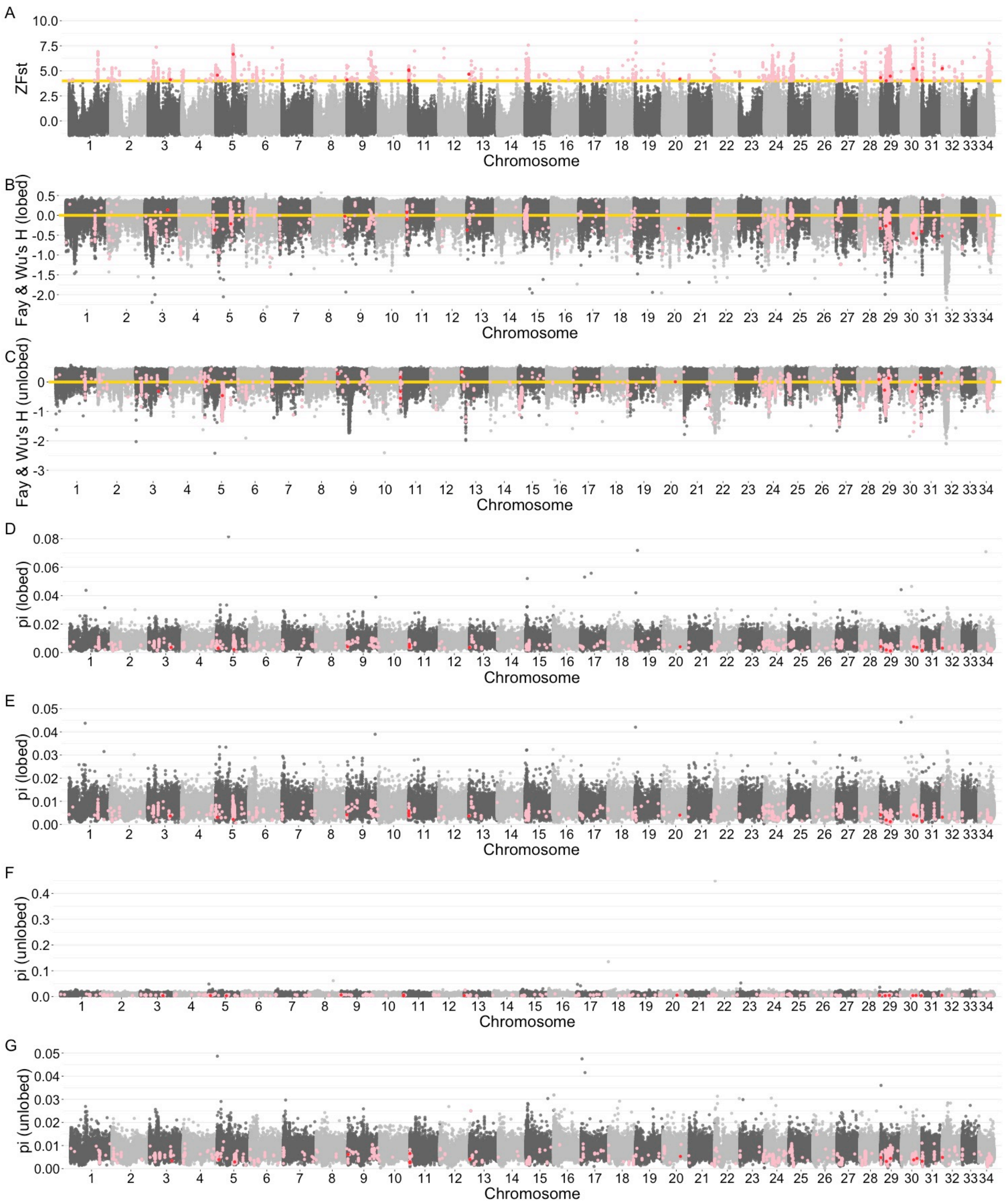

Fig. S21. Manhattan plots of F_{ST} outlier analysis of *S. divisa* vs. *S. crockeri* in non-overlapping 10-kbp sliding windows. F_{ST} outlier windows ($ZF_{ST} \geq 4$) are shown in pink. Outlier windows overlapping with a gene associated with leaf development are shown in red. **A**: ZF_{ST} values for each window. Yellow line indicates the threshold for defining outlier windows ($ZF_{ST} = 4$). **B**: Fay and Wu's *H* for *S. divisa*. Yellow line: *H*=0. **C**: Fay and Wu's *H* for *S. crockeri*. Yellow line: *H*=0. **D**: Nucleotide diversity for *S. divisa*. **E**: Nucleotide diversity for *S. divisa* with upper y-axis limited to 0.05. **F**: Nucleotide diversity for *S. crockeri*. **G**: Nucleotide diversity for *S. crockeri* with upper y-axis limited to 0.05. Source data are provided on Dryad (https://doi.org/10.5061/dryad.8gtht76rh).

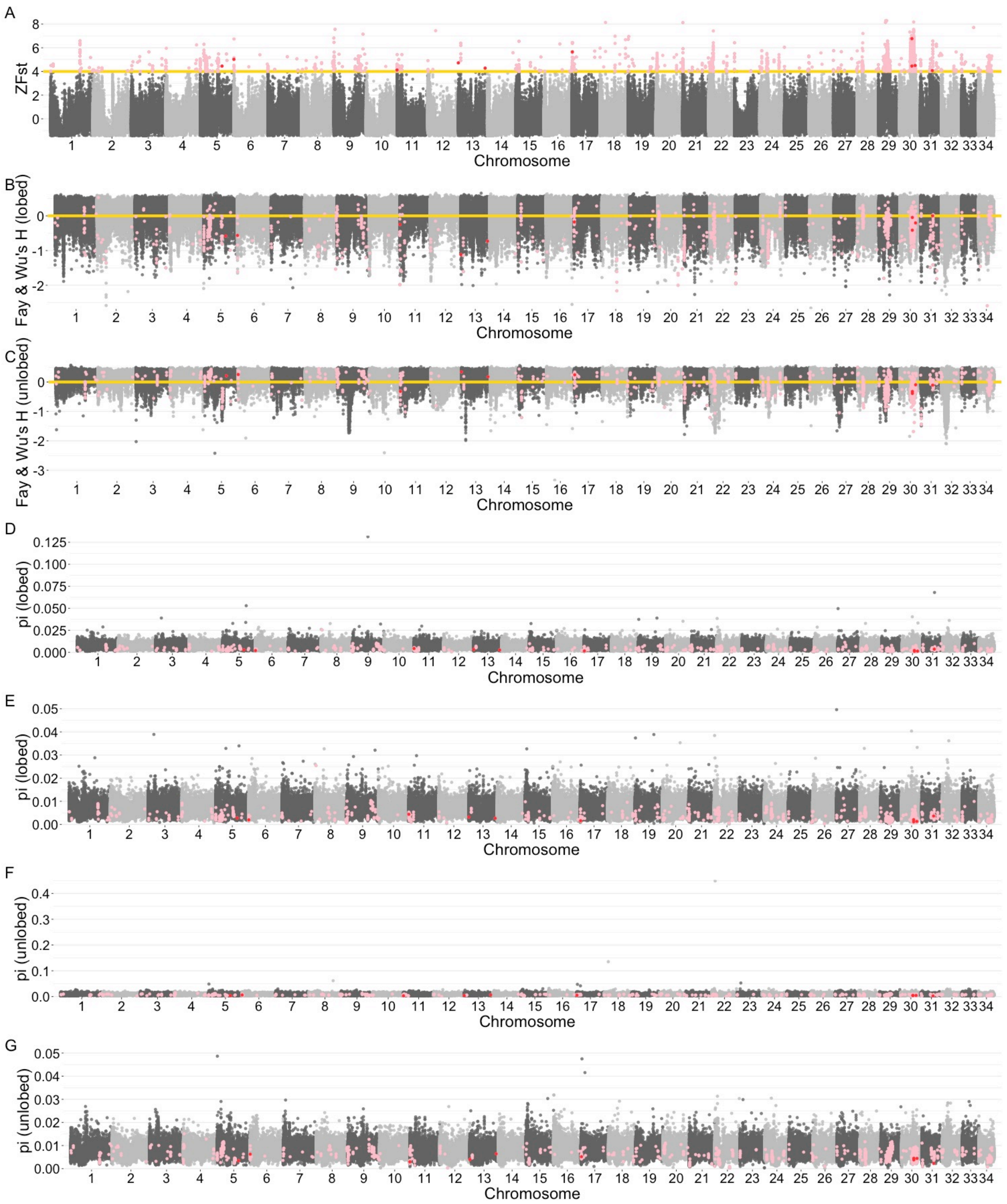

Fig. S22. Manhattan plots of F_{ST} outlier analysis of *S. divisa* x *S. incisa* vs. *S. crockeri* in non-overlapping 10-kbp sliding windows. F_{ST} outlier windows ($ZF_{ST} \geq 4$) are shown in pink. Outlier windows overlapping with a gene associated with leaf development are shown in red. **A**: ZF_{ST} values for each window. Yellow line indicates the threshold for defining outlier windows ($ZF_{ST} = 4$). **B**: Fay and Wu's *H* for *S. divisa* x *S. incisa*. Yellow line: *H*=0. **C**: Fay and Wu's *H* for *S. crockeri*. Yellow line: *H*=0. **D**: Nucleotide diversity for *S. divisa* x *S. incisa*. **E**: Nucleotide diversity for *S. divisa* x *S. incisa* with upper y-axis limited to 0.05. **F**: Nucleotide diversity for *S. crockeri*. **G**: Nucleotide diversity for *S. crockeri* with upper y-axis limited to 0.05. Source data are provided on Dryad (https://doi.org/10.5061/dryad.8gtht76rh).

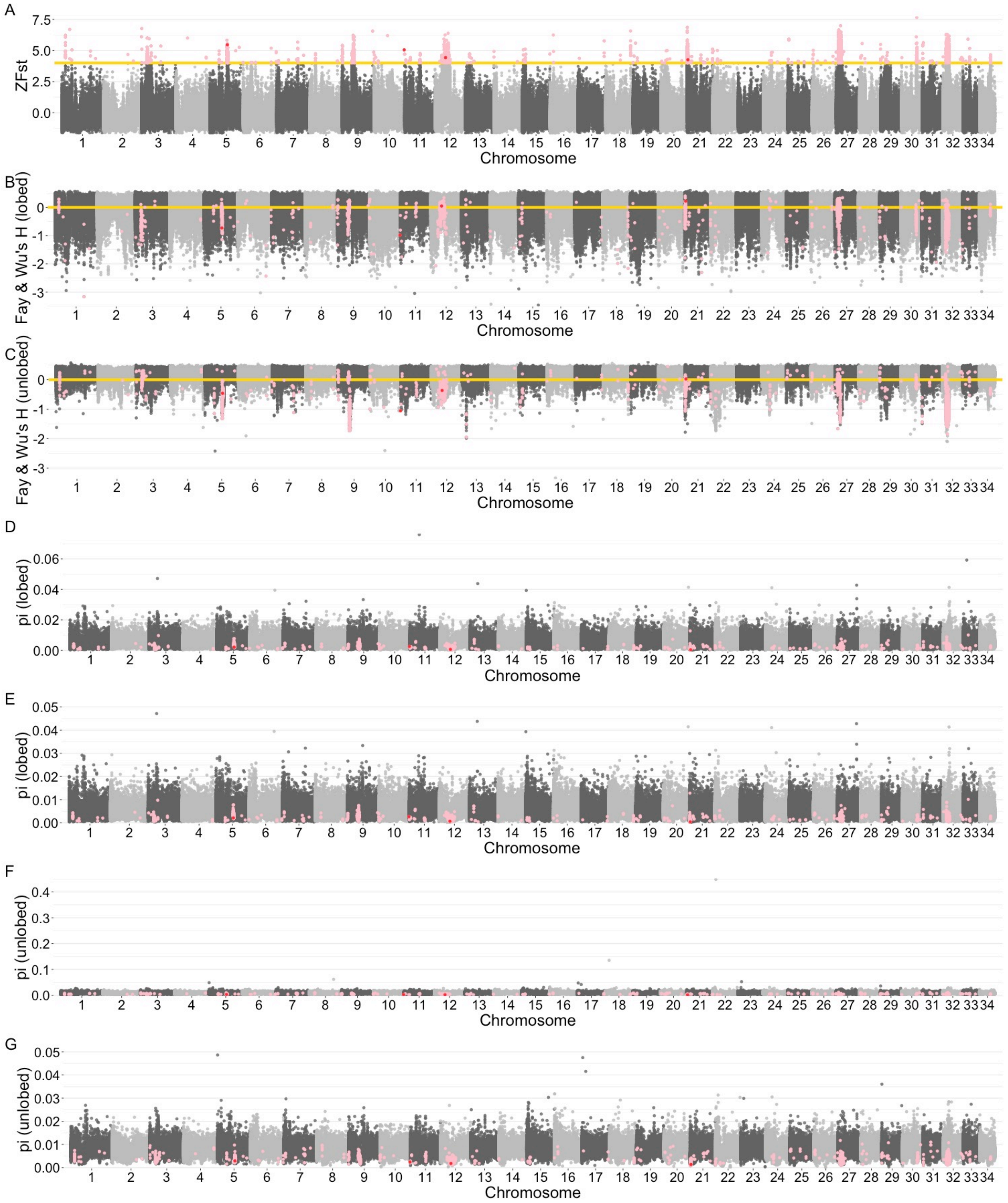

Fig. S23. Manhattan plots of F_{ST} outlier analysis of *S. baurii* ssp. *hopkinsii* vs. *S. crockeri* in non-overlapping 10-kbp sliding windows. F_{ST} outlier windows ($ZF_{ST} \geq 4$) are shown in pink. Outlier windows overlapping with a gene associated with leaf development are shown in red. **A**: ZF_{ST} values for each window. Yellow line indicates the threshold for defining outlier windows (ZF_{ST} = 4). **B**: Fay and Wu's H for *S. baurii* ssp. *hopkinsii*. Yellow line: *H*=0. **C**: Fay and Wu's *H* for *S. crockeri*. Yellow line: *H*=0. **D**: Nucleotide diversity for *S. baurii* ssp. *hopkinsii*. **E**: Nucleotide diversity for *S. baurii* ssp. *hopkinsii* with upper y-axis limited to 0.05. **F**: Nucleotide diversity for *S. crockeri*. **G**: Nucleotide diversity for *S. crockeri* with upper y-axis limited to 0.05. Source data are provided on Dryad (https://doi.org/10.5061/dryad.8gtht76rh).

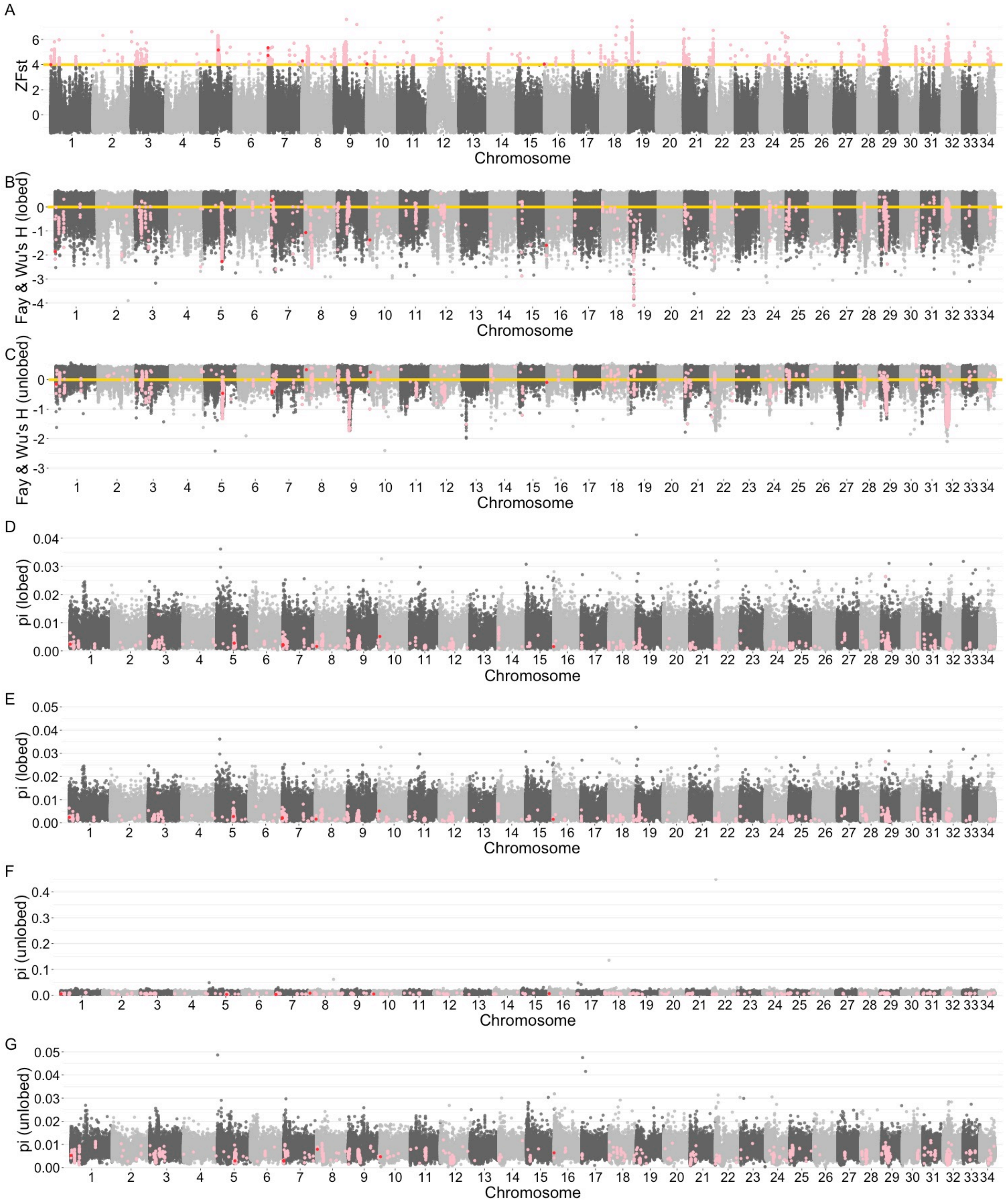

Fig. S24. Manhattan plots of F_{ST} outlier analysis of *S. baurii* ssp. *baurii* vs. *S. crockeri* in non-overlapping 10-kbp sliding windows. F_{ST} outlier windows ($ZF_{ST} \geq 4$) are shown in pink. Outlier windows overlapping with a gene associated with leaf development are shown in red. **A**: ZF_{ST} values for each window. Yellow line indicates the threshold for defining outlier windows (ZF_{ST} = 4). **B**: Fay and Wu's *H* for *S. baurii* ssp. *baurii*. Yellow line: *H*=0. **C**: Fay and Wu's *H* for *S. crockeri*. Yellow line: *H*=0. **D**: Nucleotide diversity for *S. baurii* ssp. *baurii*. **E**: Nucleotide diversity for *S. baurii* ssp. *baurii* with upper y-axis limited to 0.05. **F**: Nucleotide diversity for *S. crockeri*. **G**: Nucleotide diversity for *S. crockeri* with upper y-axis limited to 0.05. Source data are provided on Dryad (https://doi.org/10.5061/dryad.8gtht76rh).

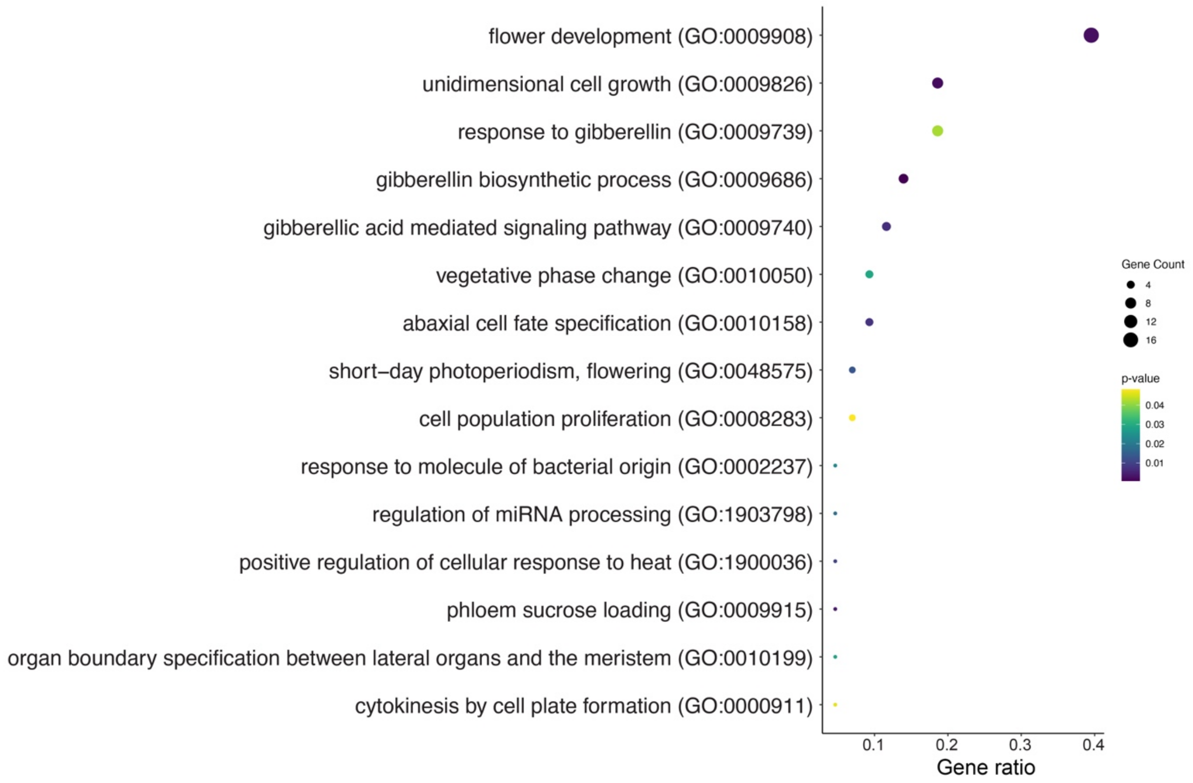

Fig. S25. GO enrichment analysis of putatively selected leaf development genes in *Scalesia* species with lobed leaves. The test was performed with the list of genes associated with leaf development (1630 genes) as a null list. Gene ratio represents the number of selected leaf development genes (gene count) associated with a given GO term divided by the total number of selected leaf development genes (43). Source data are provided on Dryad (https://doi.org/10.5061/dryad.8gtht76rh).

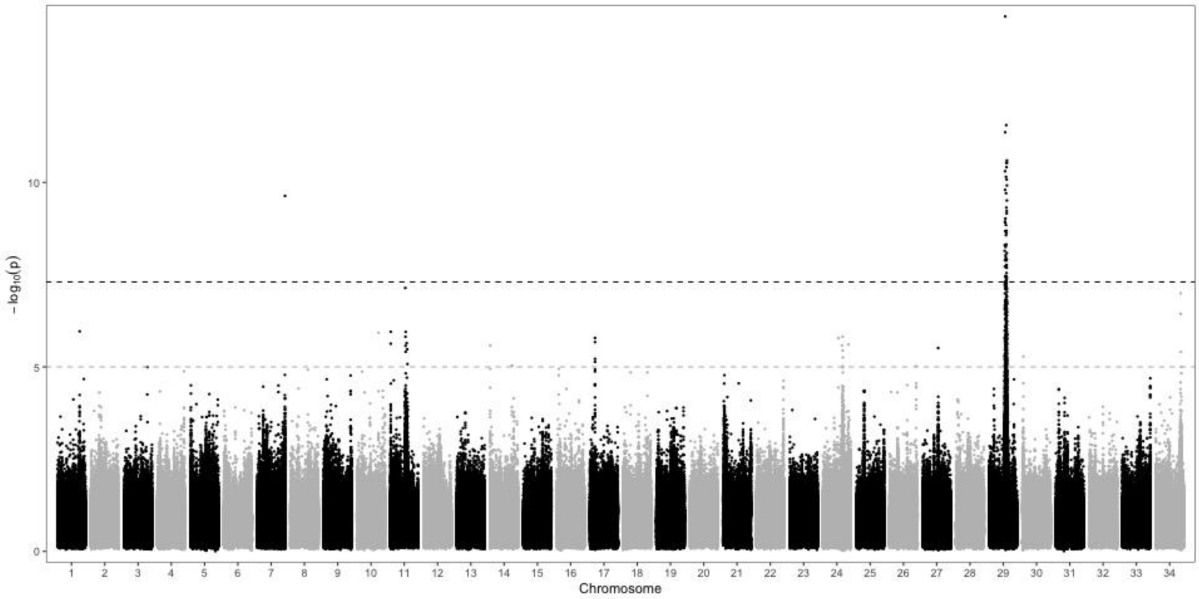

Fig. S26. Manhattan plot for GWAS analysis using samples from lobed and unlobed species within clade *c*. Black dotted line indicates the significance threshold (*p-value* ≥ 5e-08).

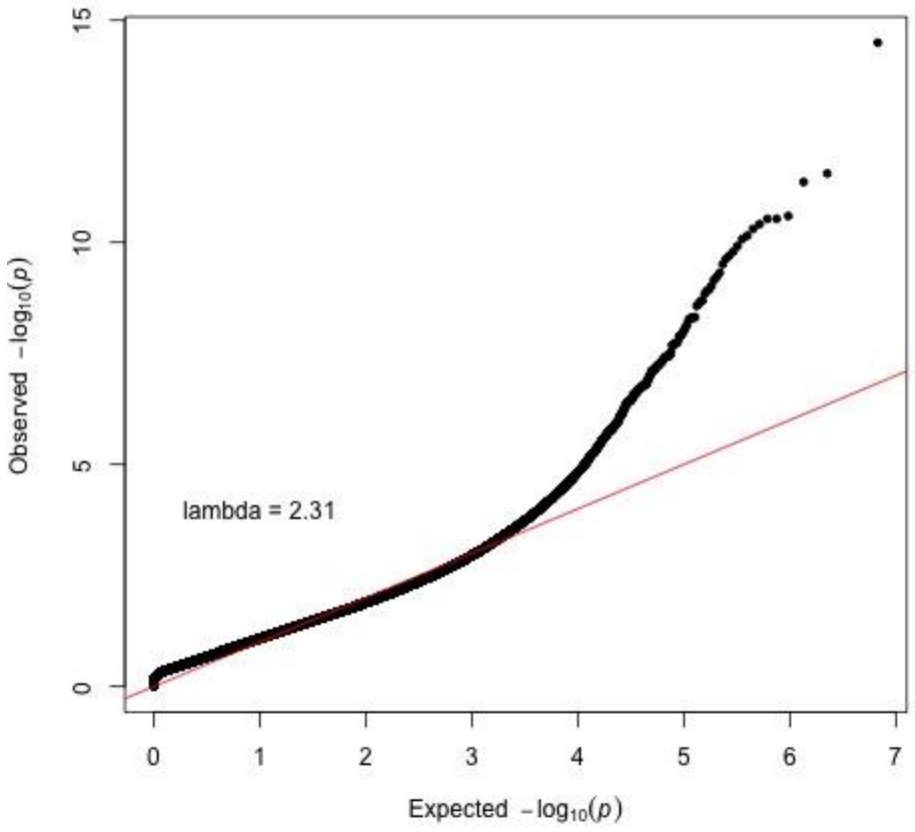

Fig. S27. QQ-plot for GWAS analysis between lobed and unlobed species within clade *c.*

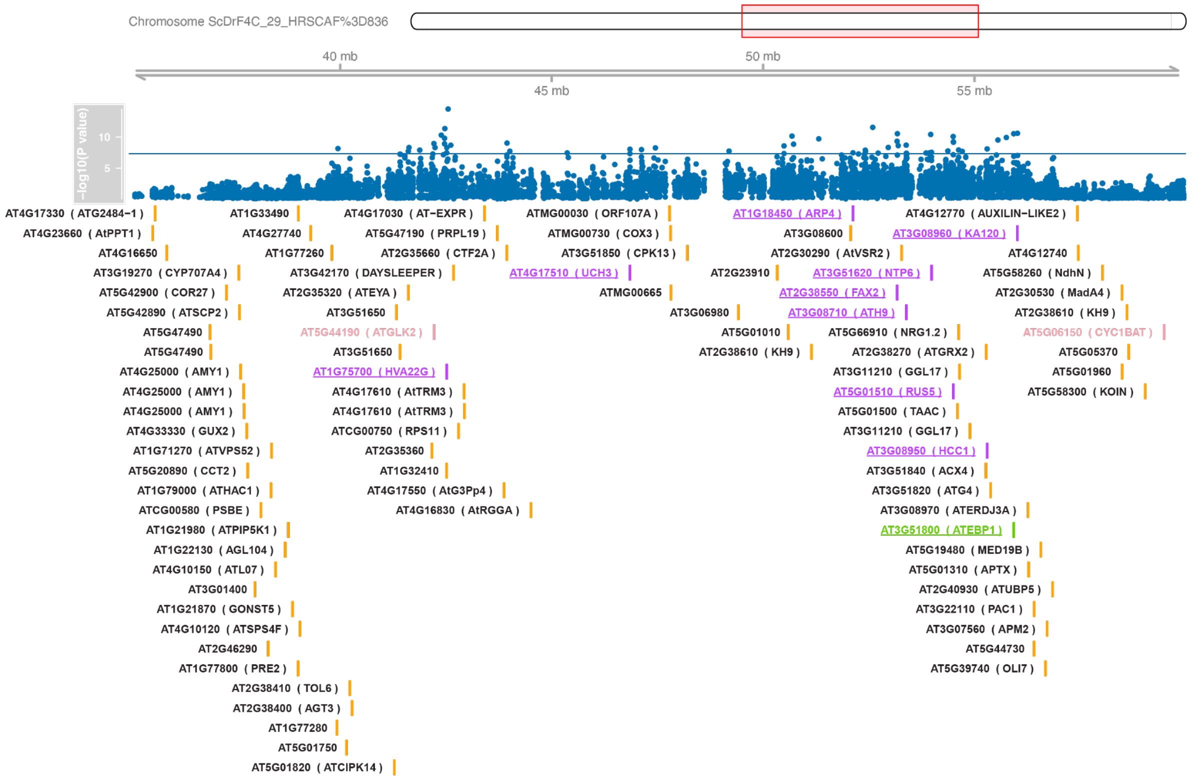

Fig. S28. Zoom on the region on chromosome 29 containing the GWAS peak. Top: p-values for each SNP within the region. Blue line indicates the significant threshold (*p-value* ≥ 5e-08). Bottom: Genes within the region with their *A. thaliana* gene model ID next to them and if available gene symbol in parentheses. Genes associated with leaf development are shown in pink. Genes containing outlier SNPs are underlined and shown in purple (not associated with leaf development) and green (associated with leaf development).

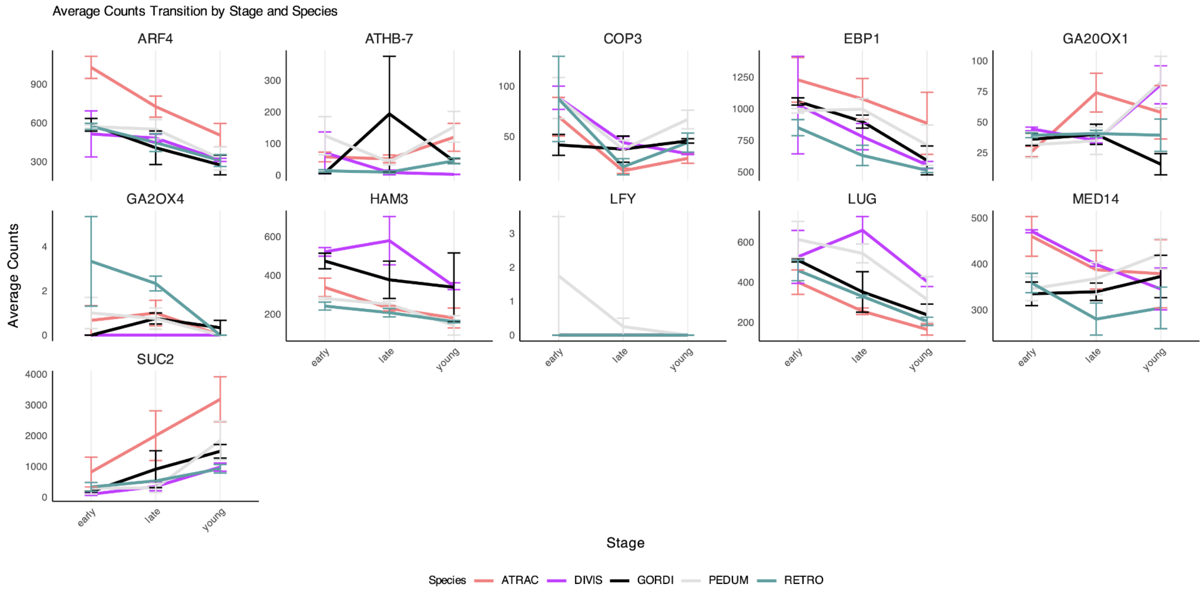

Fig. S29. Expression patterns of selected literature-curated genes across developmental stages in *Scalesia* species. This multi-panel graph depicts the relative expression levels of literature-curated genes (LC genes) putatively under selection across different developmental stages in *Scalesia* species. Expression levels are quantified as transcript counts, and each gene is represented in a separate panel. Points on the graph correspond to the mean expression level for each gene at a given developmental stage, with error bars representing the standard deviation among biological replicates.

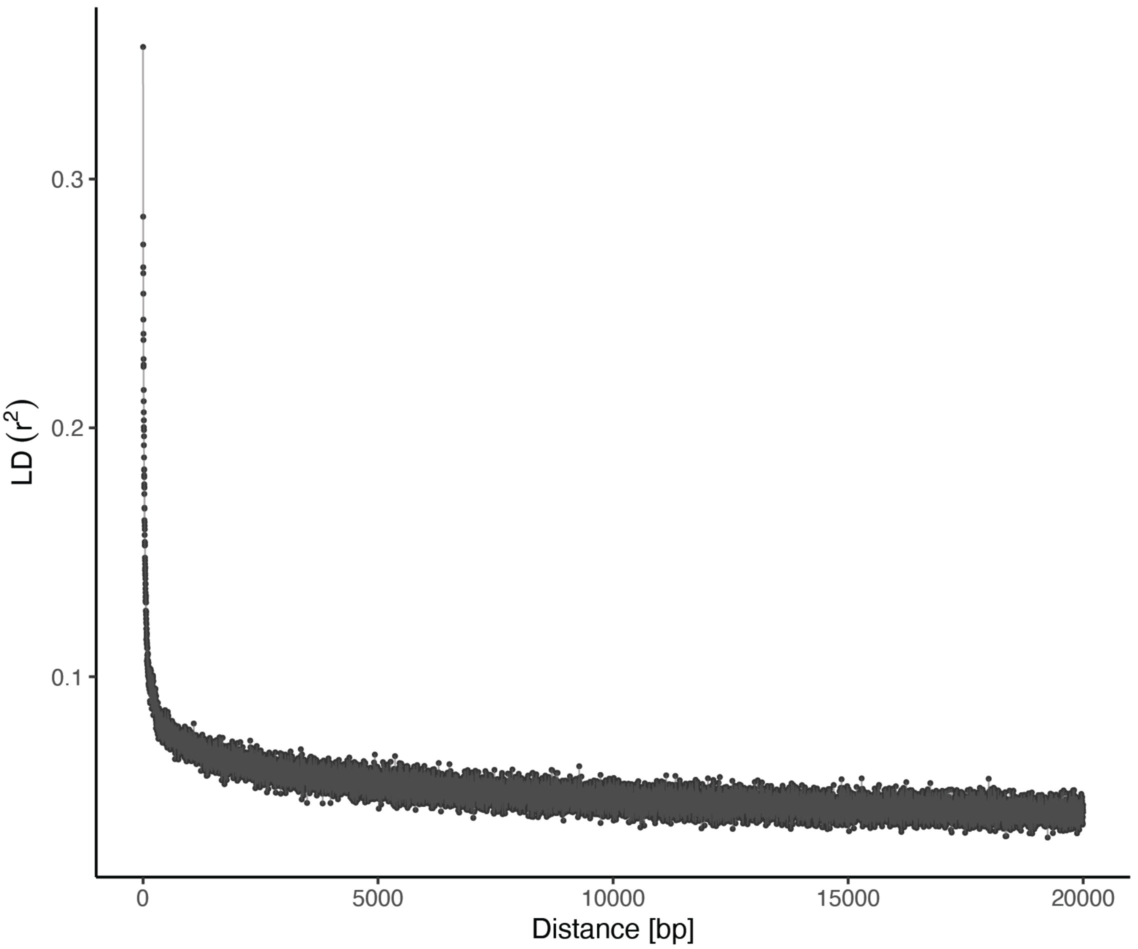

Fig. S30. Linkage disequilibrium (LD) against genomic distance for all *Scalesia* samples combined. The mean value for each distance is plotted. Source data are provided on Dryad (https://doi.org/10.5061/dryad.8gtht76rh).

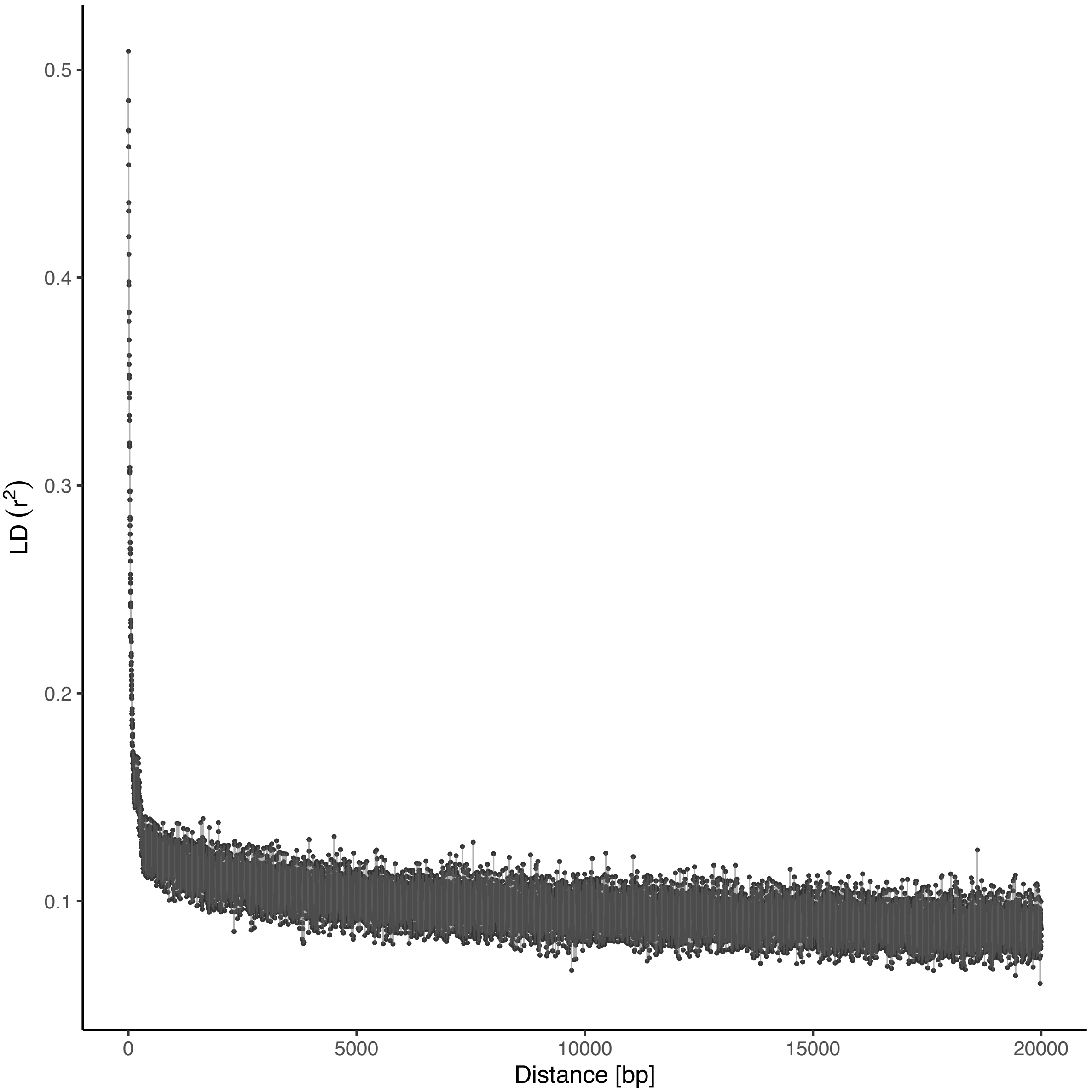

Fig. S31. Linkage disequilibrium (LD) against genomic distance for *Scalesia affinis*. The mean value for each distance is plotted. Source data are provided on Dryad (https://doi.org/10.5061/dryad.8gtht76rh).

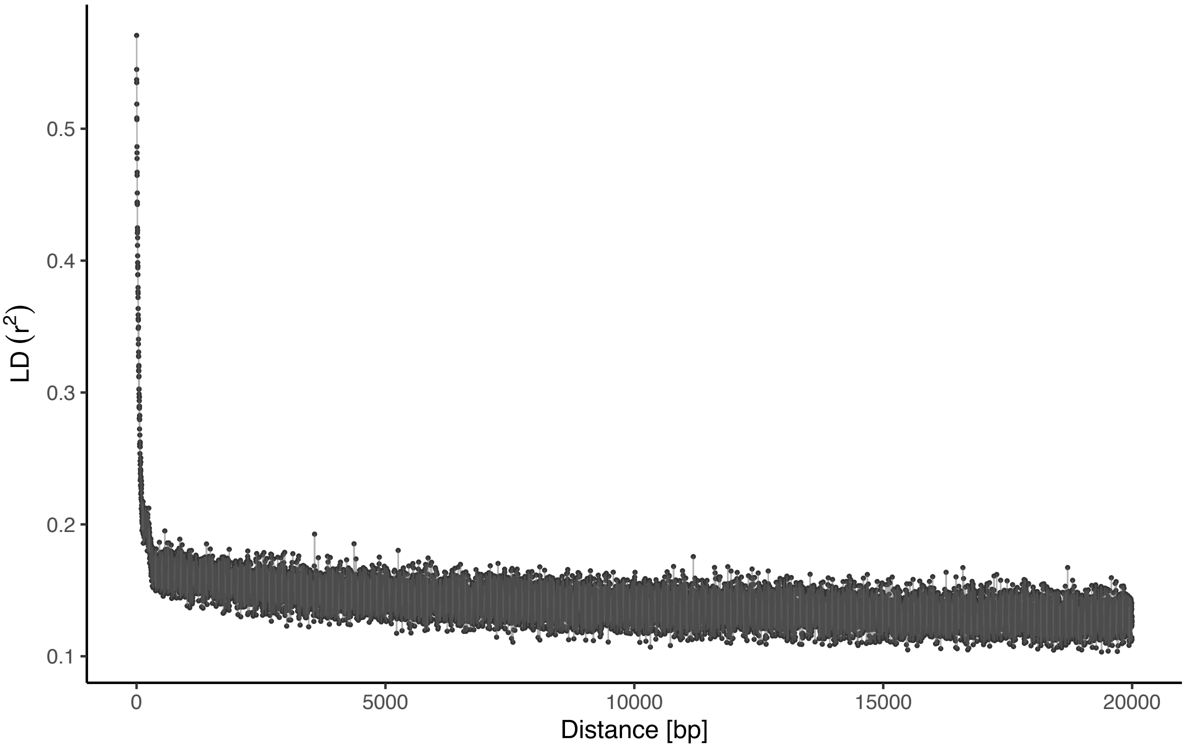

Fig. S32. Linkage disequilibrium (LD) against genomic distance for *Scalesia affinis* (Floreana and Santa Cruz population). The mean value for each distance is plotted. Source data are provided on Dryad (https://doi.org/10.5061/dryad.8gtht76rh).

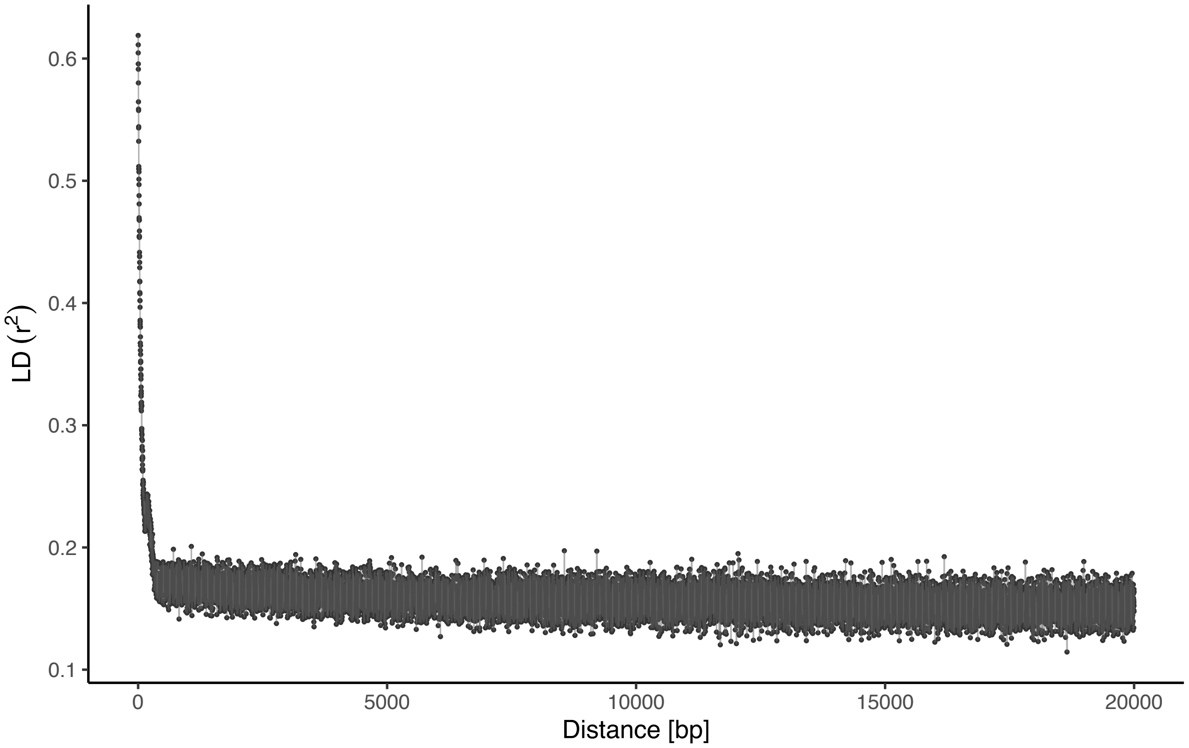

Fig. S33. Linkage disequilibrium (LD) against genomic distance for *Scalesia affinis* (Isabela population). The mean value for each distance is plotted. Source data are provided on Dryad (https://doi.org/10.5061/dryad.8gtht76rh).

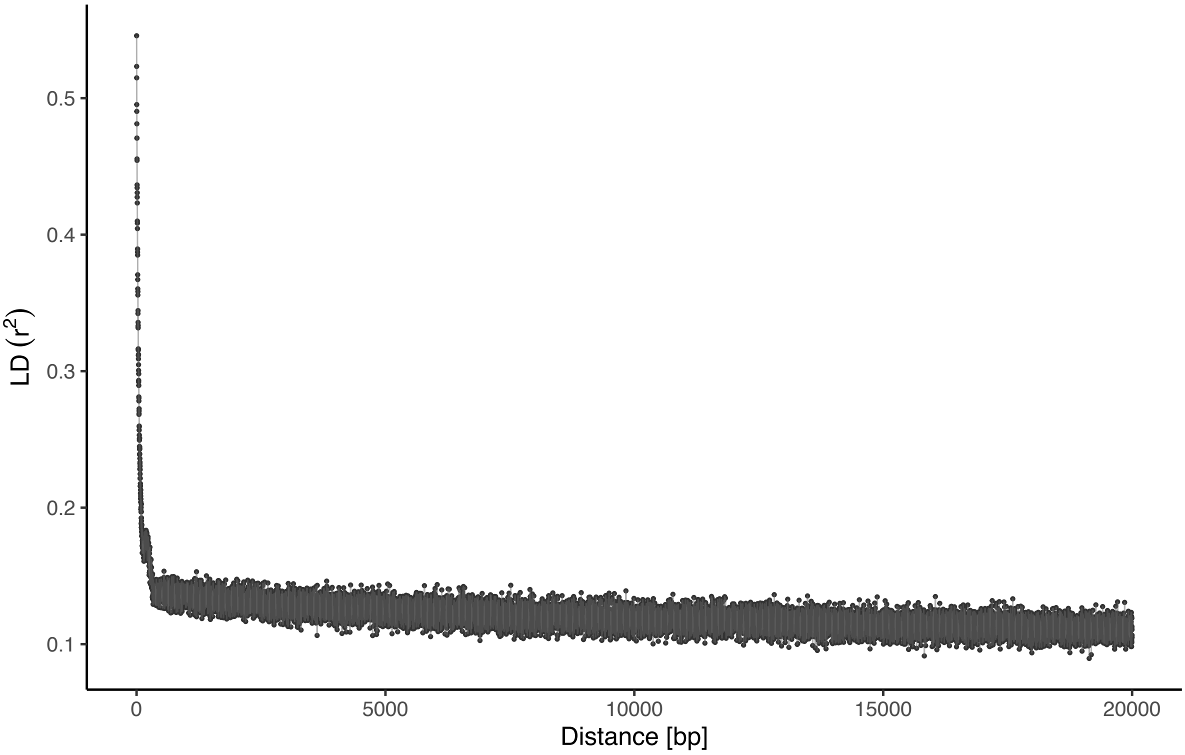

Fig. S34. Linkage disequilibrium (LD) against genomic distance for *Scalesia aspera.* The mean value for each distance is plotted. Source data are provided on Dryad (https://doi.org/10.5061/dryad.8gtht76rh).

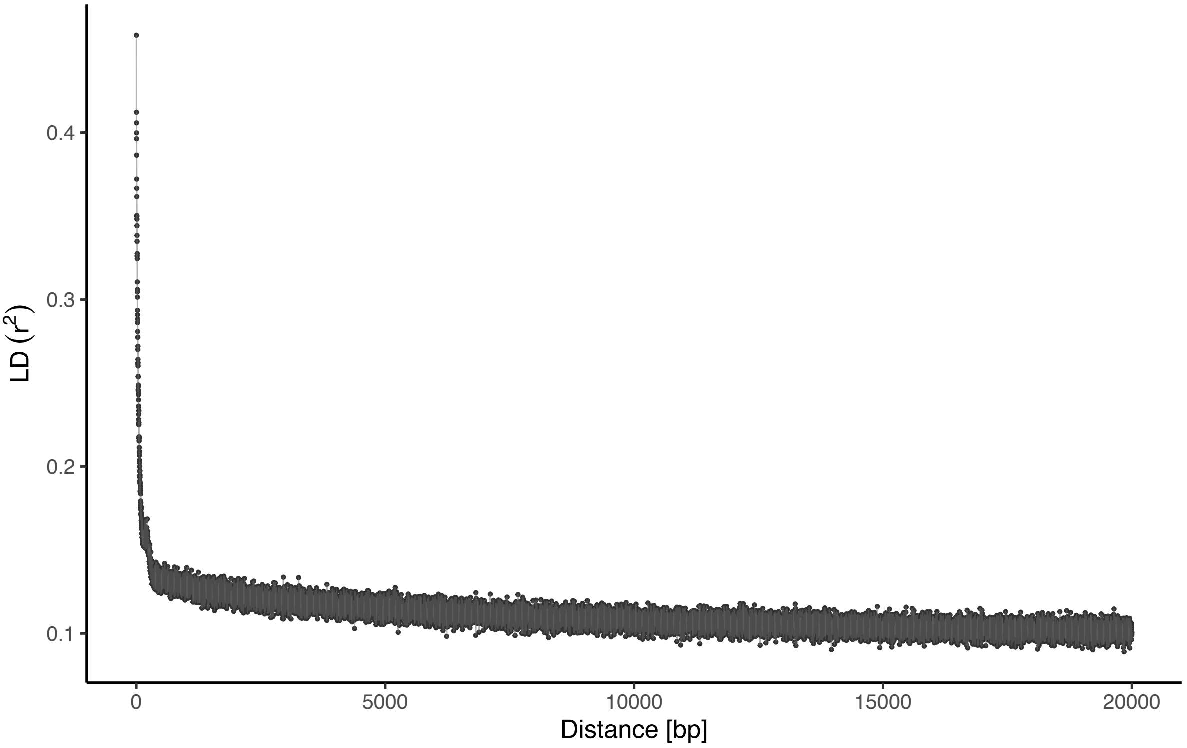

Fig. S35. Linkage disequilibrium (LD) against genomic distance for *Scalesia aspera* x *crockeri.* The mean value for each distance is plotted. Source data are provided on Dryad (https://doi.org/10.5061/dryad.8gtht76rh).

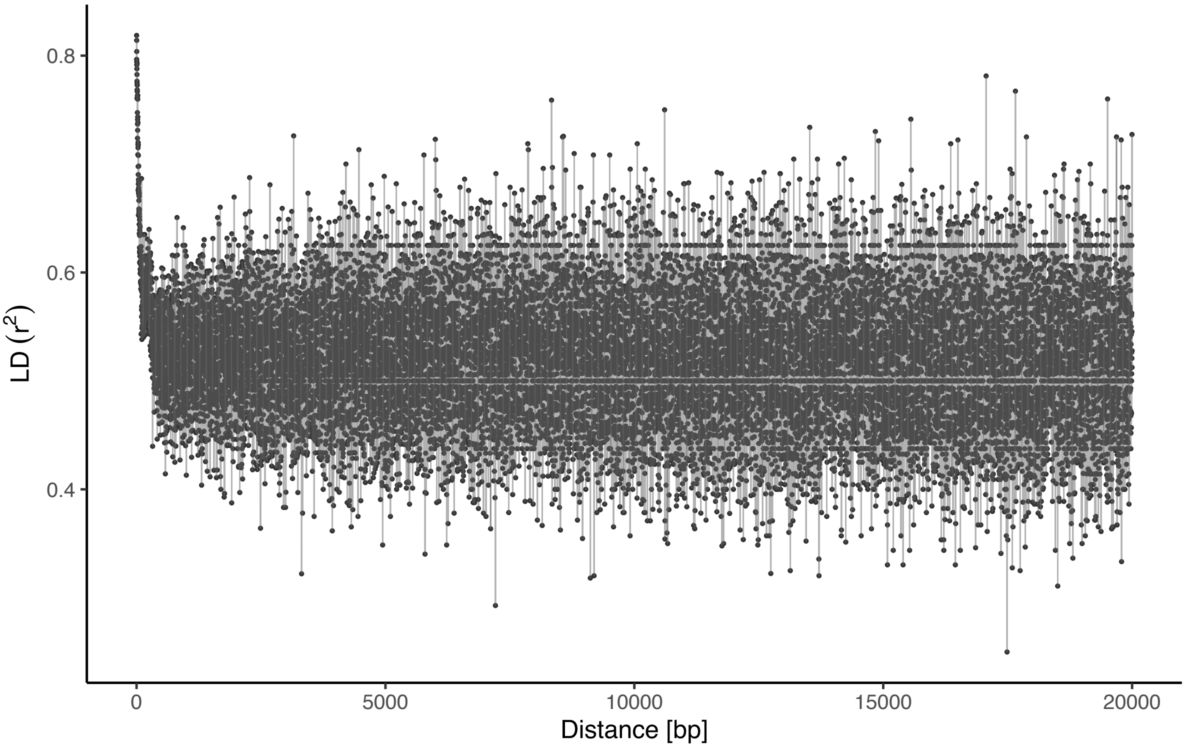

Fig. S36. Linkage disequilibrium (LD) against genomic distance for *Scalesia atractyloides.* The mean value for each distance is plotted. Source data are provided on Dryad (https://doi.org/10.5061/dryad.8gtht76rh).

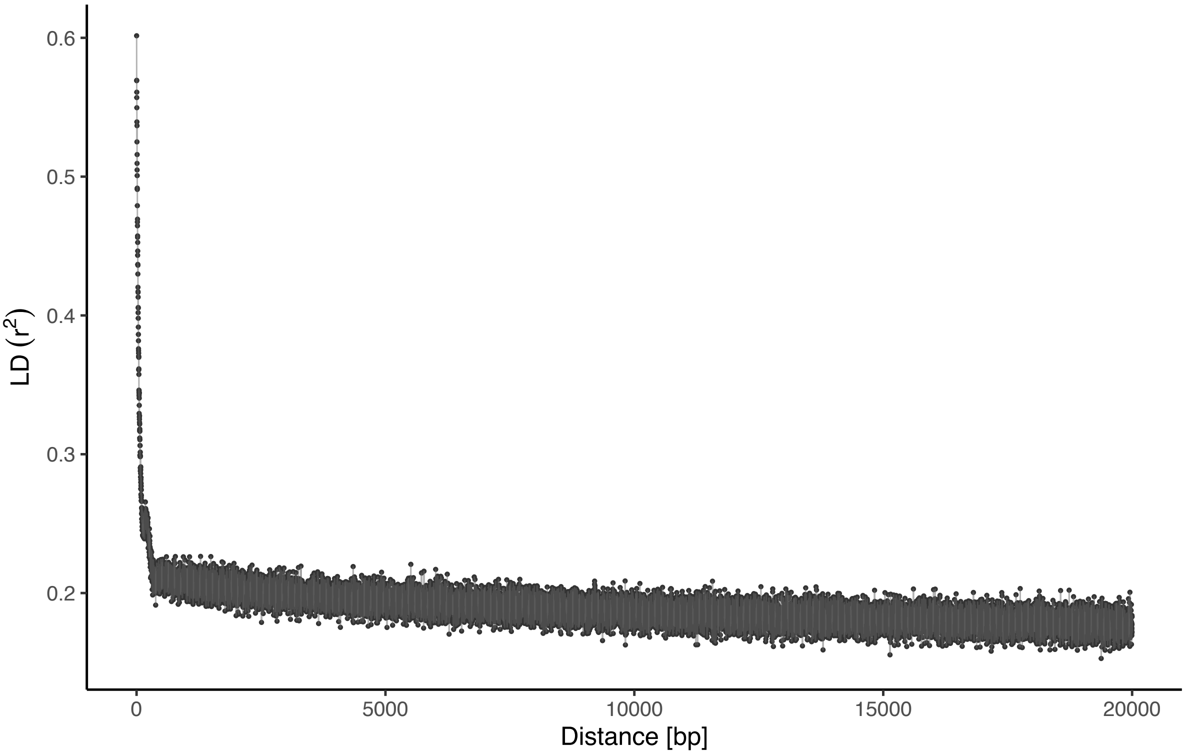

Fig. S37. Linkage disequilibrium (LD) against genomic distance for *Scalesia baurii* ssp. *baurii.* The mean value for each distance is plotted. Source data are provided on Dryad (https://doi.org/10.5061/dryad.8gtht76rh).

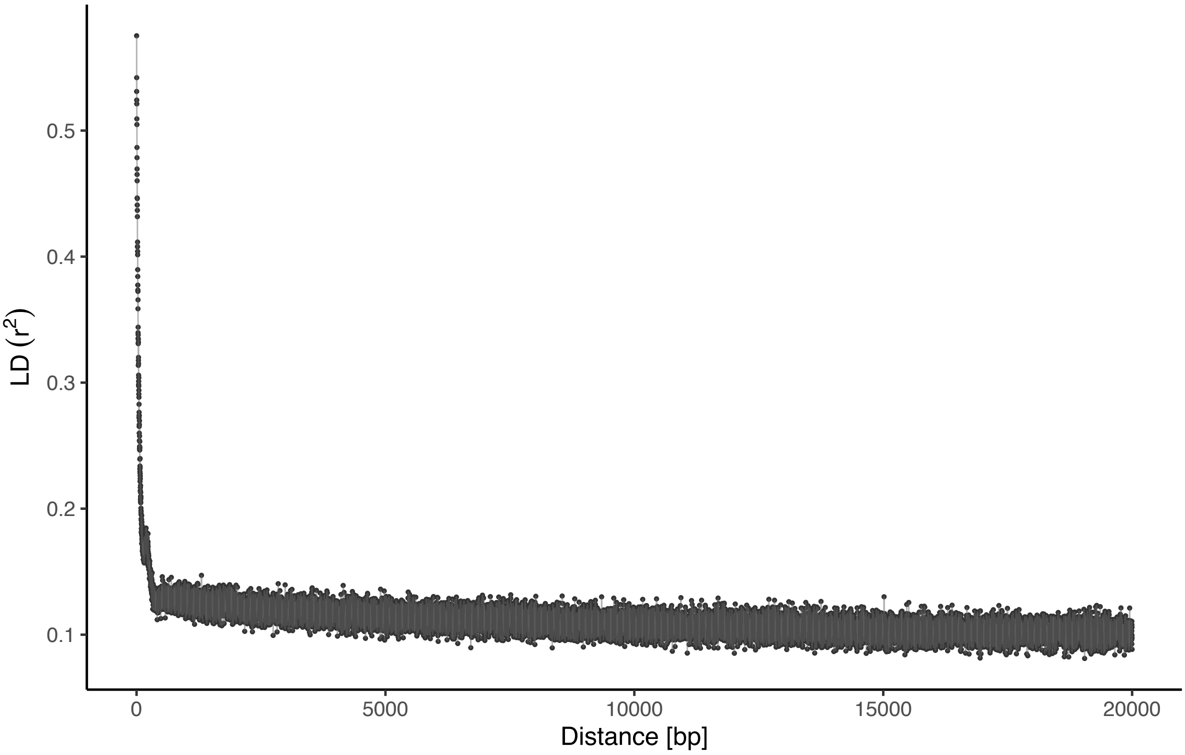

Fig. S38. Linkage disequilibrium (LD) against genomic distance for *Scalesia baurii* ssp. *hopkinsii.* The mean value for each distance is plotted. Source data are provided on Dryad (https://doi.org/10.5061/dryad.8gtht76rh).

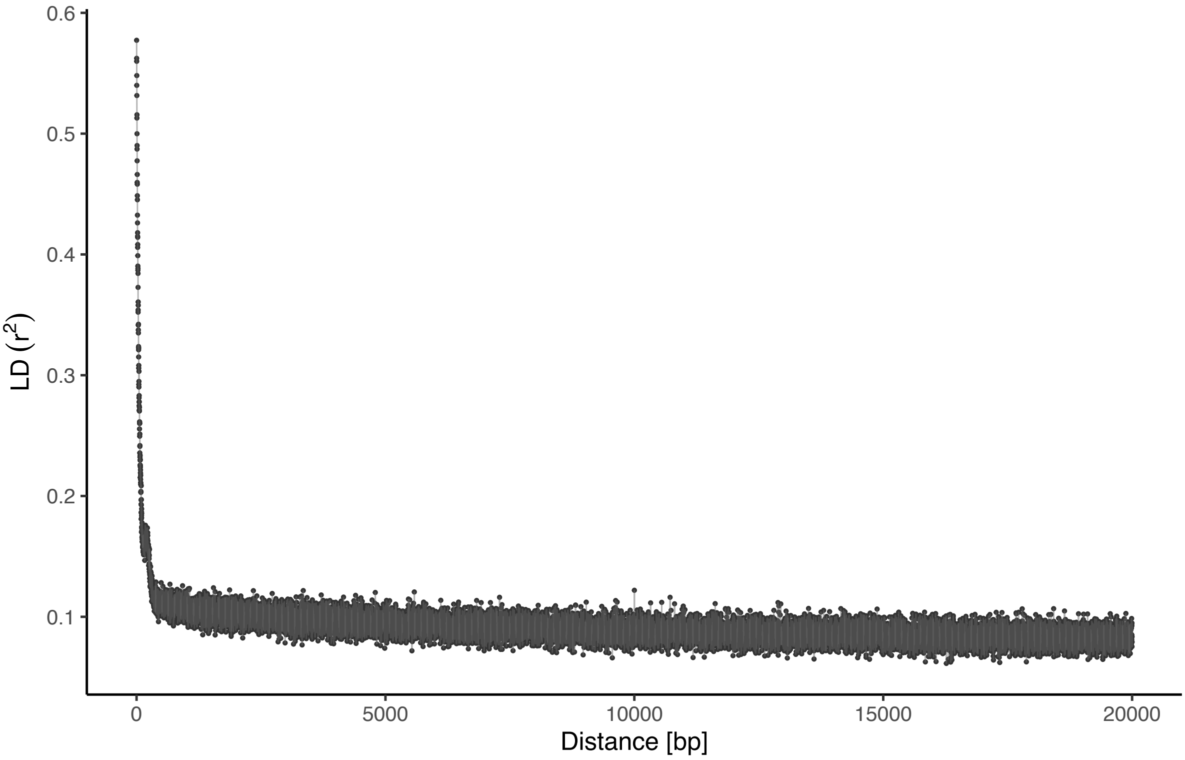

Fig. S39. Linkage disequilibrium (LD) against genomic distance for *Scalesia cordata.* The mean value for each distance is plotted. Source data are provided on Dryad (https://doi.org/10.5061/dryad.8gtht76rh).

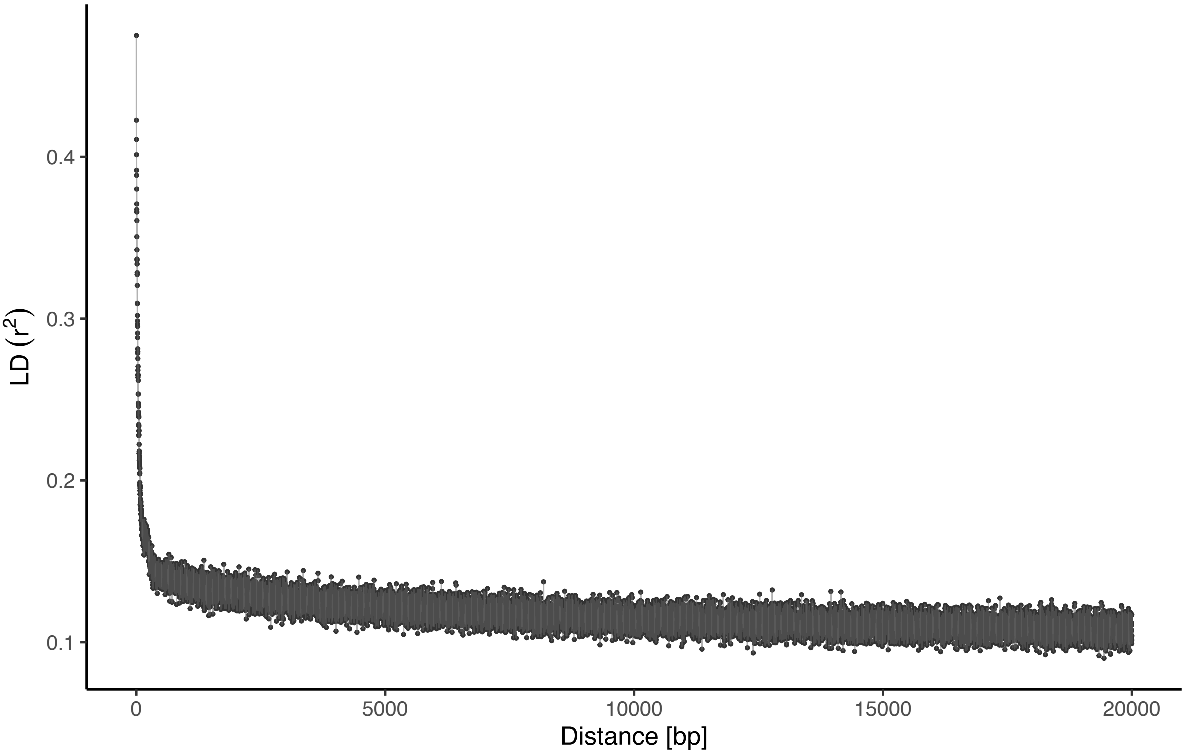

Fig. S40. Linkage disequilibrium (LD) against genomic distance for *Scalesia crockeri.* The mean value for each distance is plotted. Source data are provided on Dryad (https://doi.org/10.5061/dryad.8gtht76rh).

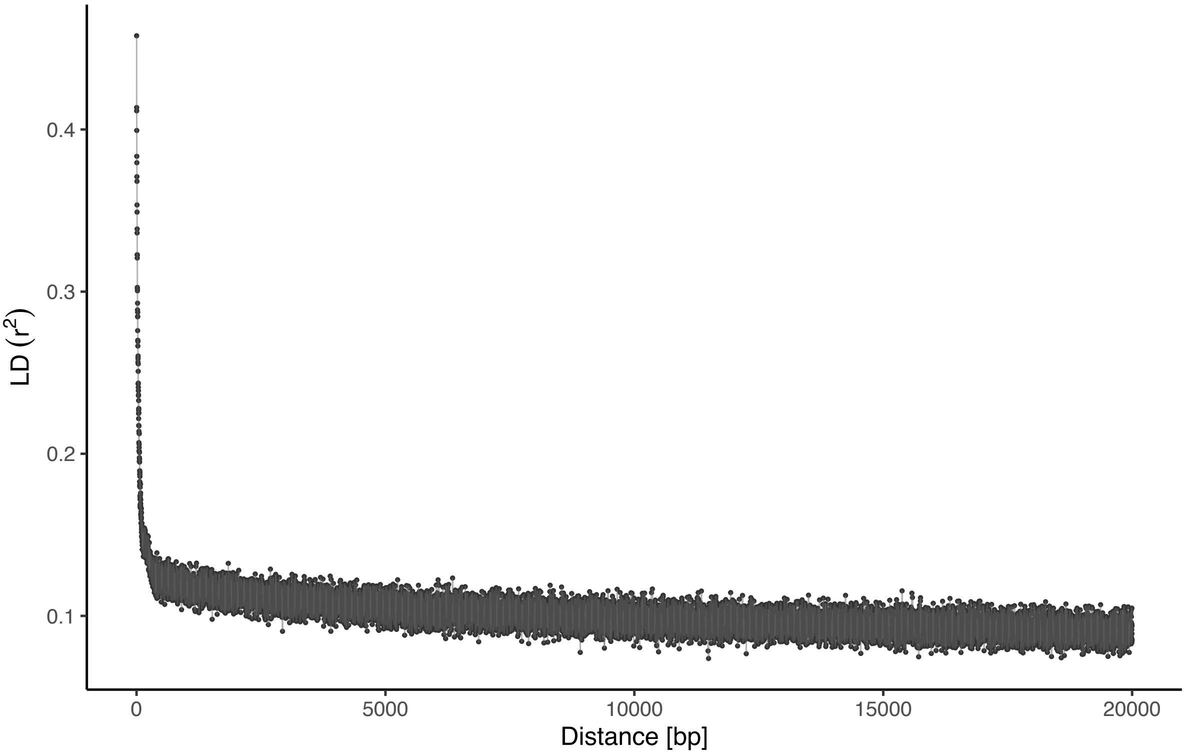

Fig. S41. Linkage disequilibrium (LD) against genomic distance for *Scalesia divisa.* The mean value for each distance is plotted. Source data are provided on Dryad (https://doi.org/10.5061/dryad.8gtht76rh).

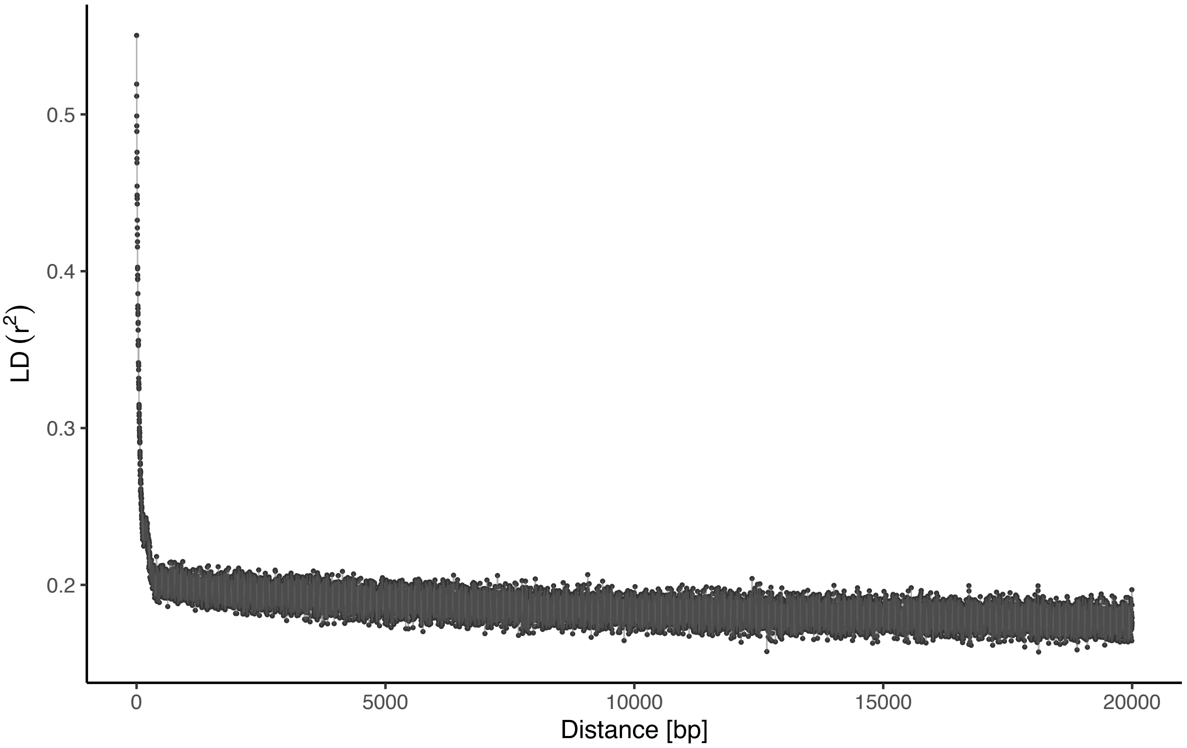

Fig. S42. Linkage disequilibrium (LD) against genomic distance for *Scalesia divisa* x *incisa.* The mean value for each distance is plotted. Source data are provided on Dryad (https://doi.org/10.5061/dryad.8gtht76rh).

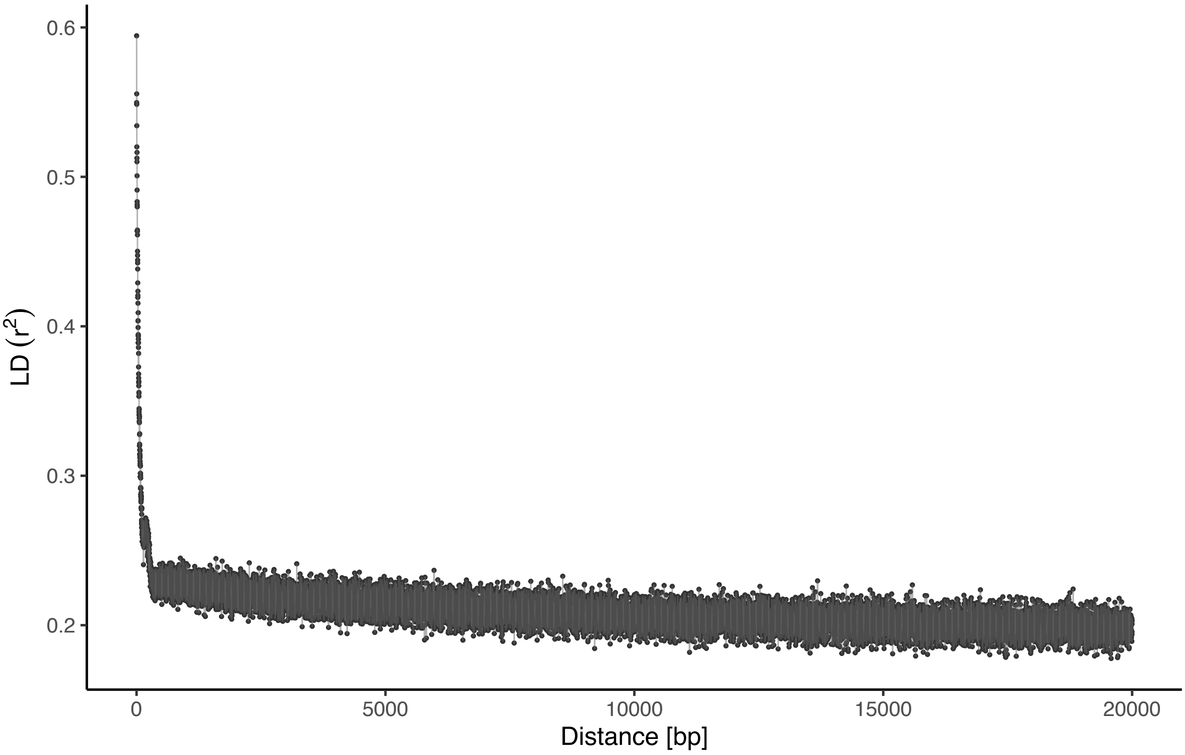

Fig. S43. Linkage disequilibrium (LD) against genomic distance for *Scalesia gordilloi.* The mean value for each distance is plotted. Source data are provided on Dryad (https://doi.org/10.5061/dryad.8gtht76rh).

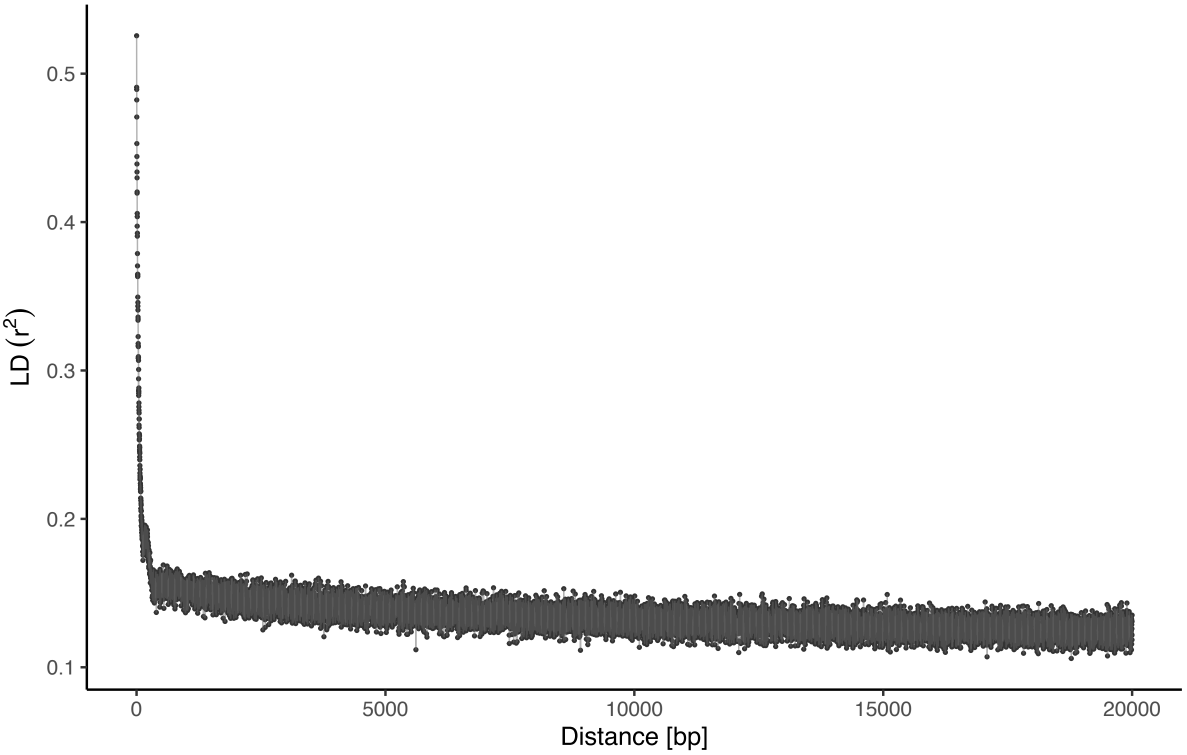

Fig. S44. Linkage disequilibrium (LD) against genomic distance for *Scalesia helleri.* The mean value for each distance is plotted. Source data are provided on Dryad (https://doi.org/10.5061/dryad.8gtht76rh).

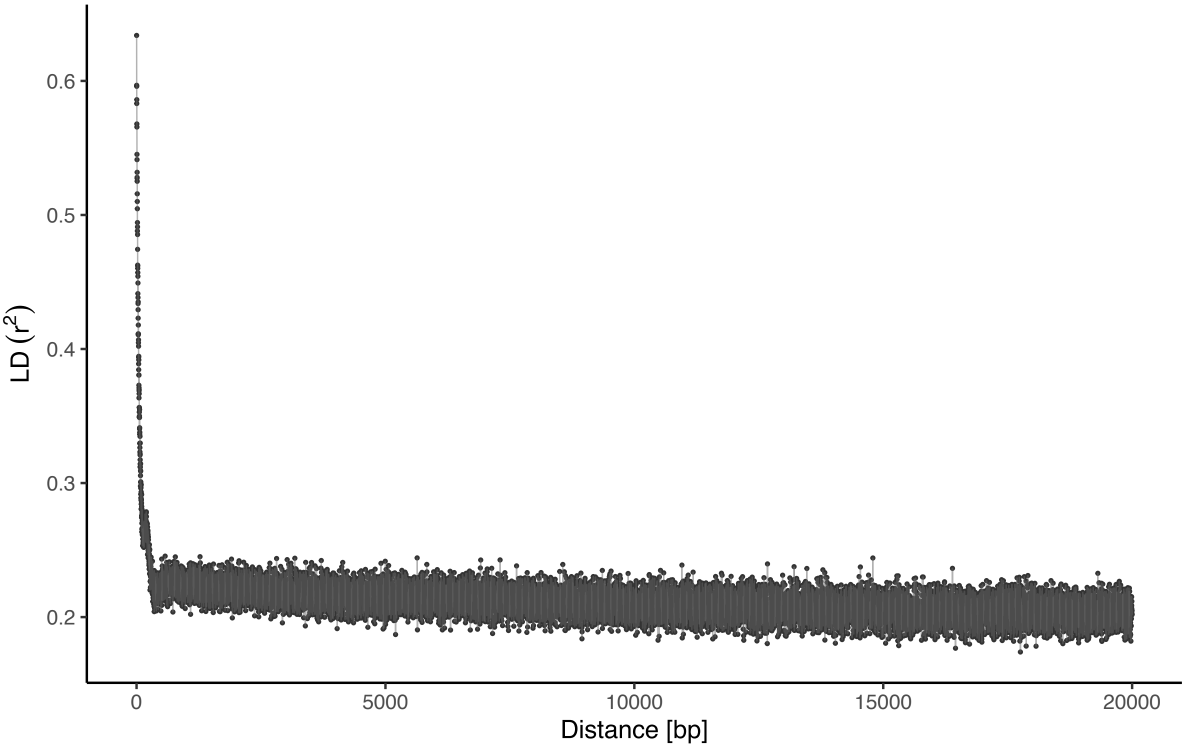

Fig. S45. Linkage disequilibrium (LD) against genomic distance for *Scalesia helleri* (Santa Cruz population). The mean value for each distance is plotted. Source data are provided on Dryad (https://doi.org/10.5061/dryad.8gtht76rh).

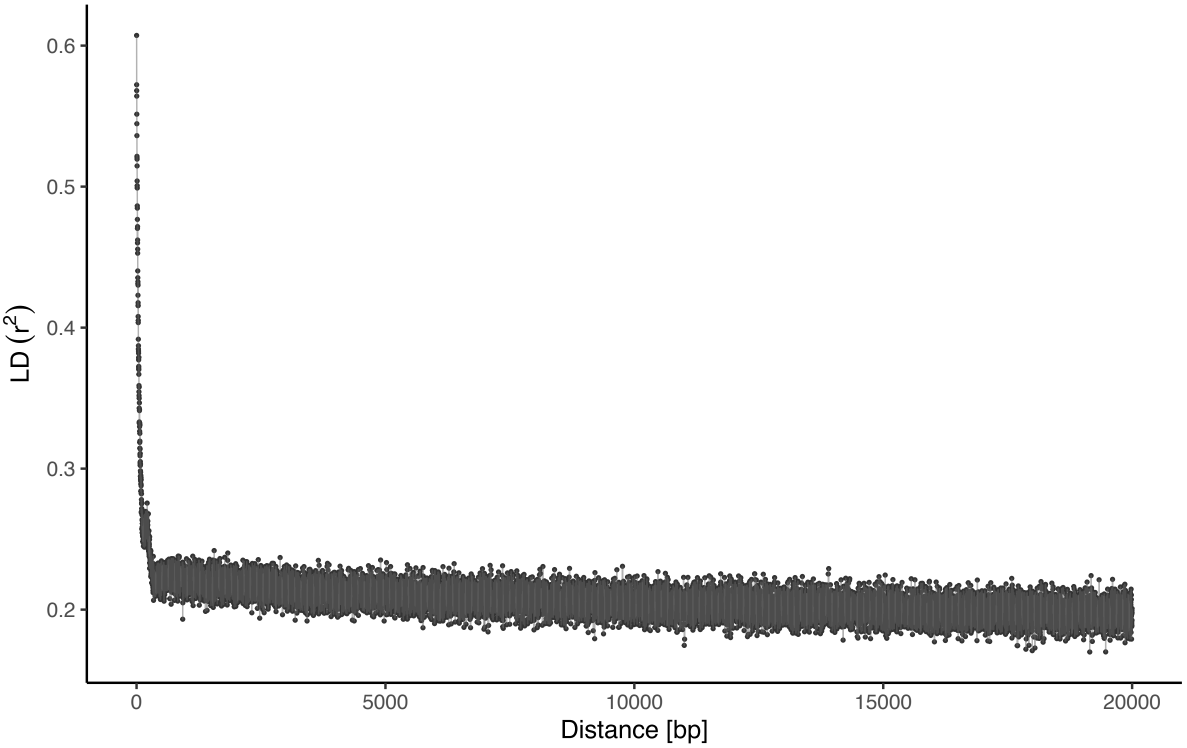

Fig. S46. Linkage disequilibrium (LD) against genomic distance for *Scalesia helleri* (Santa Fe population). The mean value for each distance is plotted. Source data are provided on Dryad (https://doi.org/10.5061/dryad.8gtht76rh).

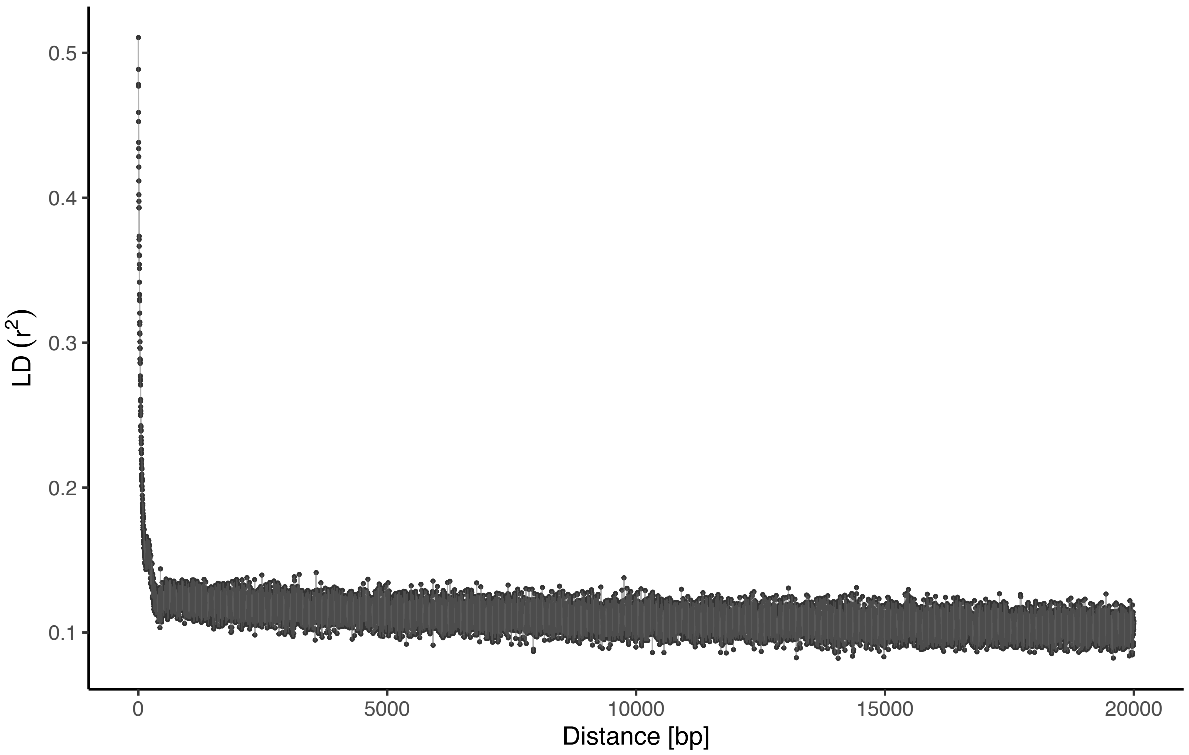

Fig. S47. Linkage disequilibrium (LD) against genomic distance for *Scalesia incisa.* The mean value for each distance is plotted. Source data are provided on Dryad (https://doi.org/10.5061/dryad.8gtht76rh).

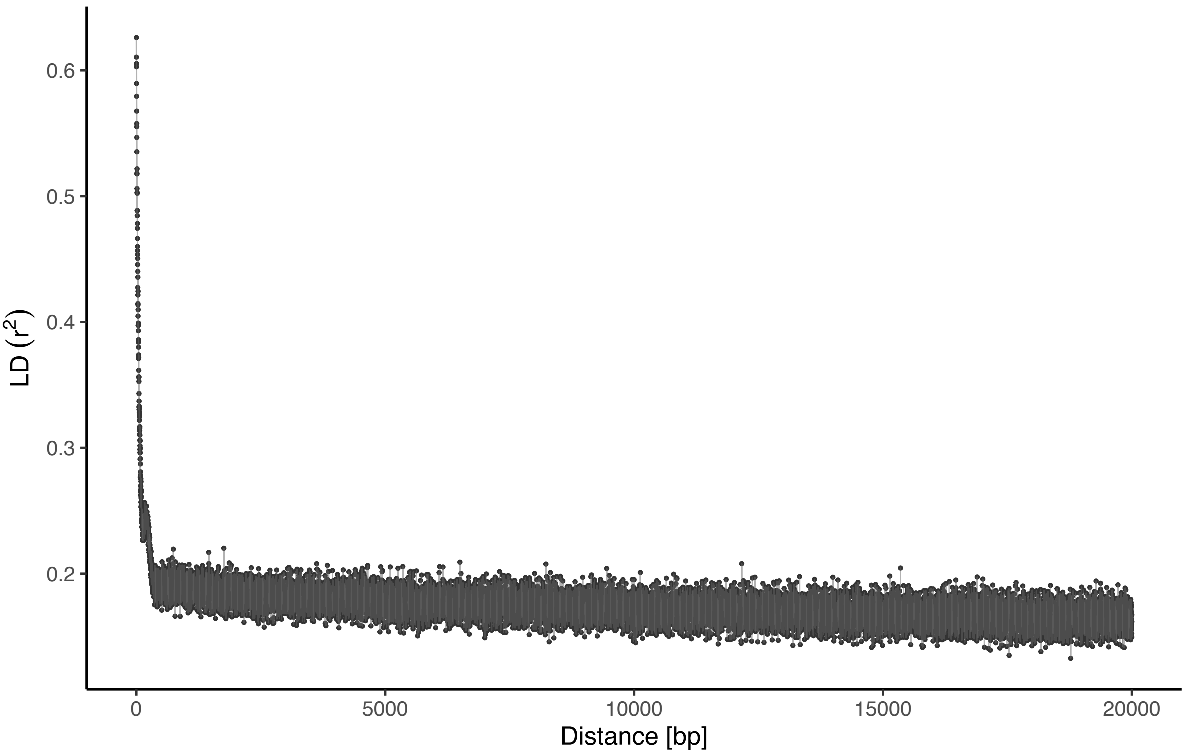

Fig. S48. Linkage disequilibrium (LD) against genomic distance for *Scalesia microcephala.* The mean value for each distance is plotted. Source data are provided on Dryad (https://doi.org/10.5061/dryad.8gtht76rh).

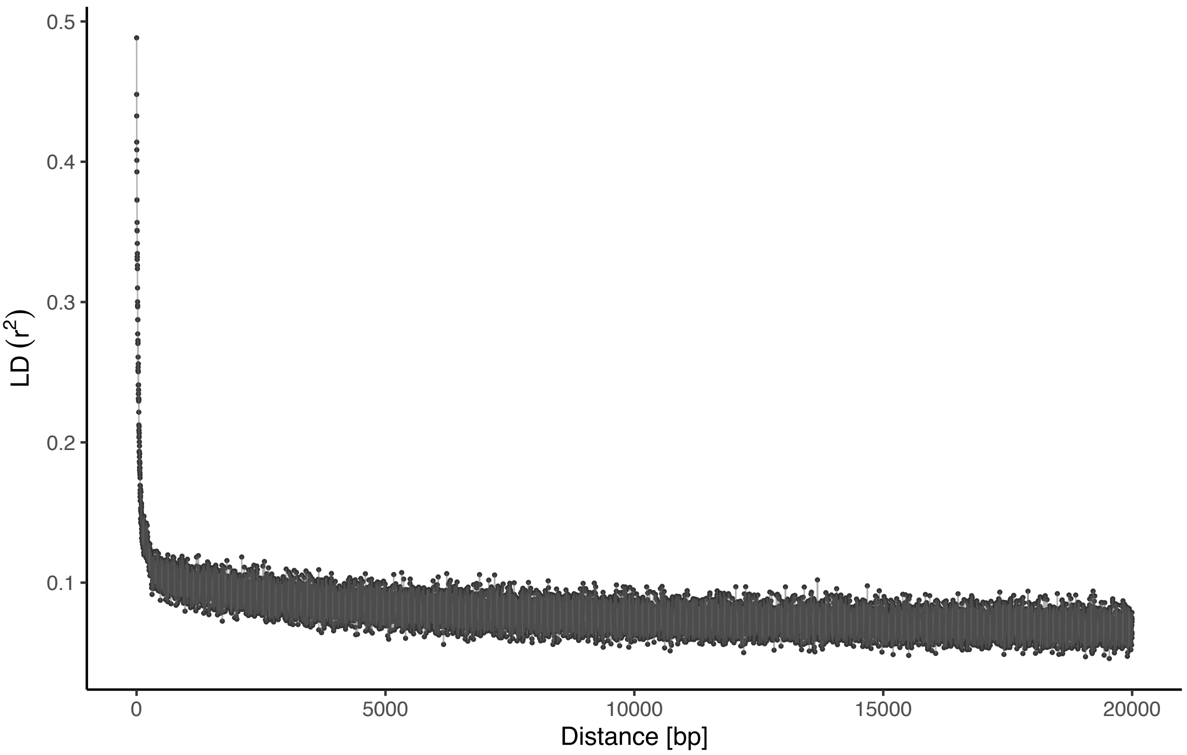

Fig. S49. Linkage disequilibrium (LD) against genomic distance for *Scalesia pedunculata.* The mean value for each distance is plotted. Source data are provided on Dryad (https://doi.org/10.5061/dryad.8gtht76rh).

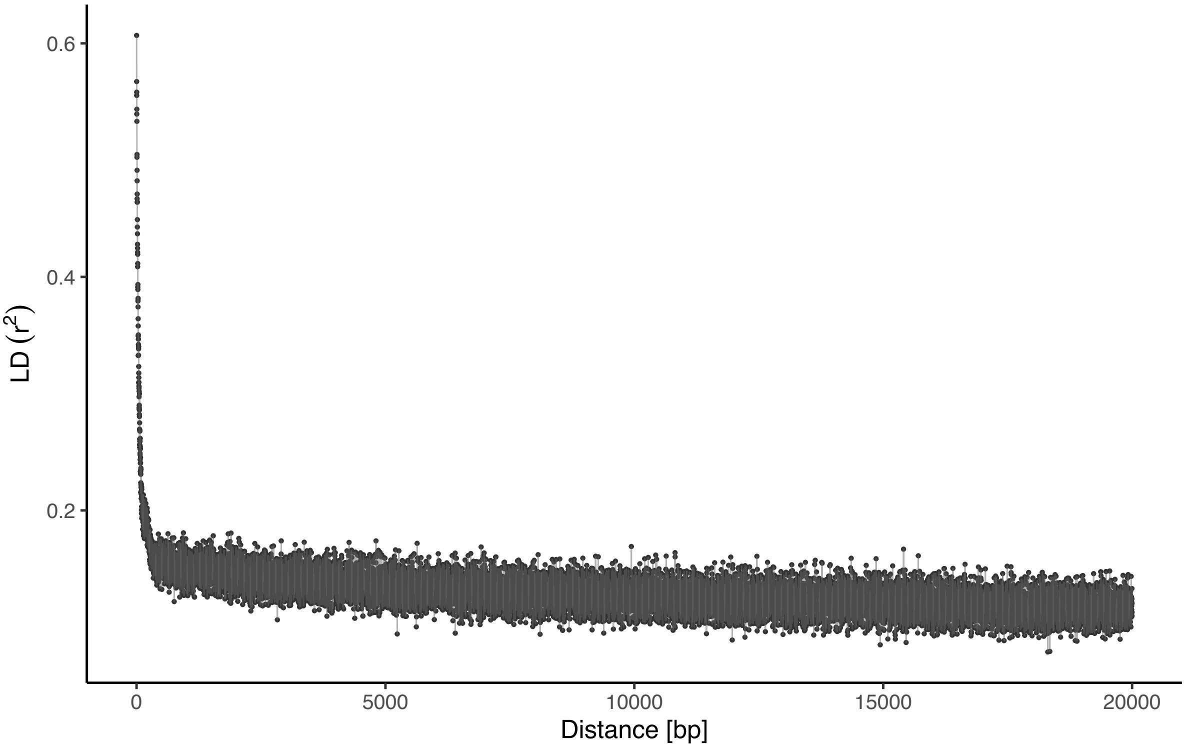

Fig. S50. Linkage disequilibrium (LD) against genomic distance for *Scalesia pedunculata* 1. The mean value for each distance is plotted. Source data are provided on Dryad (https://doi.org/10.5061/dryad.8gtht76rh).

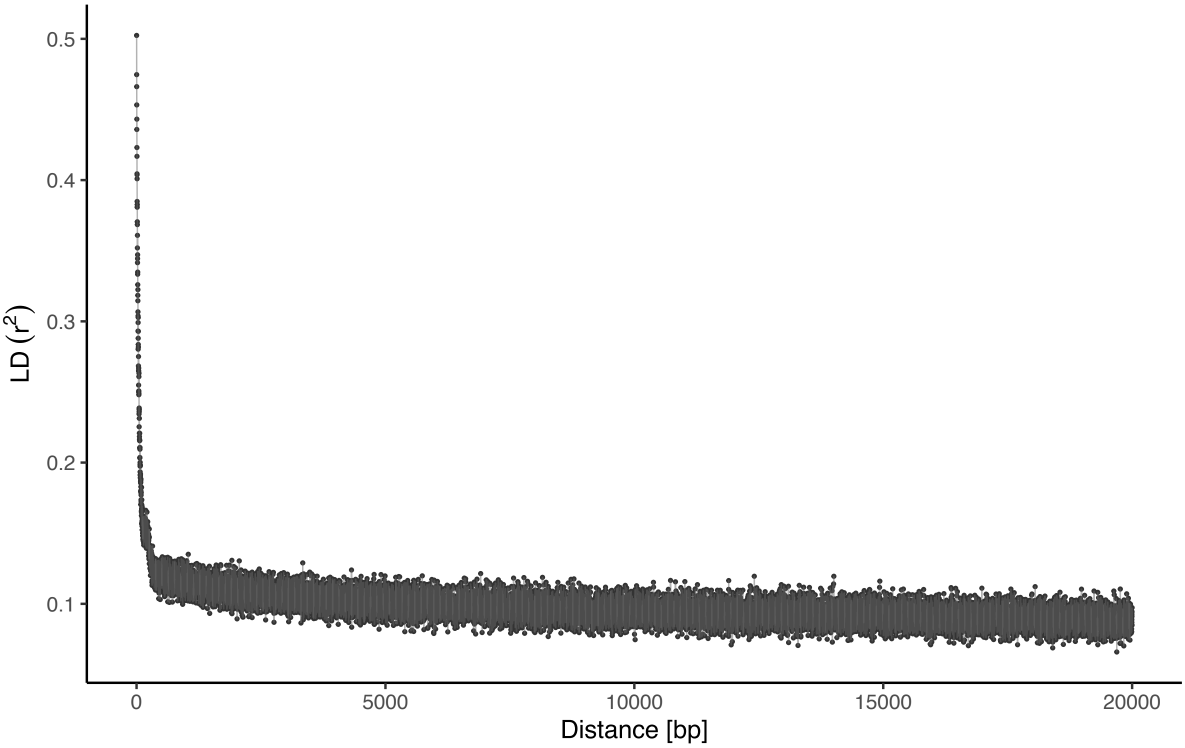

Fig. S51. Linkage disequilibrium (LD) against genomic distance for *Scalesia pedunculata* 2. The mean value for each distance is plotted. Source data are provided on Dryad (https://doi.org/10.5061/dryad.8gtht76rh).

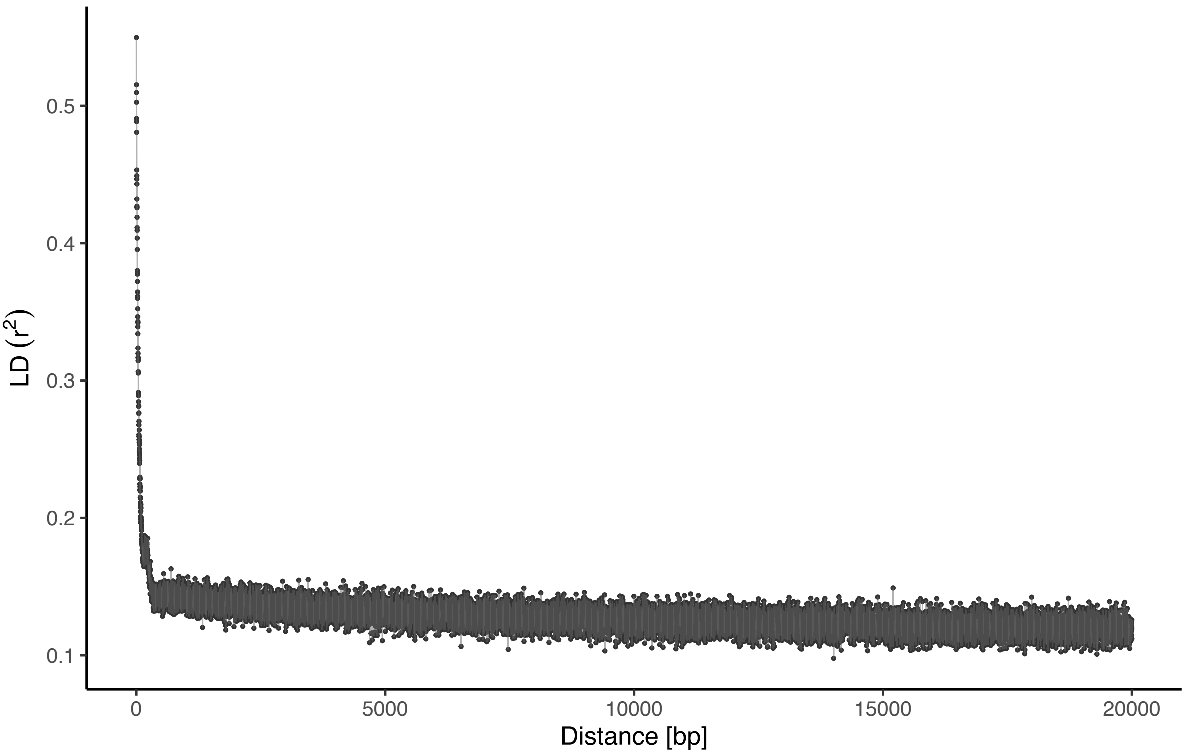

Fig. S52. Linkage disequilibrium (LD) against genomic distance for *Scalesia retroflexa.* The mean value for each distance is plotted. Source data are provided on Dryad (https://doi.org/10.5061/dryad.8gtht76rh).

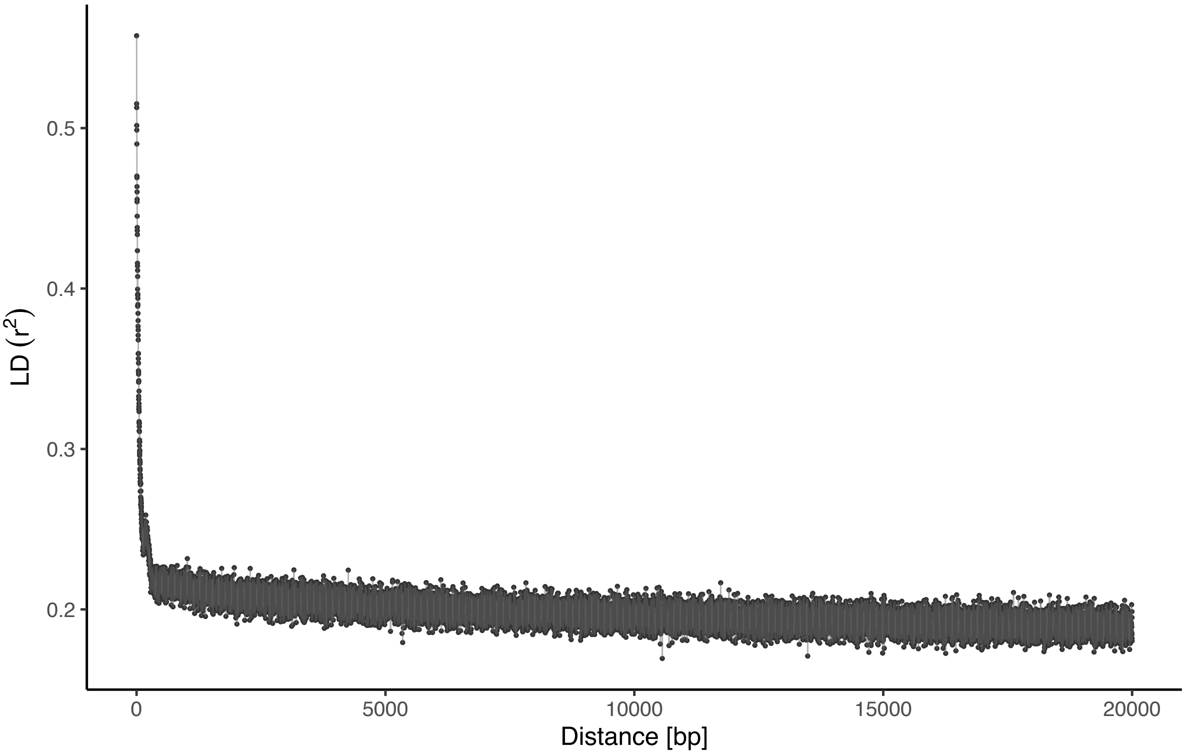

Fig. S53. Linkage disequilibrium (LD) against genomic distance for *Scalesia* cfr. *retroflexa.* The mean value for each distance is plotted. Source data are provided on Dryad (https://doi.org/10.5061/dryad.8gtht76rh).

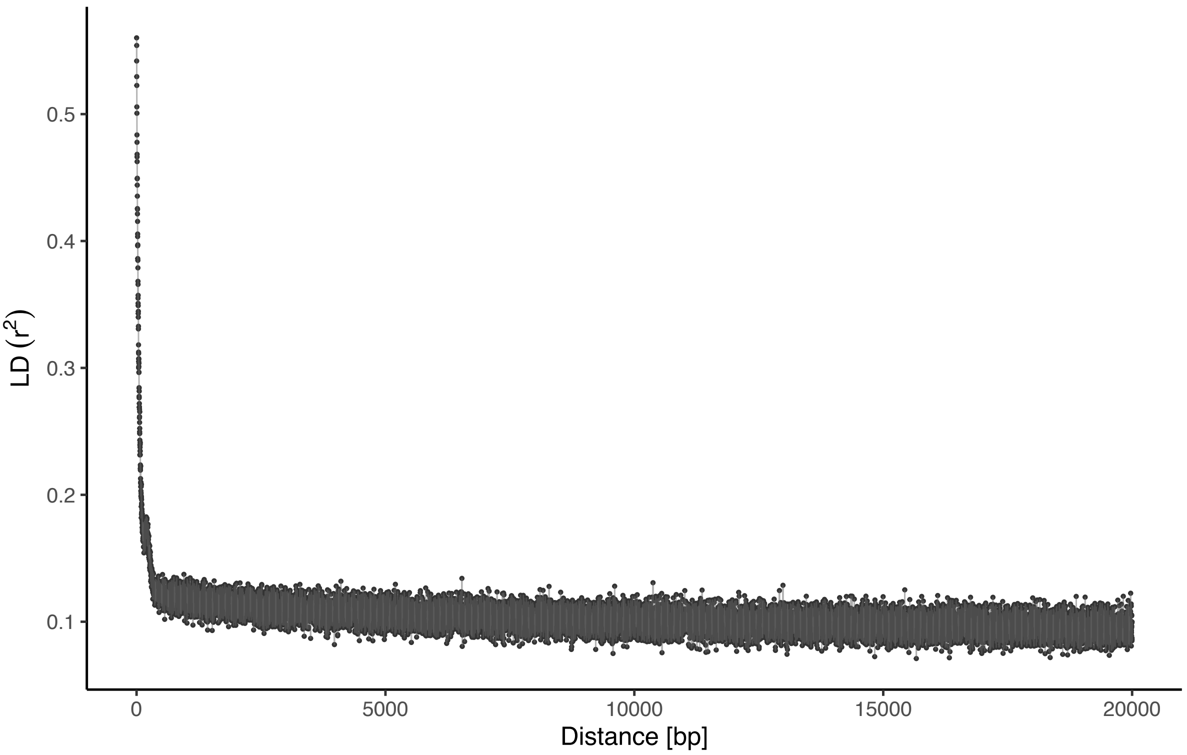

Fig. S54. Linkage disequilibrium (LD) against genomic distance for *Scalesia stewartii.* The mean value for each distance is plotted. Source data are provided on Dryad (https://doi.org/10.5061/dryad.8gtht76rh).

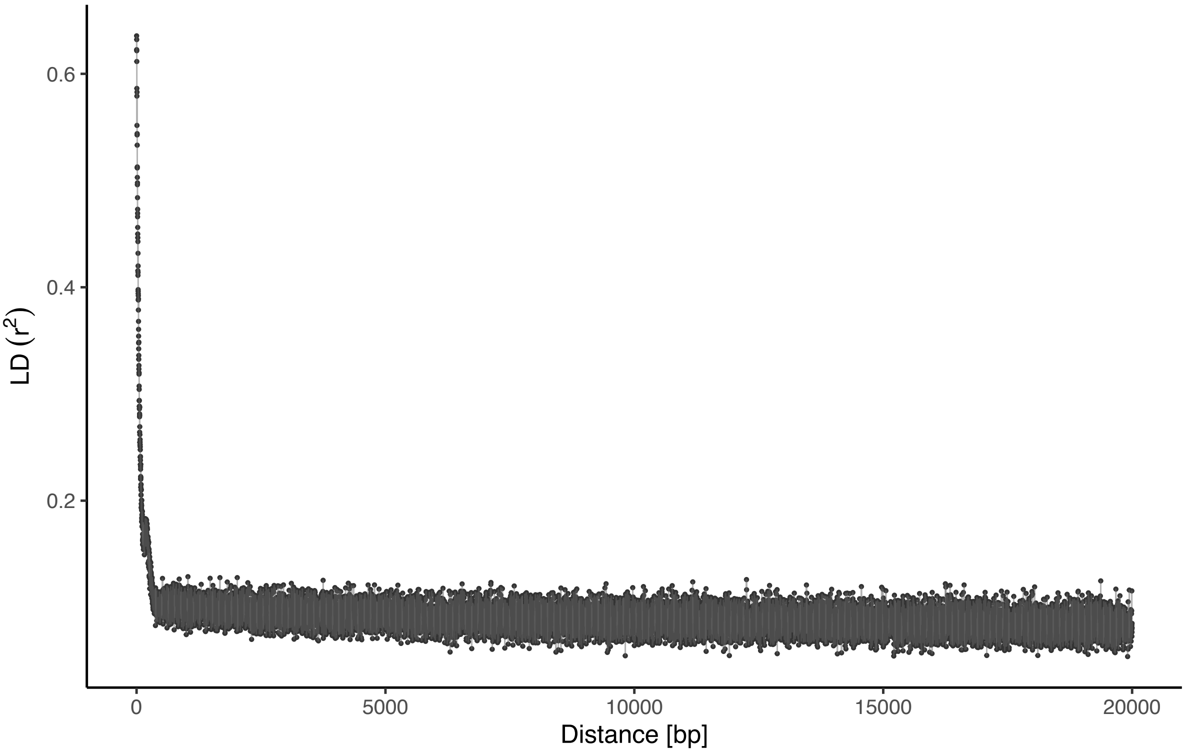

Fig. S55. Linkage disequilibrium (LD) against genomic distance for *Scalesia villosa.* The mean value for each distance is plotted. Source data are provided on Dryad (https://doi.org/10.5061/dryad.8gtht76rh).

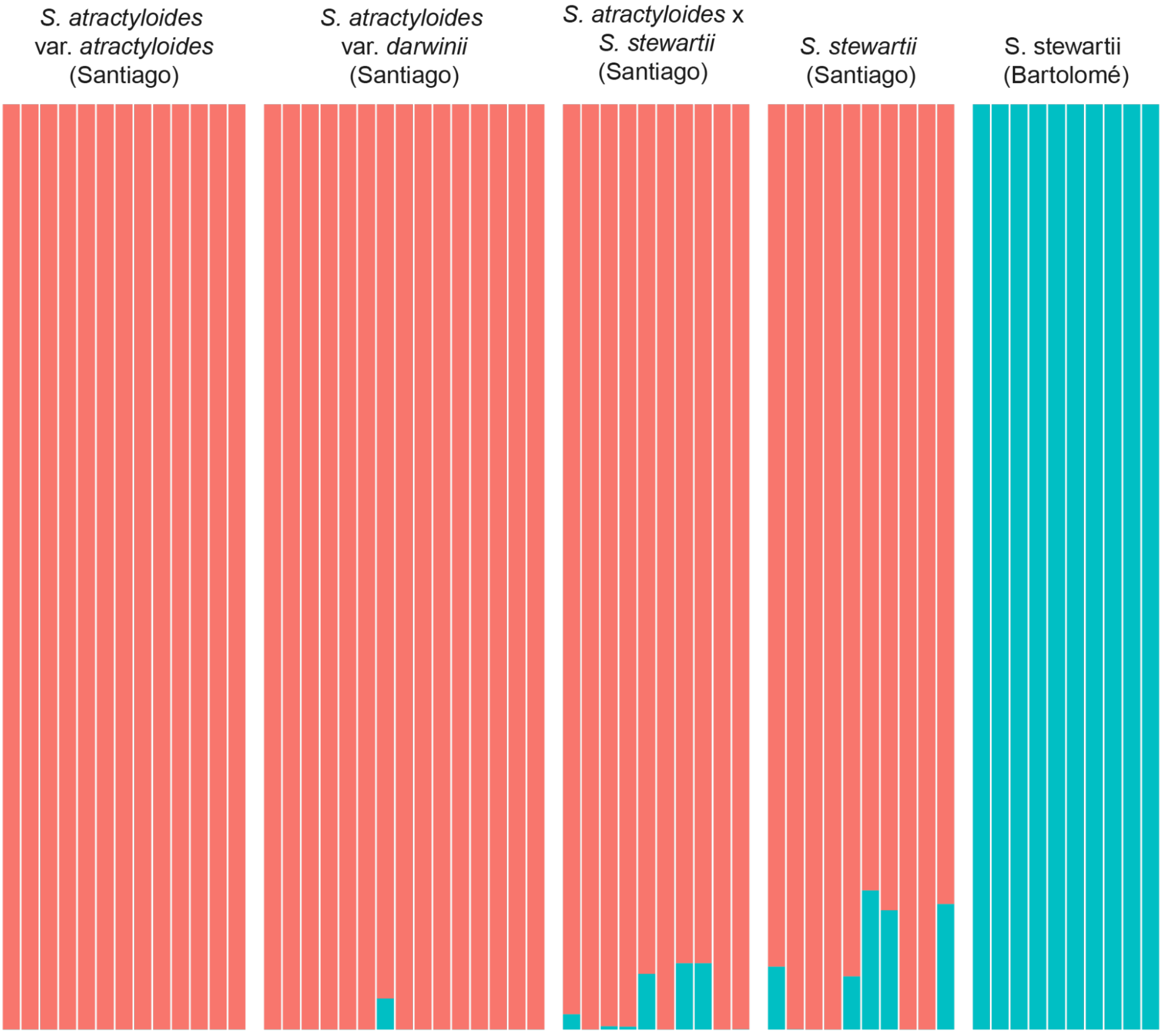

Figure S56. Admixture analysis for *K*=2 of *Scalesia stewartii, Scalesia atractyloides*, and potential *S. atractyloides* x *S. stewartii* hybrids. Source data are provided on Dryad (https://doi.org/10.5061/dryad.8gtht76rh).

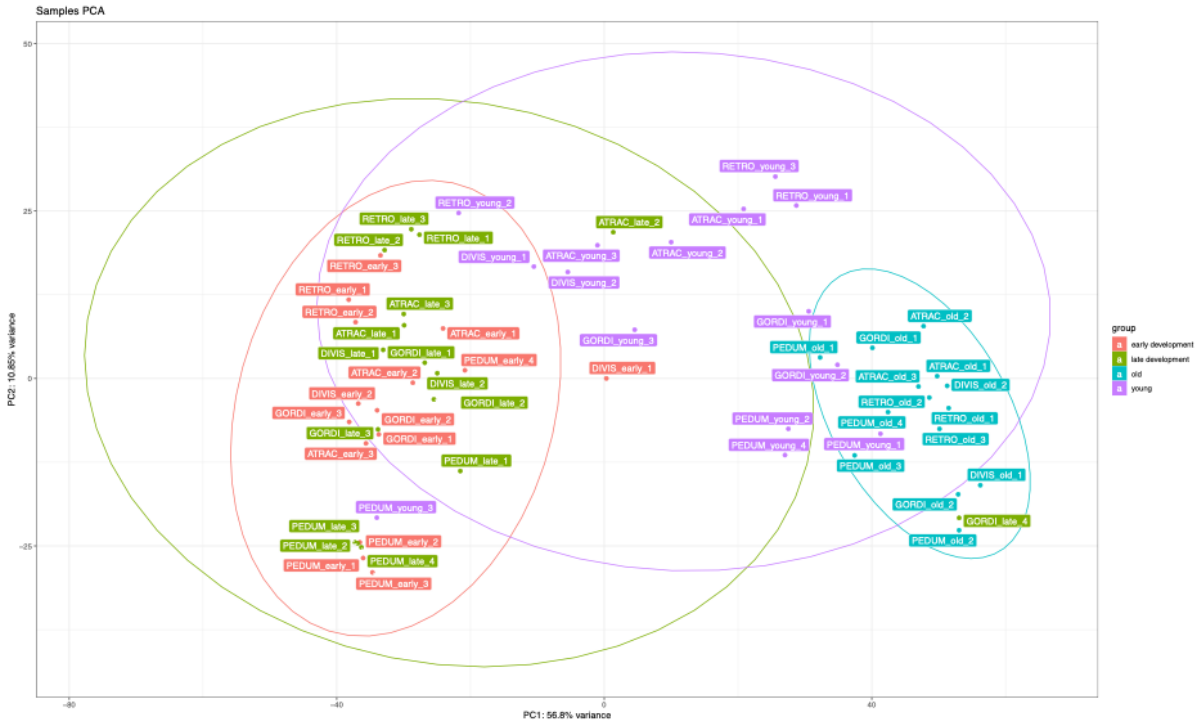

Fig. S57. Principal component analysis of transcriptomic profiles across four developmental stages in five *Scalesia* species. This figure displays a principal component analysis (PCA) plot representing the transcriptomic variation among four developmental stages (early development, late development, young leaf) of five Scalesia species: *S. atractyloides* (ATRAC), *S. retroflexa* (RETRO), *S. pedunculata* (PEDUM), *S. gordilloi* (GORDI), and *S. divisa* (DIVIS). Each point corresponds to an individual sample, labeled with the species abbreviation followed by the developmental stage and replicate number. The samples are color-coded by developmental stage: early development (red), late development (green), young leaf (purple), and old leaf (purple). Ellipses represent the 95% confidence intervals around the mean for each group.

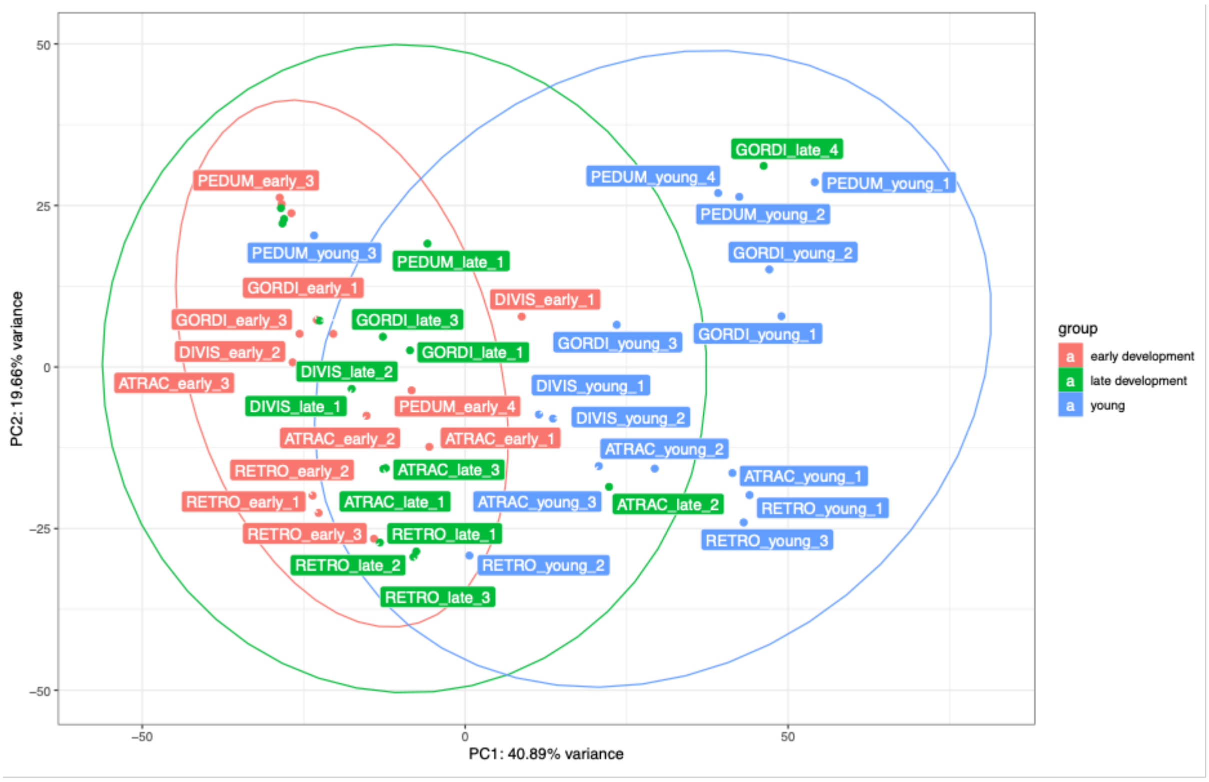

Fig. S58. Principal component analysis (PCA) of transcriptomic profiles across three developmental stages in five *Scalesia* species. This figure displays a PCA plot representing the transcriptomic variation among four developmental stages (early development, late development, young leaf) of five Scalesia species: *S. atractyloides* (ATRAC), *S. retroflexa* (RETRO), *S. pedunculata* (PEDUM), *S. gordilloi* (GORDI), and *S. divisa* (DIVIS). Each point corresponds to an individual sample, labeled with the species abbreviation followed by the developmental stage and replicate number. The samples are color-coded by developmental stage: early development (red), late development (green), and young leaf (blue). Ellipses represent the 95% confidence intervals around the mean for each group.

Supplementary References

1. Fernández-Mazuecos, M. *et al.* The Radiation of Darwin's Giant Daisies in the Galápagos Islands. *Curr. Biol.* **30**, 4989-4998.e7 (2020).
2. Eliasson, U. Studies in Galapagos plants. xIV. the genus Scalesia Arn. *Opera Bot.* (1974).
3. Nielsen, L. R. *et al.* Morphometric, AFLP and plastid microsatellite variation in populations of Scalesia divisa and S. incisa (Asteraceae) from the Galápagos Islands. *Bot. J. Linn. Soc.* **143**, 243–254 (2003).
4. Geist, D. J., McBIRNEY, A. R. & Duncan, R. A. Geology and petrogenesis of lavas from San Cristobal Island, Galapagos Archipelago. *Geol. Soc. Am. Bull.* **97**, 555 (1986).
5. Butlin, R. Speciation by reinforcement. *Trends Ecol. Evol.* **2**, 8–13 (1987).
6. Gillespie, R. G. *et al.* Comparing Adaptive Radiations Across Space, Time, and Taxa. *J. Hered.* **111**, 1–20 (2020).
7. Hamann, O. & Wium-Andersen, S. Scalesia gordilloi sp. nov. (Asteraceae) from the Galápagos Islands, Ecuador. *Nord. J. Bot.* **6**, 35–38 (1986).
8. Itow, S. Phytogeography and Ecology of Scalesia (Compositae) Endemic to the Galapagos Islands. *Pacific Science* **49**, 17–30 (1995).
9. Tye, A. & Loving, J. Scalesia pedunculata. *The IUCN Red List of Threatened Species* (1998) doi:10.2305/IUCN.UK.1998.RLTS.T30451A9551149.en.
10. Nielsen, L. R. Molecular differentiation within and among island populations of the endemic plant Scalesia affinis (Asteraceae) from the Galápagos Islands. *Heredity* **93**, 434–442 (2004).
11. Geist, D. J., Snell, H., Snell, H., Goddard, C. & Kurz, M. D. A paleogeographic model of the Galápagos islands and biogeographical and evolutionary implications. in *The Galápagos* 145–166 (John Wiley & Sons, Inc, Hoboken, New Jersey, 2014). doi:10.1002/9781118852538.ch8.
12. Bray, N. L., Pimentel, H., Melsted, P. & Pachter, L. Near-optimal probabilistic RNA-seq

quantification. *Nat. Biotechnol.* **34**, 525–527 (2016).

13. Ichihashi, Y. *et al.* Evolutionary developmental transcriptomics reveals a gene network module regulating interspecific diversity in plant leaf shape. *Proc. Natl. Acad. Sci. U. S. A.* **111**, E2616-21 (2014).
14. Jin, J. *et al.* PlantTFDB 4.0: toward a central hub for transcription factors and regulatory interactions in plants. *Nucleic Acids Res.* **45**, D1040–D1045 (2017).
15. Aibar, S. *et al.* SCENIC: single-cell regulatory network inference and clustering. *Nat. Methods* **14**, 1083–1086 (2017).
16. Marbach, D. *et al.* Wisdom of crowds for robust gene network inference. *Nat. Methods* **9**, 796–804 (2012).
17. Curci, P. L. *et al.* Identification of growth regulators using cross-species network analysis in plants. *Plant Physiol.* **190**, 2350–2365 (2022).
18. Shannon, P. *et al.* Cytoscape: a software environment for integrated models of biomolecular interaction networks. *Genome Res.* **13**, 2498–2504 (2003).
19. Sitaraman, J., Bui, M. & Liu, Z. LEUNIG_HOMOLOG and LEUNIG perform partially redundant functions during Arabidopsis embryo and floral development. *Plant Physiol.* **147**, 672–681 (2008).
20. Schulze, S., Schäfer, B. N., Parizotto, E. A., Voinnet, O. & Theres, K. LOST MERISTEMS genes regulate cell differentiation of central zone descendants in Arabidopsis shoot meristems: LOM genes control meristem maintenance. *Plant J.* **64**, 668–678 (2010).
21. Nakayama, H. *et al.* Regulation of the KNOX-GA gene module induces heterophyllic alteration in North American lake cress. *Plant Cell* **26**, 4733–4748 (2014).
22. Chung, Y. *et al.* Auxin Response Factors promote organogenesis by chromatin-mediated repression of the pluripotency gene SHOOTMERISTEMLESS. *Nat. Commun.* **10**, 886 (2019).
23. Mathieson, I. The omnigenic model and polygenic prediction of complex traits. *Am. J. Hum. Genet.* **108**, 1558–1563 (2021).

24. Boyle, E. A., Li, Y. I. & Pritchard, J. K. An expanded view of complex traits: From polygenic to omnigenic. *Cell* **169**, 1177–1186 (2017).

25. Liu, X., Li, Y. I. & Pritchard, J. K. Trans effects on gene expression can drive omnigenic inheritance. *Cell* **177**, 1022-1034.e6 (2019).

26. Fagny, M. & Austerlitz, F. Polygenic adaptation: Integrating population genetics and gene regulatory networks. *Trends Genet.* **37**, 631–638 (2021).

27. Stahle, M. I., Kuehlich, J., Staron, L., von Arnim, A. G. & Golz, J. F. YABBYs and the transcriptional corepressors LEUNIG and LEUNIG_HOMOLOG maintain leaf polarity and meristem activity in Arabidopsis. *Plant Cell* **21**, 3105–3118 (2009).

28. Iwasaki, M. *et al.* Dual regulation of ETTIN (ARF3) gene expression by AS1-AS2, which maintains the DNA methylation level, is involved in stabilization of leaf adaxial-abaxial partitioning in Arabidopsis. *Development* **140**, 1958–1969 (2013).

29. Sarojam, R. *et al.* Differentiating Arabidopsis shoots from leaves by combined YABBY activities. *Plant Cell* **22**, 2113–2130 (2010).

30. Fukushima, K. & Hasebe, M. Adaxial-abaxial polarity: the developmental basis of leaf shape diversity: development and evolution of leaf types. *Genesis* **52**, 1–18 (2014).

31. Vogel, S. Convective Cooling at Low Airspeeds and the Shapes of Broad Leaves. *J. Exp. Bot.* **21**, 91–101 (1970).

32. Eskildsen, L. I., Olesen, J. M. & Jones, C. G. Feeding response of the Aldabra giant tortoise (*Geochelone gigantea*) to island plants showing heterophylly. *J. Biogeogr.* **31**, 1785–1790 (2004).

33. Burns, K. C. *Evolution in Isolation: The Search for an Island Syndrome in Plants*. (Cambridge University Press, 2019).

34. Nicholls, H. *The Galápagos: A Natural History*. (Basic Books (AZ), 2014).

35. Traveset, A. *et al.* Galápagos land iguana (Conolophus subcristatus) as a seed disperser. *Integr. Zool.* **11**, 207–213 (2016).

36. Blake, S., Tapia, P. I., Safi, K. & Ellis-Soto, D. Chapter 11 - Diet, behavior, and activity patterns. in *Galapagos Giant Tortoises* (eds. Gibbs, J. P., Cayot, L. J. & Aguilera, W. T.)

207–239 (Academic Press, 2021). doi:10.1016/B978-0-12-817554-5.00025-3.

37. Philipp, M. & Nielsen, L. R. Reproductive ecology of Scalesia cordata (Asteraceae), an endangered species from the Galápagos Islands. *Bot. J. Linn. Soc.* **162**, 496–503 (2010).

38. Nielsen, L. R., Siegismund, H. R. & Philipp, M. Partial self-incompatibility in the polyploid endemic species Scalesia affinis (Asteraceae) from the Galápagos: remnants of a self-incompatibility system? *Bot. J. Linn. Soc.* **142**, 93–101 (2003).

39. Nielsen, L. R., Philipp, M., Adsersen, H. & Siegismund, H. R. Breeding system of Scalesia divisa Andersson, an endemic Asteraceae from the Galápagos Islands. *Norske Videnskaps-Akademi. I, Mat. -naturv. klasse. Skrifter, Ny serie* (2000).

40. Sambatti, J. B. M., Strasburg, J. L., Ortiz-Barrientos, D., Baack, E. J. & Rieseberg, L. H. Reconciling extremely strong barriers with high levels of gene exchange in annual sunflowers. *Evolution* **66**, 1459–1473 (2012).
