# Supplementary material for: The genomic basis of adaptive leaf variation in the Galápagos giant daisies
Source: Nat Commun. 2026 Apr 16;17:5319. doi: 10.1038/s41467-026-71865-3 (PMC13273160; doi:10.1038/s41467-026-71865-3)
Supplement: Supplementary file 2 — Description of Additional Supplementary Files [file 41467_2026_71865_MOESM2_ESM.pdf]

Description of Additional Supplementary Files

Supplementary Data 1:

Metadata information of DNA samples. This includes sample ID, population ID, species assignment, Galapagos island the sample was collected on, collection date, latitude and longitude of the collection location, mean sequencing depth, habitat and climate information of the collection location, comments if and why samples were removed from certain analysis, and accession numbers to raw sequencing files deposited to ENA. The geographic locations (latitude and longitude) have been rounded to one decimal point to protect the sampled populations of endangered species.

Supplementary Data 2:

Metadata of populations included in the DNA and/or leaf morphology analysis. This includes population ID, species assignment, collection year, Galapagos island the sample was collected on, latitude and longitude of the collection location, if the population was used for DNA sequencing and/or leaf morphology measurements and the number of individuals per population. The geographic locations (latitude and longitude) have been rounded to one decimal point to protect the sampled populations of endangered species.

Supplementary Data 3:

Leaf morphology measurements. This includes the raw measurements of LeafArea, Perimeter, VertLength, HorizWidth, AvgHorizWidth, AspectRation.W.L., FormCoefficient, BladeLength, MaxPerpWidth, PosiMaxPerpWidth, PerpWidth1, PerpWidth2, PetioleLength, and PetioleArea as well as the ratios Perimeter/BladeLength and Perimeter/LeafArea.

Supplementary Data 4:

F_{ST} outlier windows between lobed species and *Scalesia crockeri*. This includes the scaffold ID as named in the reference genome fasta file, the chromosome number (ranging from 1-34 ordered by length with chromosome 1 being the longest), the mid-position of the outlier window, F_{ST} value of the window, the Z score of the F_{ST} window, Fay and Wu's H for the lobed *Scalesia* species and for *Scalesia crockeri* in the window, *Scalesia* gene IDs of leaf development genes within/overlapping with the window, the *Arabidopsis thaliana* ortholog ID of the *Scalesia* gene and the *A. thaliana* gene symbol.

Supplementary Data 5:

Potentially selected leaf development genes in lobed-leaf *Scalesia* species. The table includes the *Arabidopsis thaliana* ortholog ID, the *A. thaliana* gene symbol, the *Scalesia* gene IDs, the *Scalesia* chromosome ID and chromosome number, and the lobed-leaf *Scalesia* species the gene is potentially under selection in.

Supplementary Data 6:

Scalesia genes overlapping with GWAS outlier SNPs. The table includes the *Scalesia* gene ID, the start and stop position of the gene on the Scalesia genome (range), the *Scalesia* chromosome ID and chromosome number, the corresponding *Arabidopsis thaliana* ortholog ID and gene symbol.

Supplementary Data 7:

Overview of RNA sequencing samples. This table includes the RNA sample ID, the species name, the tissue type sampled, the accession code of the living specimen in Copenhagen University Botanical Garden, the ENA accession

Supplementary Data 8:

Table that contains the height in m of the lobed *Scalesia* species. Values are taken from Eliasson, U. Studies in Galapagos plants. xIV. the genus Scalesia Arn. *Opera Bot.* (1974).

Supplementary Data 9:

Comparisons of F_{ST} outlier analysis between lobed-leaf species and the unlobed *S. crockery*. Table contains the species/population name and the sample size.

Supplementary Data 10:

List of genes associated with leaf-development (LCgenes2.0). Table contains the *Arabidopsis thaliana* gene ID, the gene name, gene type, a short description and a curator summary.

Supplementary Data 11:

List of *Scalesia* genes associated with leaf-development. The table contains the *Scalesia* gene ID of orthologs of the LCgenes2.0 (see Supplementary Data 10), the chromosome the gene is located on, the start and stop position of the gene on the *Scalesia* genome, a description of the gene and the associated GO terms.

Supplementary Data 12:

Description of the leaf morphology measurements.
