# Supplementary material for: The genomic basis of adaptive leaf variation in the Galápagos giant daisies
Source: Nat Commun. 2026 Apr 16;17:5319. doi: 10.1038/s41467-026-71865-3 (PMC13273160; doi:10.1038/s41467-026-71865-3)
Supplement: Supplementary file 15 — Reporting Summary [file 41467_2026_71865_MOESM15_ESM.pdf]

nature portfolio

Corresponding author(s): Michael D. Martin, Vanessa C. Bieker

Last updated by author(s): Feb 4, 2026

Reporting Summary

Nature Portfolio wishes to improve the reproducibility of the work that we publish. This form provides structure for consistency and transparency in reporting. For further information on Nature Portfolio policies, see our Editorial Policies and the Editorial Policy Checklist.

Statistics

For all statistical analyses, confirm that the following items are present in the figure legend, table legend, main text, or Methods section.

n/a	Confirmed	
☐	☒	The exact sample size (*n*) for each experimental group/condition, given as a discrete number and unit of measurement
☐	☒	A statement on whether measurements were taken from distinct samples or whether the same sample was measured repeatedly
☐	☒	The statistical test(s) used AND whether they are one- or two-sided<br>*Only common tests should be described solely by name; describe more complex techniques in the Methods section.*
☐	☒	A description of all covariates tested
☒	☐	A description of any assumptions or corrections, such as tests of normality and adjustment for multiple comparisons
☐	☒	A full description of the statistical parameters including central tendency (e.g. means) or other basic estimates (e.g. regression coefficient) AND variation (e.g. standard deviation) or associated estimates of uncertainty (e.g. confidence intervals)
☐	☒	For null hypothesis testing, the test statistic (e.g. *F*, *t*, *r*) with confidence intervals, effect sizes, degrees of freedom and *P* value noted<br>*Give P values as exact values whenever suitable.*
☒	☐	For Bayesian analysis, information on the choice of priors and Markov chain Monte Carlo settings
☒	☐	For hierarchical and complex designs, identification of the appropriate level for tests and full reporting of outcomes
☒	☐	Estimates of effect sizes (e.g. Cohen's *d*, Pearson's *r*), indicating how they were calculated

Our web collection on statistics for biologists contains articles on many of the points above.

Software and code

Policy information about availability of computer code

Data collection	WinFOLIA Pro vXXX
Data analysis	paleomix v1.2.14<br>AdapterRemoval v2.3.1<br>AdapterRemoval v2.3.2<br>BWA v0.7.17<br>picardtools v2.25.5<br>BLAST+ v2.9<br>R v4.3.1<br>R v4.2.2<br>GATK v3.7-0<br>GATK v4.2.3<br>bedtools v2.26<br>bedtools v2.30<br>angsd v0.935<br>angsd v.0.941<br>Plink v1.9<br>ggplot2 v3.4.2<br>PCangsd v0.98<br>PCangsd v1.10<br>NGSadmix

Degeneracy
calcDxy: https://github.com/mfumagalli/ngsPopGen/blob/master/scripts/calcDxy.R
R package GenomicRanges
vcftools v0.1.17
IQ-Tree 2
ASTRAL-III
Dsuite
PSMC
samtools v. 1.10
bcftools v.1.10
Bitacora v1.3
Trimmomatic v0.39
Kallisto v0.48.0
pcaExplorer v2.24.0
GENIE3 v1.20.0
Cytoscape v3.10.1
Cytoscape stringApp 2.1
R package MorphoTools2
The scripts and commands that call these tools and the Cytoscape analysis file are available on Dryad (https://doi.org/10.5061/dryad.j9kd51cr0).

For manuscripts utilizing custom algorithms or software that are central to the research but not yet described in published literature, software must be made available to editors and reviewers. We strongly encourage code deposition in a community repository (e.g. GitHub). See the Nature Portfolio guidelines for submitting code & software for further information.

Data

Policy information about availability of data

All manuscripts must include a data availability statement. This statement should provide the following information, where applicable:

- Accession codes, unique identifiers, or web links for publicly available datasets
- A description of any restrictions on data availability
- For clinical datasets or third party data, please ensure that the statement adheres to our policy

DNA sequencing data generated for this study have been deposited in the European Nucleotide Archive under the study accession code PRJEB70770 (https://www.ebi.ac.uk/ena/browser/view/PRJEB70770). The RNA sequencing data generated for this study have been deposited in the European Nucleotide Archive under the study accession code PRJEB74314 (https://www.ebi.ac.uk/ena/browser/view/PRJEB74314). A complete list of accession codes for each sample can be found in Table S1 (DNA) and Table S7 (RNA). The previously published reference genome of Scalesia atractyloides used in this study can be found on Dryad (https://doi.org/10.5061/dryad.8gtht76rh). The leaf morphology measurements generated for this study can be found in Table S3.

Research involving human participants, their data, or biological material

Policy information about studies with human participants or human data. See also policy information about sex, gender (identity/presentation), and sexual orientation and race, ethnicity and racism.

Reporting on sex and gender	*Use the terms sex (biological attribute) and gender (shaped by social and cultural circumstances) carefully in order to avoid confusing both terms. Indicate if findings apply to only one sex or gender; describe whether sex and gender were considered in study design; whether sex and/or gender was determined based on self-reporting or assigned and methods used. Provide in the source data disaggregated sex and gender data, where this information has been collected, and if consent has been obtained for sharing of individual-level data; provide overall numbers in this Reporting Summary. Please state if this information has not been collected. Report sex- and gender-based analyses where performed, justify reasons for lack of sex- and gender-based analysis.*
Reporting on race, ethnicity, or other socially relevant groupings	*Please specify the socially constructed or socially relevant categorization variable(s) used in your manuscript and explain why they were used. Please note that such variables should not be used as proxies for other socially constructed/relevant variables (for example, race or ethnicity should not be used as a proxy for socioeconomic status). Provide clear definitions of the relevant terms used, how they were provided (by the participants/respondents, the researchers, or third parties), and the method(s) used to classify people into the different categories (e.g. self-report, census or administrative data, social media data, etc.) Please provide details about how you controlled for confounding variables in your analyses.*
Population characteristics	*Describe the covariate-relevant population characteristics of the human research participants (e.g. age, genotypic information, past and current diagnosis and treatment categories). If you filled out the behavioural & social sciences study design questions and have nothing to add here, write "See above."*
Recruitment	*Describe how participants were recruited. Outline any potential self-selection bias or other biases that may be present and how these are likely to impact results.*
Ethics oversight	*Identify the organization(s) that approved the study protocol.*

Note that full information on the approval of the study protocol must also be provided in the manuscript.

Field-specific reporting

Please select the one below that is the best fit for your research. If you are not sure, read the appropriate sections before making your selection.

☐ Life sciences ☐ Behavioural & social sciences ☒ Ecological, evolutionary & environmental sciences

For a reference copy of the document with all sections, see nature.com/documents/nr-reporting-summary-flat.pdf

Ecological, evolutionary & environmental sciences study design

All studies must disclose on these points even when the disclosure is negative.

Study description	The study investigates the genomic and developmental basis of adaptive leaf shape variation, particularly leaf lobing, in the Galápagos endemic plant radiation Scalesia. It integrates population genomics, morphometrics, and transcriptomics to test whether repeated evolution of lobed leaves is adaptive and whether it arises from shared or distinct genetic mechanisms.<br><br>The primary quantitative dataset consists of whole-genome resequencing from 396 individuals representing all 15 recognized Scalesia species, including multiple populations per species and several putative hybrid populations. Sampling therefore follows a hierarchical population structure with individuals nested within populations and populations nested within species. Sequencing depth averages ~4.8×, with selected higher-coverage individuals used for demographic and phylogenomic analyses. Comparative genomic analyses include interspecific contrasts between lobed and unlobed taxa, effectively treating leaf phenotype as a focal factor across the phylogeny. Selection scans (e.g., FST outlier windows, Fay and Wu's H) and GWAS test associations between genomic regions and the lobed phenotype, incorporating population structure covariates.<br><br>Leaf morphometrics provide a second quantitative layer, measuring 16 morphological traits from multiple individuals per population across most species. This component also follows a nested design with leaves sampled from individuals within populations within species, enabling multivariate analyses (e.g., PCA) contrasting lobed versus unlobed phenotypic classes.<br><br>Transcriptomic analyses examine gene expression during four developmental stages in seven species differing in leaf phenotype. Here, developmental stage and phenotype function as treatment factors, with expression networks inferred within species and compared across lobed and unlobed groups. Replication derives from multiple individuals per species and multiple sampled leaves per developmental stage.<br><br>Overall, the design is integrative and hierarchical rather than manipulative: individuals are nested within populations and species, with comparative factors including leaf phenotype (lobed vs unlobed), phylogenetic lineage, and leaf developmental stage.
Research sample	The research sample consists of wild individuals from the Galápagos endemic plant radiation Scalesia (Asteraceae), encompassing all 15 recognized species, including subspecies and varieties. Plants were sampled from natural populations across multiple islands and habitat zones (arid, transition, humid) within Galapagos National Park, Ecuador, with ~5–10 individuals collected per population and multiple populations represented per species, yielding 396 resequenced genomes after filtering. The sample is intended to represent the standing genomic and phenotypic diversity of the extant radiation.<br><br>Individuals were mature, reproductively developed plants to ensure stable morphology; sex was not a factor because Scalesia are hermaphroditic, and precise ages were not determined. No field manipulations were performed beyond tissue and leaf collection.<br><br>Morphometric sampling included multiple individuals per population (typically 10–12), with five mature leaves measured per plant to quantify leaf shape variation. Transcriptomic analyses used a smaller subset of lobed and unlobed species grown under controlled conditions, with developing leaves sampled across ontogenetic stages to characterize gene expression dynamics.<br><br>The sampling design was chosen to maximize phylogenetic, ecological, and phenotypic coverage while retaining population-level replication, enabling inference on convergent leaf evolution and its genomic and developmental basis. Existing genomic resources from Scalesia and related Asteraceae served as reference datasets for comparative and annotation analyses.
Sampling strategy	Sampling followed a stratified, hierarchical field design rather than one based on formal a priori power calculations. Individuals were collected across the full taxonomic and geographic breadth of Scalesia, with multiple populations sampled per species where feasible and ~5–10 individuals per population, yielding 396 resequenced genomes after filtering. Sample sizes were determined by logistical access, permitting constraints, conservation considerations for rare taxa, and the need to balance phylogenetic breadth with within-population replication. These numbers are sufficient for estimating allele frequencies, resolving population structure, conducting FST outlier scans, and performing genotype–phenotype association using genotype-likelihood approaches suited to low-coverage data.<br><br>Morphometric sampling included 10–12 individuals per population with five mature leaves per plant, providing adequate replication to capture within- and among-population variance for multivariate trait analyses. Transcriptomic sampling used a smaller, targeted design spanning lobed and unlobed species and multiple developmental stages, with replication set to support differential expression and gene network inference while remaining tractable given limited available living material and sequencing costs.
Data collection	Data were collected through a combination of field sampling, laboratory processing, and controlled growth experiments. Leaf tissue and morphological specimens were collected in situ from wild Scalesia populations by the study authors and field collaborators under Galápagos research permits. For genomics, leaf tissue was preserved for DNA extraction, library preparation, and whole-genome resequencing performed by members of the research team and the lab team at the Novogene Europe sequencing facility. Morphometric data were recorded by study author Lene Nielsen and a technician by measuring multiple mature leaves per individual using standardized imaging and digital trait extraction protocols. For transcriptomics, plants from selected lobed and unlobed species were cultivated under controlled greenhouse conditions by the University of Copenhagen Botanical Garden staff, with developing

leaves harvested by Michael D. Martin and Lene Nielsen at defined stages for RNA extraction and sequencing. All datasets were generated and curated by the investigators using established genomic and phenotypic data acquisition pipelines.

Timing and spatial scale

Field sampling of wild Scalesia populations occurred between 1998 and 2004, during which fresh leaf material and pressed leaves were collected from naturally occurring populations across the Galápagos archipelago. Sampling was spatially extensive rather than temporally repeated, with each population typically sampled once; this reflects the long-lived nature of the plants and the goal of capturing standing genetic and phenotypic variation rather than temporal dynamics. A subset of these archived samples was selected in 2020 for whole-genome resequencing. Leaf material used for morphometric analyses was collected primarily between 1999 and 2004, with occasional supplementation from later collections at the same or nearby sites when original material was unavailable.

Transcriptomic data were collected in a separate sampling period from living Scalesia individuals grown ex-situ in botanical garden greenhouses, with leaf tissues harvested during a single experimental campaign spanning defined developmental stages. This later sampling phase is temporally distinct from the field collections and was designed to control environmental variation during gene expression measurements.

The spatial scale of the study encompasses the full Galápagos archipelago, including multiple islands and habitat zones (dry lowlands, transition zones, humid highlands), with data representing populations distributed across the range of the genus.

Data exclusions

Data exclusions were applied according to pre-established quality and independence criteria. In the genomic dataset, one resequenced individual was excluded due to insufficient sequencing depth, and four individuals were excluded because they were identified as first- or second-degree relatives of other samples, to avoid pseudoreplication. In addition, genomic sites in regions of low mapping quality, excessive sequencing depth, or poor mappability were excluded prior to analysis, as were variants failing standard base quality, mapping quality, coverage, or minor allele frequency thresholds; these filters were defined in advance and applied uniformly.

For morphometric analyses, leaves that were damaged, poorly pressed, incomplete, or not comparable across individuals were excluded to ensure reliable trait measurement. For transcriptomic analyses, mature leaf samples were excluded from network inference to focus on developmental stages relevant to leaf shape formation. All exclusions followed standard, predefined criteria aimed at data quality and analytical validity rather than post hoc outcome-based decisions.

Reproducibility

Reproducibility was addressed through replication across biological units, independent analytical contrasts, and methodological cross-validation rather than repeated laboratory experiments on the same samples. Population genomic inferences were supported by multiple individuals per population, multiple populations per species, and repeated independent contrasts between lobed and unlobed lineages. Phylogenetic results were verified by repeating ASTRAL species tree inference with three independent random sets of genes, all yielding the same topology with high support. Signals of selection and convergence were evaluated across independent evolutionary origins of leaf lobing rather than a single comparison.

Morphometric results were based on replicated measurements across multiple leaves per individual and multiple individuals per population, producing consistent separation of lobed and unlobed phenotypes in multivariate space. Transcriptomic findings were supported by biological replication across individuals, consistency across developmental stages, and concordance between selection scans and gene network analyses. No attempts to repeat analyses or computational experiments failed; all repeated analyses produced qualitatively consistent results.

Randomization

Samples were not randomly allocated to groups because this was an observational evolutionary study of naturally occurring populations rather than a manipulative experiment. Individuals were grouped post hoc based on species identity, population of origin, phylogenetic relationships, and leaf phenotype (lobed versus unlobed), all of which are inherent biological attributes.

Potential confounding due to non-random structure was addressed analytically rather than through randomization. Population structure and relatedness were explicitly modeled using genotype likelihood–based PCA, admixture analyses, and kinship estimation, with close relatives excluded to avoid pseudoreplication. In genotype–phenotype association analyses, population structure was controlled by including principal components as covariates. Comparative analyses were restricted to closely related taxa to minimize phylogenetic confounding, and multiple independent lobed lineages were analyzed to distinguish convergent evolution from shared ancestry. Because no experimental treatments or participant assignment occurred, random allocation was not applicable.

Blinding

Blinding was not implemented because the study was observational and comparative rather than experimental, and group identities such as species, population, and leaf phenotype are intrinsic, visually apparent attributes of the samples. During field sampling and morphometric measurement, leaf lobing is directly observable and cannot be concealed from the researcher. Genomic and transcriptomic data generation relied on automated sequencing and standardized bioinformatic pipelines, which minimize subjective influence during data acquisition. Statistical analyses were applied uniformly using predefined criteria and computational workflows, reducing the potential for observer bias. Because no treatments were assigned and no subjective outcome scoring was involved, blinding was not considered relevant or feasible for this study.

Did the study involve field work? ☒ Yes ☐ No

Field work, collection and transport

Field conditions

Field work was conducted under natural environmental conditions across the Galápagos archipelago, spanning arid lowlands, transition zones, and humid highland habitats. Sampling sites therefore encompassed broad climatic variation typical of the islands, with lowland sites characterized by hot, dry conditions and low annual rainfall, and highland sites by cooler temperatures, higher humidity, and substantially greater precipitation. Collections occurred during standard field seasons when plants were accessible and leaves were fully developed, but no experimental manipulation of environmental conditions was performed. Because the study focused on spatial ecological variation rather than short-term environmental effects, fine-scale measurements of temperature or

	rainfall at the time of collection were not required; instead, long-term habitat classifications (dry, transition, humid) were used to contextualize field conditions.
Location	Sampling was conducted across the Galápagos archipelago (Ecuador), spanning multiple islands including lowland and highland sites. Field locations covered a broad elevational range from near sea level in arid coastal zones to highland forests above 500–800 m elevation, depending on island and habitat. Sites encompassed dry lowlands, transition zones, and humid highlands, capturing the full environmental range occupied by Scalesia. Precise latitude and longitude coordinates and elevation data were recorded for each population and are reported in the associated sample metadata.
Access & import/export	All field sampling and subsequent analyses were conducted in compliance with local, national, and international regulations governing research in the Galápagos. Collection of wild Scalesia material was carried out under permits issued by the Ecuadorian Institute of Forestry, Natural Areas, and Wildlife (permit PC-001/98 PNG), authorizing plant sampling from natural populations between 1998 and 2004. Subsequent additional fieldwork and genetic analyses of these materials, including the use of herbarium specimens from the Charles Darwin Research Station Herbarium, were conducted under research permit MAAE-DBI-CM-2021-0213 issued by the Ecuadorian Ministry of the Environment, Water, and Ecological Transition. In most cases, a trained Galapagos National Park ranger was present during the fieldwork.<br><br>Sampling efforts were designed to minimize disturbance, with limited numbers of leaves collected per individual and whole-population sampling avoided except where populations were very small. Export of plant material for downstream laboratory work was performed in accordance with Ecuadorian regulations and institutional agreements, and all samples were handled through established scientific and herbarium channels. Ex situ plant material used for transcriptomic analyses was maintained in botanical garden collections under institutional oversight.
Disturbance	Disturbance caused by the study was minimal and limited to the removal of small amounts of leaf tissue. Sampling involved collecting only a few leaves per individual, avoiding damage to stems or reproductive structures, and whole-population sampling was avoided except in very small populations where it was unavoidable. No plants were removed or destroyed, and no habitat modification occurred. Sampling intensity was adjusted to population size to reduce impacts on rare or threatened taxa, and the use of herbarium specimens and ex situ collections further minimized additional field disturbance.

Reporting for specific materials, systems and methods

We require information from authors about some types of materials, experimental systems and methods used in many studies. Here, indicate whether each material, system or method listed is relevant to your study. If you are not sure if a list item applies to your research, read the appropriate section before selecting a response.

Materials & experimental systems

n/a	Involved in the study	
☒	☐	Antibodies
☒	☐	Eukaryotic cell lines
☒	☐	Palaeontology and archaeology
☒	☐	Animals and other organisms
☒	☐	Clinical data
☒	☐	Dual use research of concern
☐	☒	Plants

Methods

n/a	Involved in the study	
☒	☐	ChIP-seq
☒	☐	Flow cytometry
☒	☐	MRI-based neuroimaging

Dual use research of concern

Policy information about [dual use research of concern](#)

Hazards

Could the accidental, deliberate or reckless misuse of agents or technologies generated in the work, or the application of information presented in the manuscript, pose a threat to:

No	Yes	
☒	☐	Public health
☒	☐	National security
☒	☐	Crops and/or livestock
☒	☐	Ecosystems
☒	☐	Any other significant area

Experiments of concern

Does the work involve any of these experiments of concern:

No	Yes	
☒	☐	Demonstrate how to render a vaccine ineffective
☒	☐	Confer resistance to therapeutically useful antibiotics or antiviral agents
☒	☐	Enhance the virulence of a pathogen or render a nonpathogen virulent
☒	☐	Increase transmissibility of a pathogen
☒	☐	Alter the host range of a pathogen
☒	☐	Enable evasion of diagnostic/detection modalities
☒	☐	Enable the weaponization of a biological agent or toxin
☒	☐	Any other potentially harmful combination of experiments and agents

Plants

Seed stocks	Plant material used in this study came from two main sources: wild field collections and ex situ living collections. Field-collected material consisted of leaf tissue and pressed leaf specimens from naturally occurring Scalesia populations across the Galápagos archipelago, collected between 1998 and 2004 under authorized research permits. Sampling locations spanned multiple islands and habitat zones, with leaves collected non-destructively from mature plants; collection dates and site metadata (including island, locality, elevation, and habitat type) were recorded for each population and are provided in the sample metadata. In cases where fresh material was unavailable, small leaf fragments were sampled from herbarium specimens housed at the Charles Darwin Research Station Herbarium. Material used for transcriptomic analyses was obtained from living Scalesia plants maintained in botanical garden collections, originating from earlier authorized collections in the Galápagos. No commercial seed stocks or seed stock centres were used, and no catalogue numbers apply.
Novel plant genotypes	Not applicable
Authentication	Not applicable
