# Supplementary material for: The genomic basis of adaptive leaf variation in the Galápagos giant daisies
Source: Nat Commun. 2026 Apr 16;17:5319. doi: 10.1038/s41467-026-71865-3 (PMC13273160; doi:10.1038/s41467-026-71865-3)
Supplement: Supplementary file 16 — Transparent Peer Review file [file 41467_2026_71865_MOESM16_ESM.pdf]

nature portfolio

Peer Review File

The genomic basis of adaptive leaf variation in the Galápagos giant daisies

Corresponding Author: Dr Vanessa Bieker

This file contains all reviewer reports in order by version, followed by all author rebuttals in order by version.

Version 0:

Reviewer comments:

Reviewer #1

(Remarks to the Author)
This well-conceived, well-executed study represents a major step forward for understanding insular adaptive radiation through a convincing characterization of the importance of ancestral genomic variation in adaptation to different environments. The choice of a highly diverse insular clade as the research system and a distinctive morphological character (leaf lobing) associated with occurrence in contrasting habitats (and scattered across subclades) was ideal for examining the genomic basis for ecological change associated with diversification. The findings that lobed leaves were derived independently (convergently) in different subclades and that the different genomic variants associated with leaf lobing could be inferred to be ancestral in Scalesia allow for the authors to conclude that repeated insular adaptation in this instance was not a result of hybridization of radiating lineages, as previously concluded for Darwin's finches and African cichlids, but may still be attributable to variation (across subgenomes) acquired from ancestral hybridization (allopolyploidization), prior to radiation of the insular clade. Lack of evidence for introgression within the insular clade from the genomic analyses conducted by the authors lends further credibility to that conclusion.

These findings provide novel empirical evidence for the role that allopolyploidization can play in allowing for adaptation in the face of founder effects associated with long-distance dispersal and insular colonization, in keeping with the significantly higher incidence of polyploidy in endemic flowering plants of remote oceanic islands by comparison with flowering plants of continental or near-continental areas.

I find no problems with this study in the design, methodology, or interpretations of results and find it to be sufficiently detailed to allow for replication of the study. The manuscript is extremely well-written and organized and a pleasure to read. There are just a few minor points for consideration:

Lines 46 & 84-85: "Life habit" is an unusual construct and might be best restated as "life form" or merely "habit" to prevent confusion (e.g., in 84-85, perhaps change to "life form or habit"
Lines 149-150: "not yet managed to generate substantial diversity" warrants changing to "not yet managed to generate substantial genetic diversity" or something similar, to avoid the potential misinterpretation by readers that unqualified "diversity" refers to biodiversity or species diversity, which of course is exceptionally high in Scalesia for an insular clade.
Lines 160-161: "spawned three independent origins of leaf lobedness" would be best changed here to "spawned three origins of leaf lobedness" because the independent (convergent) basis of leaf lobedness that is so nicely shown in this study cannot be inferred directly from the phylogeny in the absence of the genomic analysis, which is introduced later in the manuscript.
Lines 271-273: This sentence, although perfectly reasonable, is stated in way that may warrant a supporting citation or callout (or to restate the sentence), if the authors agree it is warranted.
Lines 320-321: "… the enormous phenotypic diversity within the genus is at least partly adaptive and derives almost entirely from selection on standing genetic variation". This sentence is important for the conclusions but needs minor clarification to ensure that the point about how selection on standing genetic variation accounts almost entirely for adaptive phenotypic diversity is made more specific to leaf lobing or lack thereof. Otherwise, someone reading the conclusions in isolation of the rest of the study may come away thinking that the statement applies more generally to components of phenotypic diversity across Scalesia that were not investigated.
Lines 334-344: Although this last paragraph of the conclusions does not focus on the main findings of the study, these secondary findings about evidence for more biodiversity within Scalesia than is currently recognized taxonomically is

sufficiently important from a conservation standpoint that it should be retained here, in my opinion, especially given the extreme endangerment of oceanic island plant lineages in general.
Line 425: Best to spell out “linkage disequilibrium (LD)” at first usage of “LD” here.
Line 439-440: Best to insert “(SFS)” after “site frequency spectra” here, before use of abbreviation in isolation.
Line 496 & 497: Change “LTR” to “LRT” on each line here, with “likelihood ratio test (LRT)” spelled out at first usage.
Line 542: Change “once conducted” to “conducted once”

Bruce G. Baldwin

Reviewer #2

(Remarks to the Author)
Rapid adaptive radiations are a hot and very interesting topic in evolutionary biology. For a while I wondered why nobody studied evolutionary genetics of Scalesia radiation and I was very pleased and interested to see that finally this paper does just that. However, many aspects of the paper are badly in need to significant improvement.
Introduction and discussion need to do a better job with placing this study in the context of existing literature. Although the paper focuses on adaptation in a plant radiation, the previous studies of adaptation in plant radiations (e.g. Nevado et al 2016, 2019, 2024 [DOI: 10.1038/ncomms12384, DOI: 10.1016/j.cub.2019.07.059 and DOI: 10.1111/mec.17232]) are not even mentioned.
The description of the data in this manuscript is far from satisfactory. The paper focuses on evolutionary genetic analyses based on genetic diversity within and divergence between Scalesia species. Strangely enough, the levels and patterns of genetic diversity in different species are not described. How much DNA polymorphism (pi and theta) at silent sites is present in different species? How much sequence divergence is present between the species? They only report Fst but Fst is a relative measure (ratio of variation betweenSpecies/total) that tells us little about the actual species divergence – please report the actual divergence (Dxy and Da) at silent sites. The basic summary statistics for patterns of polymorphism (e.g. Tajima’s D and Kelly ZnS) are also not reported. How high is the extent of linkage disequilibrium (LD), how rapid is LD decay with distance along the genome? Without these basic statistics the reader has no way to assess genetic diversity and divergence underlying most analyses in this paper. There are many other points/criticisms below that the authors need to address. Some of these are only minor but some are major and represent significant problems that require reanalysis / rethinking / reinterpretation.
The paper does not clearly report the divergence (at silent sites, Ks) between the paralogous genes in Scalesia subgenomes – please report it! From the previous paper (Cerca et al 2022) it looks like polyploidisation is quire recent, ~4My, so I guess Ks between paralogs is only few percent. This means that distinguishing between these paralogs in read mapping and SNP calling is not easy, which is a major worry for me (and future readers) about the accuracy of SNP calling and possible artefacts originating from the mis-mapped reads between very similar paralogous copies of a gene. Such artefactual SNPs due to read mis-mapping would be expected to be present at high frequency, which may cause various biases and false signals, such as of population expansion in the past (peaks seen on FigS3). The problems with population genetic analyses of recently duplicated genes (as is the case in Scalesia) are common and not easy to resolve. The authors need to do a much better job with convincing the reader that they resolved these problems. Simply stating that their mapping quality >30 is certainly not enough as sequence reads originating from two different low divergent copies of a paralogous pair can be nearly identical and indistinguishable from genuine polymorphisms originating from two alleles of the same gene.
Fig 1A. Scalebar for branch length is shown but figure legend does not explain what king of distance is used in this tree.
Line 136 and elsewhere it is stated that samples were sequenced to “high-depth (>8X)”. Note that anything above 20X would be regarded as sufficiently high depth but 8X coverage is not at all “high depth”. Please correct this throughout the paper – avoid referring to low coverage (<10X) and medium coverage (10X<coverage<20X) samples as “high depth”.
Lines 141-145: Based on observation of short internal and long external branches the authors infer “increased speciation rates and decreased extinction rates relative to Scalesia’s early evolution”. However, such ‘star-like’ tree may reflect recent population expansion in many Scalesia species. Recent population expansion is not detectable on PSMC plots (FigS3) because this method has poor resolution for recent events (e.g. fig1 in DOI: 10.1111/2041-210x.13740). Another potential (artefactual) cause of short internal and long external branches in a phylogeny is recombination that specifically creates this bias (see Schierup and Hein 2000 Genetics v156: 879-891). Recombination would be particularly problematic for multigenic phylogeny reconstructions as done in this manuscript.
Lines 153-154: “using high depth (>12X, MAPQ30) samples”. Coverage and mapping quality varies across the SNPs. Perhaps “>12X, MAPQ30” refers to threshold used to filter SNPs before analysis rather than to samples? Please clarify.
Lines 155-156: PSMC plots from FigS3 give information about population size change but not about the time of species divergence. Convergence of such plots at some point in the past for two species does not mean that the two species diverged at that time point. Time of pairwise species divergence has to be analysed properly with sequence divergence and population split models.
Lines 218-219: Fay and Wu’s H (and other tests of neutrality) do not provide “evidence for selection”. They only show that the null model poorly fits the data. There are many possible causes for a test of neutrality to reject null model and it does not necessarily mean that selection caused the observed signal.
Line 224 and elsewhere in the paper. Please add “putative” before “selected gene”. The preceding paragraph explains why none of the identified candidate genes are proven to be under selection and the authors have to be careful about this.
Lines 239-241: Unclear why the authors conclude that different species use different genes to evolve serrated leaves (do a classic genetic complementation test? – cross two serrated species; if diff mutations are involved then the progeny should have non-dissected leaf). The signals of ‘selection’ (i.e. various outliers in Fst and FayWuH) they detect are not necessarily caused by leaf shape evolution – there are loads of various factors (e.g. adaptation to new strains of viruses or parasites)

that could have driven adaptation.
Line 319: Please add “some” before “leaf development genes” and add “likely” before “have been”
Line 321: How did the authors come to the conclusion that most adaptation occurred from standing variation?? Adaptation from standing variation leads to soft sweeps that are very difficult to detect. I did not see any convincing demonstration of adaptation from standing variation in the paper.
Line 331: Please remove “unprecedented”. Is this really unprecedented? Better avoid such poor journalistic stile in a scientific paper.
FigS3 legend. “Demographic reconstructions from past population sizes” please replace “from” with “of”. Also, please add to fig legend what approach was used – PSMC, MSMC2 or something else.

Reviewer #3

(Remarks to the Author)
The authors present a genomic study of the Asteraceae genus Scalesia, which includes morphologically diverse species that inhabit various islands of the Galapagos. Following from a previous study that sequenced the genome of one species, S. atractyloides, the authors utilize this resource to investigate the potential adaptive variation in leaf lobation in Scalesia species. They conduct shallow genome sequencing for all species, with multiple populations represented, conduct a morphometric study of leaf characters for the species, and undertake a leaf transcriptomic analysis of selected species. The paper overall is generally well written, but as there is a lot going on in the paper, I think it could be better structured to help the reader understand why certain approaches were undertaken. There are also several aspects that need to be further clarified given the genomic complexities of this system.

Line 73 – recommend some other word than crucible: location, region, etc.
Line 125 – Overall, the introduction should introduce the potential significance of leaf lobation better – as is found in the supplementary information. Many plants show flexibility in leaf lobation so it is unclear how this is perceived as an adaptive trait (leading to higher fitness, etc.).
Lines 142 – 145 ‘…clade c, which includes all species with lobed leaves…’ This phrasing is misleading, as this clade also includes unlobed species. Could these shorter branch lengths at the base of clade c relate to the species geography and island geology rather than leaf lobing as stated (Line 145)?
Line 383 – How much data were generated for each sample? This information is indicated for the transcriptome analyses (line 601) but not the genome sequencing.

Given that these are all polyploid species (n=34 according to the Chromosome counts database), how were homeologous sequences dealt with for the analyses? There is one brief mention about the subgenomes (line 503) for SNP discovery. How were homeologs vs paralogs dealt with for the phylogenetic analyses and the population genetic analyses? Can you be certain that you are analyzing gene variation vs allelic variation among all the samples?

Line 375 – change ‘were’ to ‘was’ at the end of the line
Line 440 – define SFS
Line 496 – define LTR
Line 542 – move once after conducted on its first use
Lines 915-921. Figure 1 – add asterisks to taxon names in A (phylogeny). It would be helpful to add asterisks to lobed species names in all of the figures. Delete ‘are’ in line 917. The symbols in C are really difficult to distinguish.

Version 2:

Reviewer comments:

Reviewer #1

(Remarks to the Author)
I was Reviewer #1 in the previous review of this manuscript and am completely satisfied with the responses to my review by the authors and with their revision of the manuscript, taking into account their responses to the other two reviewers, as well. I have nothing further to suggest toward revision of the manuscript, which I believe is in fine shape.
Bruce G. Baldwin

(Remarks on code availability)
It appears that the authors have provided what is necessary at DRYAD.

Reviewer #2

(Remarks to the Author)
The paper is somewhat improved after revision, but my concerns about selection analyses remain. The scan for selection used in this paper is relatively basic and it is prone to false positives due to sensitivity of Fay&Wu’s H to by mis-identification of ancestral SNP state, as explained in the original Fay and Wu’s paper (2020). There are many other approaches proposed more recently, such as Selscan (10.1093/bioinformatics/btae006), SweepFinder (10.1093/bioinformatics/btw051) and Raisd (10.1038/s42003-018-0085-8); at least one of these has to be added to the paper to make the analyses of selection more convincing.

I’m not convinced by their claim that adaptation occurs mainly from preexisting variation. Adaptation from preexisting variation should result in soft sweeps that are not easily detectable with such statistics as FayWuH. To assess the contribution of preexisting (standing) genetic variation to adaptation it is better to use the tests that were shown to be effective in detecting soft sweeps (e.g. 10.1534/genetics.118.301502). The (negative) peaks of FayWuH they see (Figs S14 to S23) are likely due to hard sweeps (FayWuH is more sensitive to such sweeps) driven by denovo mutations or variants introgressed from other species. In small populations, as is the case in Scalesia, rare introgression of adaptive alleles is expected to play larger role in adaptation compared to large population. Sweeps driven by introgressed alleles would also explain why in many cases putatively selected SNPs detected in Scalesia are present in the other species too. There are tests specifically designed to detect such sweeps due to introgressed alleles (e.g. Volcanofinder [Setter et al 2020 PlosG]). Adding this analysis to the paper would clarify the source of adaptive alleles contributing to adaptation in Scalesia.

Minor points:

In response to my request to remove “high” for seq depth >8, the authors changed “high” to “higher” throughout the paper. I do no think this is a satisfactory change. “Higher” than what? For example, the 1st sentence in Fig1 legend now reads “Multi-species coalescent phylogeny based on higher sequencing depth samples (> 8X)”. Why “high” or “higher” is needed here and elsewhere in the paper in the context of read depth? Why not simply give the information about read depth and let the reader decide whether it is high or not?

Thanks for clarifying distance units for branch length in fig1A legend. One other thing that is missing from the legend is the clarification of what the vertical lines with a,b,c,c-1… labels mean in fig1A.

For fig2C “subclades with green: clade c-1” I do not see any green bars or dots – only purple and various shades of grey in the pdf version I’ve got for review.

Line 164: “below 0.2 respectively 0.041” change to “below 0.2 and 0.041, respectively”

Lines 165-166: “Such FST values are typically considered moderate to high for differentiated populations within species, indicating that individual Scalesia species have not yet managed to generate substantial genetic diversity despite their success in adapting to various environments.” This is a surprising statement. Fst tells us little about diversity within individual species. Please refer to pi and theta levels here to support the statement that species have low diversity. Also, why “yet”? It implies that diversity is increasing, but no evidence for such increase over time is reported in the paper. Small island populations are expected to have small Ne and low genetic diversity, so pi~0.5% (FigS10c) may actually be regarded as relatively high genetic diversity for small island populations.

Line 191: “Low gene flow … facilitated by their nearly allopatric distribution” please rephrase to make it clear that allopatry leads to reduction in gene flow rather that facilitates it.

Figs S14 to S23. It would be really helpful to see plots of pi for each of the compared species along with Fst and FayWuH plots that are already shown. Sweep in one species should reduce polymorphism in that species and it would be reassuring to see this along with plots of Pst and FayWuH.

Line 463 and FigS30 legend. Please clarify which species was used for this analysis. Was it done for each of the species (then why a single LD decay plot shown)?

(Remarks on code availability)

Reviewer #3

(Remarks to the Author)

(Remarks on code availability)

Version 3:

Reviewer comments:

Reviewer #2

(Remarks to the Author)
I am satisfied with this revised version of the manuscript. I have only few minor points listed below.

Line 163: please correct “(FST > 0.25, Dxy > 0.42).” to “(FST > 0.25, Dxy > 0.042).” (Dxy is 10 times higher than it should be)

Suppl. Figures S14 to S23: please change the scale of Y-axis on all plots with pi – please limit it to 0.05 maximum. All

outliers >0.05 are likely artefacts – misalignment or mis-mapping of paralogous reads. Without this Y-scale adjustment all pi values are squeezed at the bottom of the plot, making pi-plots uninformative.

Lines 165-166: I think positive TajD is in species where >1 population was sampled – please check and clarify. Pooling slightly differentiated populations should result in inflated positive Tajima's D, while per-population TajD is likely often negative.

(Remarks on code availability)

Open Access This Peer Review File is licensed under a Creative Commons Attribution 4.0 International License, which permits use, sharing, adaptation, distribution and reproduction in any medium or format, as long as you give appropriate credit to the original author(s) and the source, provide a link to the Creative Commons license, and indicate if changes were made.
In cases where reviewers are anonymous, credit should be given to 'Anonymous Referee' and the source.
The images or other third party material in this Peer Review File are included in the article's Creative Commons license, unless indicated otherwise in a credit line to the material. If material is not included in the article's Creative Commons license and your intended use is not permitted by statutory regulation or exceeds the permitted use, you will need to obtain permission directly from the copyright holder.
To view a copy of this license, visit https://creativecommons.org/licenses/by/4.0/

Response to reviewers

I am satisfied with this revised version of the manuscript. I have only few minor points listed below.

Line 163: please correct “(FST > 0.25, Dxy > 0.42).” to “(FST > 0.25, Dxy > 0.042).” (Dxy is 10 times higher than it should be)

Thank you very much for spotting that typo. We have now corrected the value.

Suppl. Figures S14 to S23: please change the scale of Y-axis on all plots with pi – please limit it to 0.05 maximum. All outliers >0.05 are likely artefacts – misalignment or mis-mapping of paralogous reads. Without this Y-scale adjustment all pi values are squeezed at the bottom of the plot, making pi-plots uninformative.

Thank you for your comment. We have added additional panels to the SI figures with the y-axis limited to 0.05. We chose to keep the original panels without the y-axis limit to show the whole range of values obtained to not mislead readers.

Lines 165-166: I think positive TajD is in species where >1 population was sampled – please check and clarify. Pooling slightly differentiated populations should result in inflated positive Tajima’s D, while per-population TajD is likely often negative.(Remarks on code availability).

Thank you for your comment. That is an interesting point. We have now estimated Tajima’s D for all populations separately. Similar to the per-species estimates, we find that most populations have a positive Tajima’s D. For some species, some populations have a positive value while others are negative. We have added the per-population estimates as Figure S11.

REVIEWER COMMENTS

Reviewer #1 (Remarks to the Author):

This well-conceived, well-executed study represents a major step forward for understanding insular adaptive radiation through a convincing characterization of the importance of ancestral genomic variation in adaptation to different environments. The choice of a highly diverse insular clade as the research system and a distinctive morphological character (leaf lobing) associated with occurrence in contrasting habitats (and scattered across subclades) was ideal for examining the genomic basis for ecological change associated with diversification. The findings that lobed leaves were derived independently (convergently) in different subclades and that the different genomic variants associated with leaf lobing could be inferred to be ancestral in Scalesia allow for the authors to conclude that repeated insular adaptation in this instance was not a result of hybridization of radiating lineages, as previously concluded for Darwin's finches and African cichlids, but may still be attributable to variation (across subgenomes) acquired from ancestral hybridization (allopolyploidization), prior to radiation of the insular clade. Lack of evidence for introgression within the insular clade from the genomic analyses conducted by the authors lends further credibility to that conclusion.

These findings provide novel empirical evidence for the role that allopolyploidization can play in allowing for adaptation in the face of founder effects associated with long-distance dispersal and insular colonization, in keeping with the significantly higher incidence of polyploidy in endemic flowering plants of remote oceanic islands by comparison with flowering plants of continental or near-continental areas.

I find no problems with this study in the design, methodology, or interpretations of results and find it to be sufficiently detailed to allow for replication of the study. The manuscript is extremely well-written and organized and a pleasure to read. There are just a few minor points for consideration:

Thank you for the kind words. We are glad to hear that you enjoyed reading our manuscript and provided constructive feedback. Please find below point by point answers to the raised concerns.

R1-1: Lines 46 & 84-85: "Life habit" is an unusual construct and might be best restated as "life form" or merely "habit" to prevent confusion (e.g., in 84-85, perhaps change to "life form or habit")

Thank you for your comment. We have changed "Life habit" in the abstract to "habit" now and changed it in the introduction to "life form or habit" as suggested.

R1-2: Lines 149-150: "not yet managed to generate substantial diversity" warrants changing to "not yet managed to generate substantial genetic diversity" or something similar, to avoid the potential misinterpretation by readers that unqualified "diversity" refers to biodiversity or species diversity, which of course is exceptionally high in Scalesia for an insular clade.

We have added "genetic" now to avoid confusion with other types of diversity within the genus.

R1-3: Lines 160-161: "spawned three independent origins of leaf lobedness" would be best changed here to "spawned three origins of leaf lobedness" because the independent (convergent) basis of leaf lobedness that is so nicely shown in this study cannot be inferred directly from the phylogeny in the absence of the genomic analysis, which is introduced later in the manuscript.

Thank you for spotting that the independent origins are only shown later in the manuscript and should thus not be mentioned here yet. We have thus removed it now.

R1-4: Lines 271-273: This sentence, although perfectly reasonable, is stated in a way that may warrant a supporting citation or callout (or to restate the sentence), if the authors agree it is warranted.

Thank you for alerting us to this unclear statement. We have attempted to clarify the sentence by simplifying it. We also added a callout to Figure 4, which should also be more clarifying.

R1-5: Lines 320-321: "… the enormous phenotypic diversity within the genus is at least partly adaptive and derives almost entirely from selection on standing genetic variation". This sentence is important for the conclusions but needs minor clarification to ensure that the point about how selection on standing genetic variation accounts almost entirely for adaptive phenotypic diversity is made more specific to leaf lobing or lack thereof. Otherwise, someone reading the conclusions in isolation of the rest of the study may come away thinking that the statement applies more generally to components of phenotypic diversity across Scalesia that were not investigated.

Thank you for your comment. We agree that this sentence should be clarified to avoid that it will be read in a too general way. We have thus changed it to:
"They further indicate that the plants' enormous phenotypic diversity in leaf lobing is at least partly adaptive, reflecting past work that has shown positive selection to be more common in rapidly radiating plant lineages[37,38], and derives almost entirely from selection on standing genetic variation within leaf development genes.." (l. 341-344).

R1-6: Lines 334-344: Although this last paragraph of the conclusions does not focus on the main findings of the study, these secondary findings about evidence for more biodiversity within Scalesia than is currently recognized taxonomically is sufficiently important from a conservation standpoint that it should be retained here, in my opinion, especially given the extreme endangerment of oceanic island plant lineages in general.

Thank you for agreeing that this part should be retrained in the conclusion. We also felt that this is a very important finding for conservation that should not go unnoticed and thus decided to include it in the conclusion. We have changed the header of that section to "Conclusion and implications for conservation" to reflect that we do not just mention the main conclusions from the manuscript. (l. 339).

R1-7: Line 425: Best to spell out "linkage disequilibrium (LD)" at first usage of "LD" here.

Yes, you are right that LD should be defined at first mention. We have added this now (l. 453).

R1-8: Line 439-440: Best to insert "(SFS)" after "site frequency spectra" here, before use of abbreviation in isolation.

We have inserted "(SFS)" now as we indeed missed to define the abbreviation before its use (l. 481).

R1-9: Line 496 & 497: Change "LTR" to "LRT" on each line here, with "likelihood ratio test (LRT)" spelled out at first usage.

Thank you for spotting the accidental use of "LTR" instead of "LRT". We have corrected this now and spelled it out at its first use (l. 549).

R1-10: Line 542: Change "once conducted" to "conducted once"

We have corrected this now to "conducted once" (l. 596).

Bruce G. Baldwin

Reviewer #2 (Remarks to the Author):

Rapid adaptive radiations are a hot and very interesting topic in evolutionary biology. For a while I wondered why nobody studied evolutionary genetics of Scalesia radiation and I was very pleased and interested to see that finally this paper does just that. However, many aspects of the paper are badly in need to significant improvement.

R2-1: Introduction and discussion need to do a better job with placing this study in the context of existing literature. Although the paper focuses on adaptation in a plant radiation, the previous studies of adaptation in plant radiations (e.g. Nevado et al 2016, 2019, 2024 [DOI: 10.1038/ncomms12384, DOI: 10.1016/j.cub.2019.07.059 and DOI: 10.1111/mec.17232]) are not even mentioned.

Thank you for alerting us about these studies. It is indeed true that they were not previously mentioned in the Introduction. It should however be noted that we do cite literature of other plant radiations in the Introduction and Discussion, namely the Hawaiian silversword plants and the plant genus Metrosideros. We now added a very relevant reference to the Discussion where parallel evolution of leaf morphology was found in a sky island system in the *Viburnum* genus (l. 260-262) as well as the most relevant references suggested by the Reviewer in the Introduction (l. 126-128) and Discussion (l. 342-344).

R2-2: The description of the data in this manuscript is far from satisfactory. The paper focuses on evolutionary genetic analyses based on genetic diversity within and divergence between Scalesia species. Strangely enough, the levels and patterns of genetic diversity in different species are not described. How much DNA polymorphism (pi and theta) at silent sites is present in different species? How much sequence divergence is present between the species? They only report Fst but Fst is a relative measure (ratio of variation betweenSpecies/total) that tells us little about the actual species divergence – please report the actual divergence (Dxy and Da) at silent sites. The basic summary statistics for patterns of polymorphism (e.g. Tajima's D and Kelly ZnS) are also not reported. How high is the extent of linkage disequilibrium (LD), how rapid is LD decay with distance along the genome? Without these basic statistics the reader has no way to assess genetic diversity and divergence underlying most analyses in this paper. There are many other points/criticisms below that the authors need to address. Some of these are only minor but some are major and represent significant problems that require reanalysis / rethinking / reinterpretation.

In response to the reviewer's comments, we have now included an analysis of pi, theta, and Tajima's D at 4-fold degenerate sites. All of these data should then provide answers to the reviewer's fundamental questions about quantifying the genetic divergence within and between *Scalesia* species. The associated methods can be found in l.475-500. The plots are included as Fig. S10 and can also be found below for convenience. To add an absolute estimate of divergence between species, we now calculated d_{xy} at 4-fold degenerate sites and included it in Fig. S9 (together with the F_{ST} results, see below). The associated methods can be found in line l.475-500. It should be noted that d_{xy} and F_{ST} show similar results with species pairs having low F_{ST} also having low d_{xy} values. The new results have been incorporated into the main text (l. 161-171). We now also include a figure showing LD decay (Fig. S30, see below). The associated methods can be found in l. 455-463. The LD decay plot (Fig. 30) supports our choice of 10-kbp windows in the F_{ST} analysis as LD is broken down at this distance.

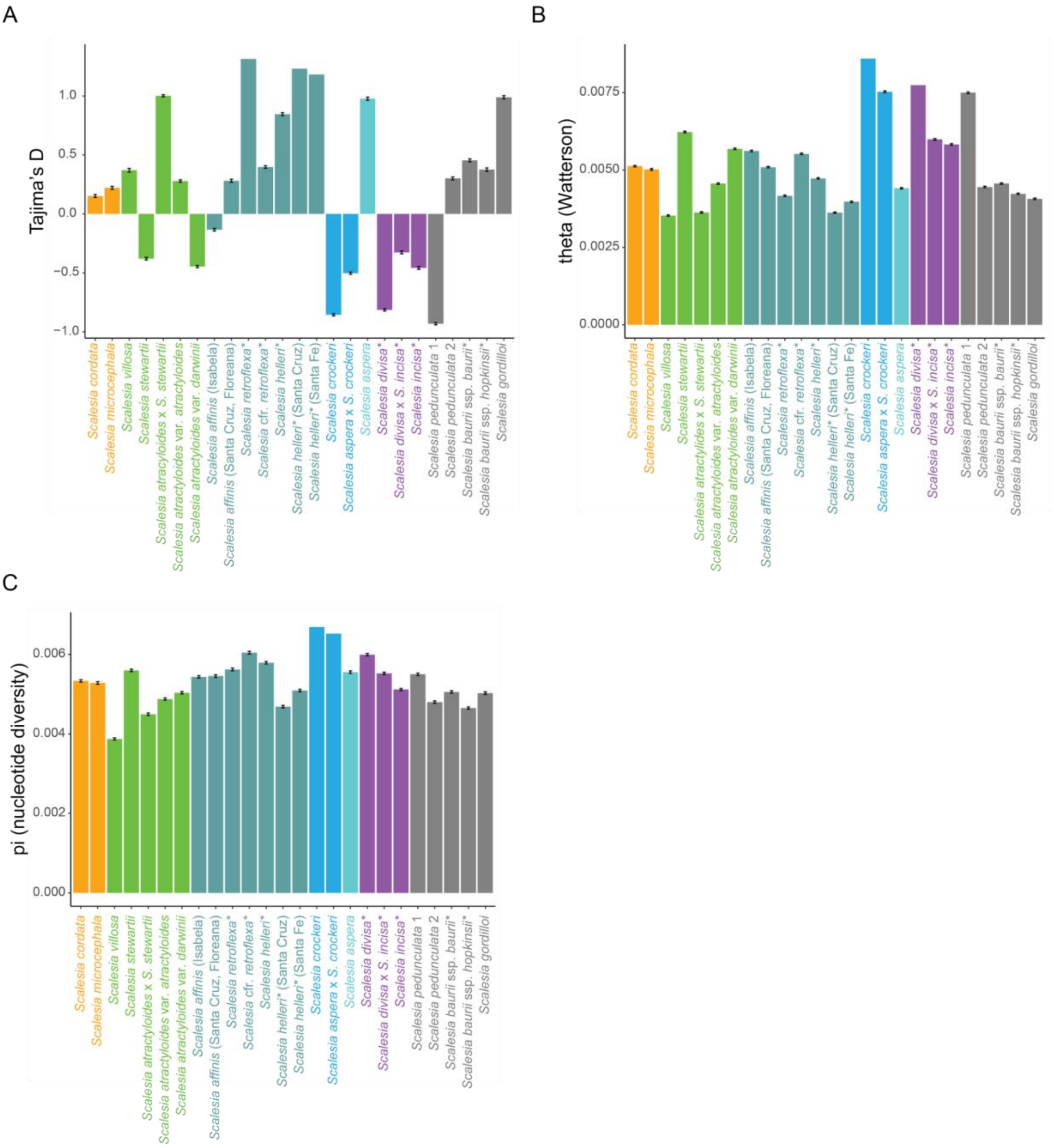

Fig S10. A: Tajima's D. B: Top right: theta (Watterson). C: Nucleotide diversity (theta pairwise, pi). Height of the bars indicate the value of the estimators. Bars are colored based on clade. Error bars indicate the 95% CI based on 1000 bootstraps. Species with lobed leaves are marked with an asterisk.

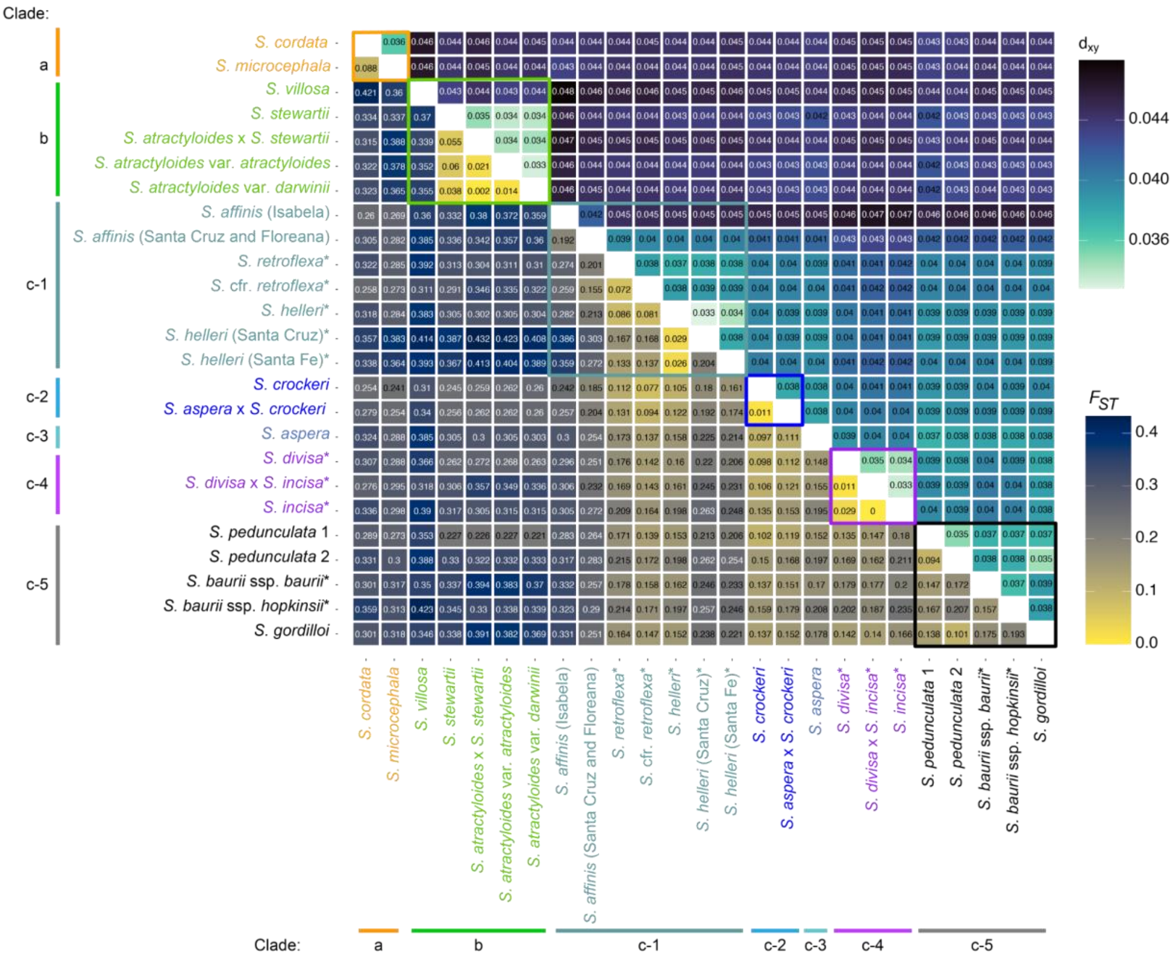

Figure S2. Divergence between species pairs. Upper diagonale shows d_{xy} at 4-fold degenerate sites and lower diagonale shows genome-wide F_{ST} estimates. Species names are colored according to their clades/subclades (see Fig. 1A). Coloured boxes are drawn around comparisons within the same clade/subclade. Tiles are colored according to the F_{ST} or d_{xy} value with yellow or light green being low and dark blue being high values. Species with lobed leaves are marked with an asterisk.

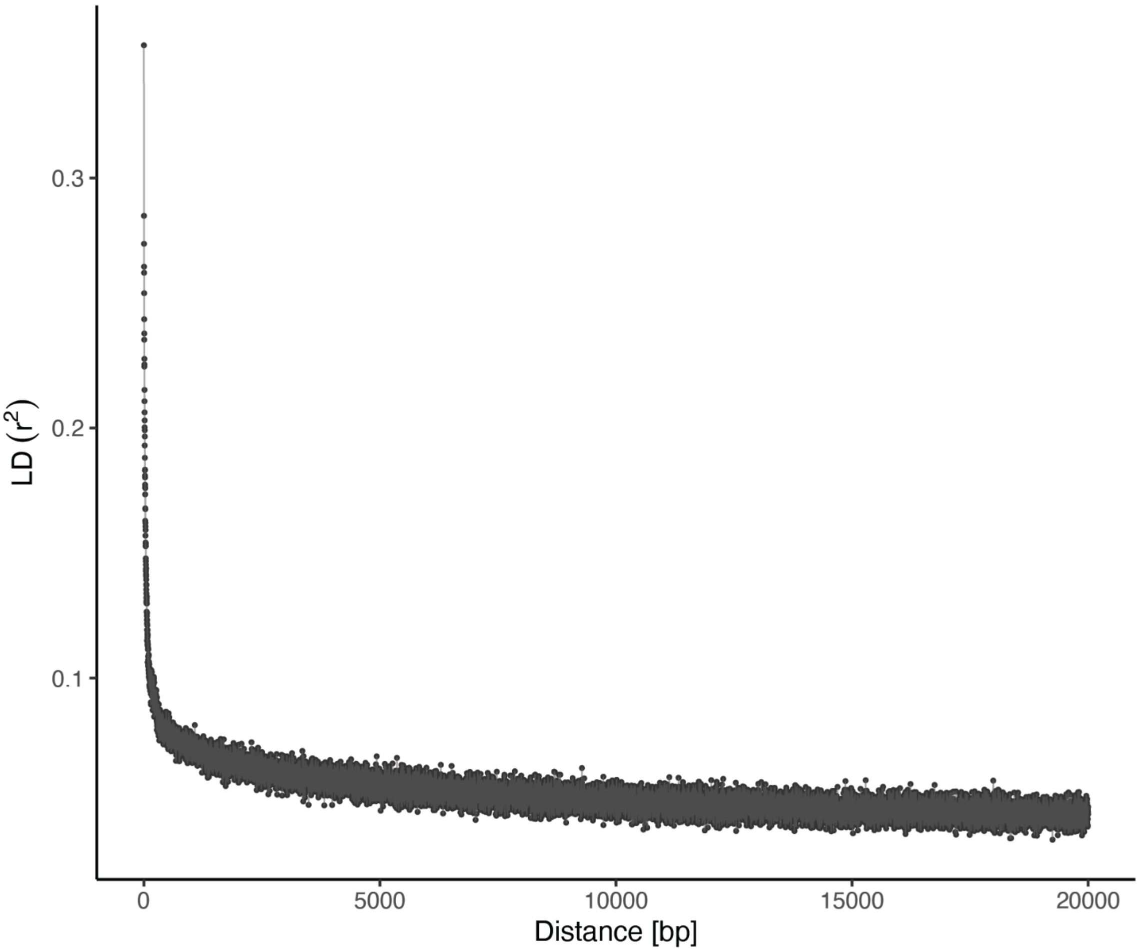

Fig. S30. Linkage disequilibrium (LD) versus genomic distance. The mean value for each distance is plotted.

R2-3: The paper does not clearly report the divergence (at silent sites, Ks) between the paralogous genes in Scalesia subgenomes – please report it! From the previous paper (Cerca et al 2022) it looks like polyploidisation is quite recent, ~4My, so I guess Ks between paralogs is only few percent. This means that distinguishing between these paralogs in read mapping and SNP calling is not easy, which is a major worry for me (and future readers) about the accuracy of SNP calling and possible artefacts originating from the mis-mapped reads between very similar paralogous copies of a gene. Such artefactual SNPs due to read mis-mapping would be expected to be present at high frequency, which may cause various biases and false signals, such as of population expansion in the past (peaks seen on FigS3). The problems with population genetic analyses of recently duplicated genes (as is the case in Scalesia) are common and not easy to resolve. The authors need to do a much better job with convincing the reader that they resolved these problems. Simply stating that their mapping quality >30 is certainly not enough as sequence reads originating from two different low divergent copies of a paralogous pair can be nearly identical and indistinguishable from genuine polymorphisms originating from two alleles of the same gene.

Thank you for your comment as it has given us a chance to make some important clarifications. *Scalesia* is an allotetraploid group, and for the *Scalesia atractyloides* reference genome used in this analysis, both subgenomes are fully resolved. This means that each homeologous chromosome (e.g. 1 each coming from the 2 subgenomes) has its own gene annotation and is thus theoretically capable of being unambiguously aligned against by query reads, especially when considering the 2 subgenomes were contributed by 2 different species such that homeologous chromosomes are relatively divergent. Indeed, past work has determined these subgenomes are far from identical and even are characterized by several chromosomal rearrangements (Figure 2A and Figure 4 in Cerca et al. 2022).

As requested by the reviewer, we used wgd (https://github.com/heche-psb/wgd?tab=readme-ov-file) to estimate

K_S between orthologs in the two *Scalesia* subgenomes (see figure below). As the sequencing reads were simultaneously mapped against both subgenomes, reads originating from nearly identical paralogs would have multiple best hits and therefore a mapping quality of zero. To ensure these reads are indeed excluded from downstream analysis, our approach ignores all reads with a Phred-scaled mapping quality score less than 30 and in addition uses the *-uniqueOnly* flag in angsd, which requires all reads to have unique alignments for consideration during the downstream analyses. It should be noted that this is a common approach to treat allopolyploid species such as ours. See for example this review paper on polyploid analysis and a recent article analysing allotetraploid *Coffea arabica*:

- Phillips, A. R. 2024. Variant calling in polyploids for population and quantitative genetics. Applications in Plant Sciences 12(4): e11607. https://doi.org/10.1002/aps3.11607
- Salojärvi, J., Rambani, A., Yu, Z. et al. The genome and population genomics of allopolyploid Coffea arabica reveal the diversification history of modern coffee cultivars. Nat Genet 56, 721–731 (2024). https://doi.org/10.1038/s41588-024-01695-w

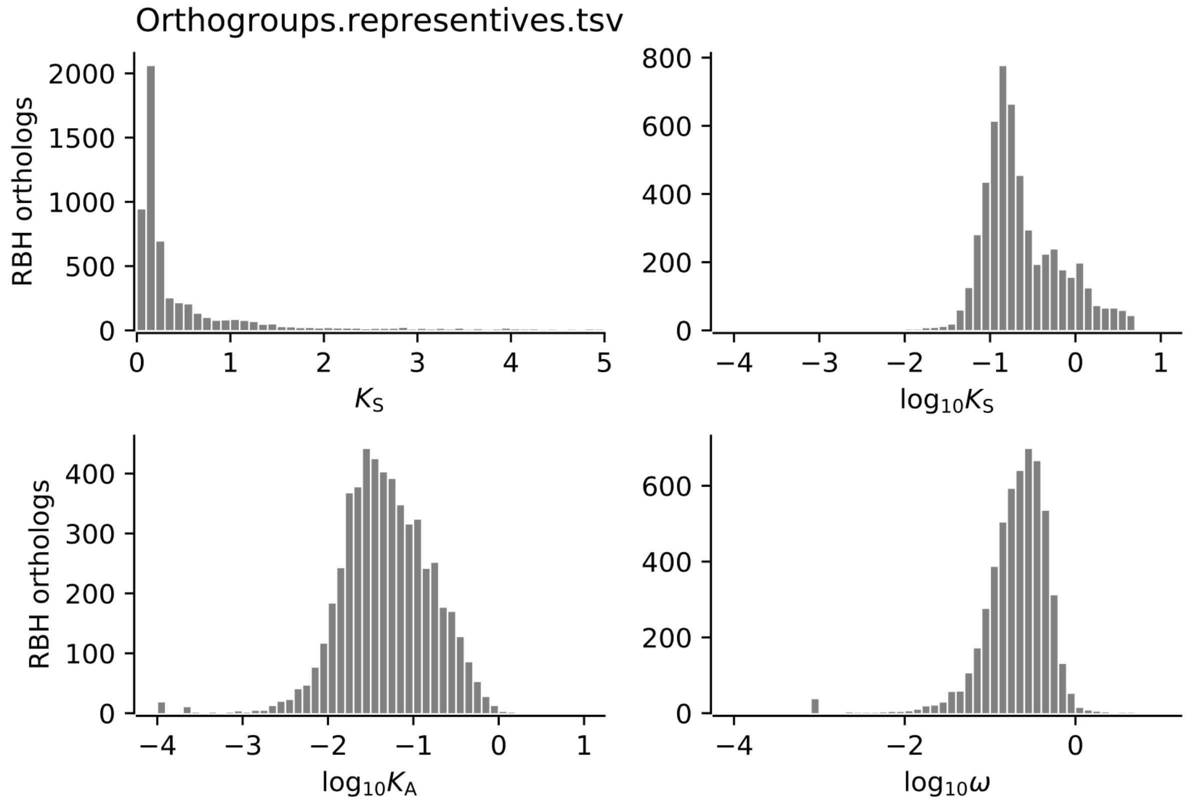

K_S analysis of orthogroup representatives from the two *Scalesia* subgenomes.

R2-4: Fig 1A. Scalebar for branch length is shown but figure legend does not explain what kind of distance is used in this tree.

Thank you for noticing and alerting us that we did not explain what the branch length represents. We the authors regret not initially including this important information about the *Scalesia* species tree reconstructed by ASTRAL under the multispecies coalescent model. We have revised the Figure 1A caption label. It now states: “Numbers on nodes indicate posterior probability and internal branch lengths are in coalescent units (N_e generations). Terminal branch lengths are set to 1 in the phylogeny …”

R2-5: Line 136 and elsewhere it is stated that samples were sequenced to “high-depth (>8X)”. Note that anything above 20X would be regarded as sufficiently high depth but 8X coverage is not at all “high depth”. Please correct this throughout the paper – avoid referring to low coverage (<10X) and medium coverage (10X<coverage<20X) samples as “high depth”.

Thank you for your comment. We note that the values for low coverage, medium coverage and high coverage are not universally defined and thus definitions vary between different publications. Given that the coverage of some samples is considerably higher than the mean value across all samples, we decided to call these “high-depth” and give our cut-off in parentheses. However, since this can lead to confusion, we have now changed it to “higher depth” across the manuscript. The sequencing depths achieved here are sufficient for our analyses and conclusions and reflect recent advancements in the power of evolutionary inference using genotype likelihoods rather than discretely determined genotypes.

R2-6: Lines 141-145: Based on observation of short internal and long external branches the authors infer “increased speciation rates and decreased extinction rates relative to Scalesia’s early evolution”. However, such ‘star-like’ tree may reflect recent population expansion in many Scalesia species. Recent population expansion is not detectable on PSMC plots (FigS3) because this method has poor resolution for recent events (e.g. fig1 in DOI: 10.1111/2041-210x.13740). Another potential (artefactual) cause of short internal and long external branches in a phylogeny is recombination that specifically creates this bias (see Schierup and Hein 2000 Genetics v156: 879-891). Recombination would be particularly problematic for multigenic phylogeny reconstructions as done in this manuscript.

Thank you for your comment. We have neglected to state in the figure caption that the terminal branch lengths were arbitrarily set to one coalescent unit as coalescence within individuals cannot be estimated without haplotype-resolved (i.e., phased) SNP calls. Thus due to this we believe the “long” terminal branches have been misinterpreted here. We have now corrected the figure caption. It should be noted that our Tajima’s D estimates (Figure S10, see **R2-3** response) show a mix of positive and negative values for species within clade *c*. Thus the “star-like” tree is unlikely to just reflect recent population expansion in all clade *c Scalesia* species. The potential bias due recombination seems to affect all ingroup species in the tree in a similar way in the publication cited by the reviewer. We thus think that such a bias would affect all clades in our phylogeny and not just clade c. Therefore, we argue that our interpretation of the phylogeny is valid.

R2-7: Lines 153-154: “using high depth (>12X, MAPQ30) samples”. Coverage and mapping quality varies across the SNPs. Perhaps “>12X, MAPQ30” refers to threshold used to filter SNPs before analysis rather than to samples? Please clarify.

This statement refers to the average genome-wide sequencing depth after filtering out reads with a mapping quality below 30. To clarify this, we have now changed it to “using higher-depth (>12X, after MAPQ30 filtering) samples” (l. 172).

R2-8: Lines 155-156: PSMC plots from FigS3 give information about population size change but not about the time of species divergence. Convergence of such plots at some point in the past for two species does not mean that the two species diverged at that time point. Time of pairwise species divergence has to be analysed properly with sequence divergence and population split models.

We do say that the similar demographic trajectories prior to ~1.4 Ma is “ indicating that they may not have yet begun to diversify into species”. We do not say that this is necessarily the case as we indeed did not perform a population split analysis. It should however be noted that other authors interpret past demographic estimates in a similar matter, e.g.:

- "Our results showed that the Ne curves of the four species converged back in time around 1–2 Ma, at Ne 200 000 and then started to diverge (Fig. 7). This is consistent with the existence of a common ancestor at this time, followed by rapid radiation and speciation." (Nadachowska-Brzyska et al., *PSMC analysis of effective population sizes in molecular ecology and its application to black-and-white Ficedula flycatchers*. Mol. Ecol. 25, 1058–1072 (2016).)
- "Results showed that all taxa had identical trajectories that included a decrease in Ne until ~3 MYA, followed by an increase and subsequent drop in Ne in a pattern unique to each taxon (Fig. 2C). This result suggested that all Hawaiian Metrosideros taxa share the same common ancestor that experienced a population bottleneck 3 to 4 MYA, which is likely when the ancestral population initially colonized the islands" (Choi, J. Y. et al. *Ancestral polymorphisms shape the adaptive radiation of Metrosideros across the Hawaiian Islands*. Proc. Natl. Acad. Sci. U. S. A. 118, (2021))

Moreover, as we pointed out in the text (l. 176-177), the time when demographic trajectories of *Scalesia* species based on PSMC start to differ from one another is consistent with a previous phylogeny-based estimate of *Scalesia* diversification. We thus think that our interpretation of the results are valid, although further work with

population split models would likely yield more accurate estimates of the divergence times of various Scalesia species pairs.

R2-9: Lines 218-219: Fay and Wu's H (and other tests of neutrality) do not provide "evidence for selection". They only show that the null model poorly fits the data. There are many possible causes for a test of neutrality to reject null model and it does not necessarily mean that selection caused the observed signal.

The Fay and Wu's H test was developed specifically to detect an excess of rare variants as a signature of genetic hitchhiking, defined as a change in allele frequency due to linkage with another allele undergoing a selective sweep. Indeed the original study reporting the H test (https://doi.org/10.1093/genetics/155.3.1405), entitled "Hitchhiking Under Positive Darwinian Selection", Fay and Wu motivate the development of the H test saying that "the spread of a positively selected mutation through a population removes linked neutral variation … However, there are a number of alternative explanations for reduced levels of variation … Distinguishing positive selection from these alternative explanations has become a significant empirical and theoretical pursuit." The authors then apply the H test to *Drosophila* sequences to identify "an excess of high frequency variants that are likely to have been influenced by positive selection". In our study we seek to make similar connections between positive selection and the H test, and indeed go further by combining the negative H values in combination with a further requirement that allele frequencies in the region must be highly differentiated (e.g. must be high-Fst outliers). This approach requiring multiple forms of evidence of positive selection (including Fay and Wu's H) was successfully used in several previously published studies, e.g.: (1) https://www.science.org/doi/10.1126/sciadv.abo5115, (2) https://doi.org/10.1111/mec.17274, (3) https://doi.org/10.1093/molbev/msz149, and (4) https://doi.org/10.1111/jse.12817. However, we have now added "putative" before "selected genes" throughout the manuscript as suggested in **R2-10** to clarify that processes other than selection potentially could lead to similar patterns.

R2-10: Line 224 and elsewhere in the paper. Please add "putative" before "selected gene". The preceding paragraph explains why none of the identified candidate genes are proven to be under selection and the authors have to be careful about this.

We have changed this as suggested by the reviewer.

R2-11: Lines 239-241: Unclear why the authors conclude that different species use different genes to evolve serrated leaves (do a classic genetic complementation test? – cross two serrated species; if diff mutations are involved then the progeny should have non-dissected leaf). The signals of 'selection' (i.e. various outliers in Fst and FayWuH) they detect are not necessarily caused by leaf shape evolution – there are loads of various factors (e.g. adaptation to new strains of viruses or parasites) that could have driven adaptation.

We thank the reviewer for their suggestion to experimentally test our conclusions. However, these experiments would require significant time and effort that are outside the scope of this study, e.g. research permits to do such work and access to germplasm of potential parents. Moreovers, very few of the species are known to be able to hybridize. In addition, a classic genetic complementation test can only be used when different homozygous recessive mutations are used by the different species/populations. In case of dominant alleles, the offspring will not necessarily have the wild-type phenotype (in this case unlobed leaves) even though different genes are used to create the mutant phenotype in the different species/populations.

In the previous paragraph we explicitly present the evidence for this statement: "Most (56%) of the 223 selected leaf genes are only found in one lobed species, and no gene is shared by all lobed species (Fig. 2C)." Thus 'most' of the leaf genes we identify as likely involved with the lobed phenotype are putatively subject to selection in only one species, and 'different species mostly use different genes'. We only looked for signals of selection within leaf development genes as these are less likely to be affected by adaptation to other factors such as adaptation to parasites.

R2-12: Line 319: Please add "some" before "leaf development genes" and add "likely" before "have been"

Thank you for your comment. We have changed this now as suggested.

R2-13: Line 321: How did the authors come to the conclusion that most adaptation occurred from standing variation?? Adaptation from standing variation leads to soft sweeps that are very difficult to detect. I did not see any convincing demonstration of adaptation from standing variation in the paper.

Thank you for presenting the opportunity to clarify this part of our work. In the opinion of us the authors, the manuscript states this very directly: “Within each lobed species and clade a and b Scalesia species, we evaluated whether SNPs within putatively selected leaf genes were ancestral (i.e. the allele was already present in early-diverging Scalesia genomes) or generated de-novo. We found that most of the SNPs were fixed for an ancestral allele in lobed species. However, SNP positions that are variable within a given lobed species are mainly de-novo mutations (Fig. 2B, Fig. S20). This indicates that selection on standing genetic variation is the primary process driving evolution of the lobed phenotype.”

While standing variation can be difficult to detect, we have in this study applied a rather sophisticated test to detect it: we used population genomic approach to identify selection on leaf-development genes, requiring windows containing these genes to be characterized both by extreme Fst outlier values and by negative values of Fay and Wu’s H. We further use comparative phylogenetics to identify whether SNPs within these genes were already present in unlobed, early-diverging Scalesia genomes, or rather whether these variants evolved de-novo in the lobed lineages. We found that most of the variants of interest were already present in early-diverging Scalesia genomes. We refer to this variation as “ancestral”, and as they are shown phylogenetically to be alleles that existed before the lobed phenotype evolved, we choose to refer to it as “standing variation”, e.g. “where the selected variant predates the selective pressure” as defined in Peter et al. 2012, PLoS Genetics (10.1371/journal.pgen.1003011). An approach similar to ours was used in this recent publication: J.Y. Choi et al., Ancestral polymorphisms shape the adaptive radiation of Metrosideros across the Hawaiian Islands, Proc. Natl. Acad. Sci. U.S.A. 118 (37) e2023801118, https://doi.org/10.1073/pnas.2023801118 (2021). However, if there is apreference for us to simply refer to this as ancestral variation, or by some other label, we are willing to do so.

R2-14: Line 331: Please remove “unprecedented”. Is this really unprecedented? Better avoid such poor journalistic stile in a scientific paper.

We have removed the “unprecedented” as requested.

R2-15: FigS3 legend. “Demographic reconstructions from past population sizes” please replace “from” with “of”. Also, please add to fig legend what approach was used – PSMC, MSMC2 or something else.

Thank you for noting the mistake and the missing approach. We have now changed the relevant text as suggested and added to the legend that PSMC was used.

Reviewer #3 (Remarks to the Author):

The authors present a genomic study of the Asteraceae genus Scalesia, which includes morphologically diverse species that inhabit various islands of the Galapagos. Following from a previous study that sequenced the genome of one species, S. atractyloides, the authors utilize this resource to investigate the potential adaptive variation in leaf lobation in Scalesia species. They conduct shallow genome sequencing for all species, with multiple populations represented, conduct a morphometric study of leaf characters for the species, and undertake a leaf transcriptomic analysis of selected species. The paper overall is generally well written, but as there is a lot going on in the paper, I think it could be better structured to help the reader understand why certain approaches were undertaken. There are also several aspects that need to be further clarified given the genomic complexities of this system.

R3-1: Line 73 – recommend some other word than crucible: location, region, etc.

We have replaced the word “crucible” with “hotspot”.

R3-2: Line 125 – Overall, the introduction should introduce the potential significance of leaf lobation better – as is found in the supplementary information. Many plants show flexibility in leaf lobation so it is unclear how this is perceived as an adaptive trait (leading to higher fitness, etc.).

Thank you for this helpful comment. We have now expanded the relevant section of the Introduction and added more references that support the well developed idea that leaf lobing can be an adaptive trait in environments that are hot and dry (l. 115-122). These are the same environmental factors that have been correlated with the occurrence of leaf lobing in *Scalesia*. However, since the possible defensive advantage of lobed leaves against flightless browsers such as land iguanas and giant tortoises is highly speculative and more data and experiments are needed to verify this hypothesis, we decided to not include it in the Introduction.

R3-3: Lines 142 – 145 ‘…clade c, which includes all species with lobed leaves…’ This phrasing is misleading, as this clade also includes unlobed species. Could these shorter branch lengths at the base of clade c relate to the species geography and island geology rather than leaf lobing as stated (Line 145)?

Thank you for spotting this misleading phrasing. We now included “among unlobed species” to avoid confusion. The sentence now reads: “[...] clade c, which includes, among unlobed species, all species with lobed leaves” (l. 158).

R3-4: Line 383 – How much data were generated for each sample? This information is indicated for the transcriptome analyses (line 601) but not the genome sequencing.

We are sorry for neglecting to report the total amount of DNA sequencing data generated per sample. In summary, we generated an average of 35.5 Gbp per sample (min: 3.1 Gbp, max: 219 Gbp, median: 30.9 Gbp). We have now added this information to the methods (l. 406-408).

R3-5: Given that these are all polyploid species (n=34 according to the Chromosome counts database), how were homeologous sequences dealt with for the analyses? There is one brief mention about the subgenomes (line 503) for SNP discovery. How were homeologs vs paralogs dealt with for the phylogenetic analyses and the population genetic analyses? Can you be certain that you are analyzing gene variation vs allelic variation among all the samples?

Thank you for your comment, as it enables us to clarify an important point that was neglected in the previous version of our manuscript: *Scalesia* is an allotetraploid genus as shown in Cerca et al. (2022) (https://doi.org/10.1038/s41467-022-31280-w). Thus the reference genome used in our study is fully resolved and contains both subgenomes, with the two subgenomes showing chromosomal rearrangements (Fig. 2A and Fig. 4 in Cerca et al., 2022). Furthermore, the genome assembly is of extremely high quality, with both haplotypes of each chromosome being fully resolved (number of chromosomes in the reference genome is 34, corresponding to n=34). Indeed upon publication, that assembly was the most complete and contiguous assembly from a large non-crop, non-model plant genome. We used that assembly as a mapping and gene annotation reference, thus we are confident that the vast majority of paralogs were properly assembled and thus are possible to distinguish in the mapping steps, as reads that have multiple best hits in the mapping to the reference genome were

removed from downstream analysis. Indeed the Cerca et al. (2022) study includes some analysis of paralogs and uses this information to discriminate between the two subgenomes. With both subgenomes fully resolved, mapping can be performed against them simultaneously and treated as effectively diploid in downstream analysis (Phillips, A. R. 2024. Variant calling in polyploids for population and quantitative genetics. Applications in Plant Sciences 12(4): e11607. https://doi.org/10.1002/aps3.11607). Please also see response **R2-3** for a response to a similar comment. We have added a brief summary of this in the methods:

"*Scalesia is an allotetraploid genus with the two subgenomes being fully resolved in the reference genome used for mapping*[42]*. Thus, it was effectively treated as diploid in downstream analysis as is common for polyploids with fully resolved subgenomes*[69]." (l. 410-412)

R3-6: Line 375 – change 'were' to 'was' at the end of the line

Thank you for noting this. We have corrected it to "was" now (l. 398).

R3-7: Line 440 – define SFS
Thank you for notifying us that we missed to define that. We now include site frequency spectra (SFS) in l. 488.

R3-8: Line 496 – define LTR
We have now included a definition and corrected "LTR" to "LRT" in l. 549.

R3-9: Line 542 – move once after conducted on its first use

Thank you for spotting this mistake. We have corrected it now.

R3-10: Lines 915-921. Figure 1 – add asterisks to taxon names in A (phylogeny). It would be helpful to add asterisks to lobed species names in all of the figures. Delete 'are' in line 917. The symbols in C are really difficult to distinguish.

Thank you for spotting the missing asterisks in Figure 1A. We have corrected this now. We have also added this to other figures as suggested. We agree that the symbols in Figure 1C are difficult to distinguish but have not found a better solution as the number of species is too high to use different colors for all of them. We instead now include population location maps per species in the SI (Figure S2-S8). We have changed the caption for Figure 1. It now reads:

"Numbers on nodes indicate posterior probability and internal branch lengths are in coalescent units (N_e generations). Terminal branch lengths are set to 1 in the phylogeny and tips are labeled with species names and identifiers for the individual plants, with island names given in parentheses."

Point by point response to REVIEWER COMMENTS

We thank the reviewers for their time and effort in reviewing our manuscript. Please find below point-by-point responses to the comments. Changes in the manuscript are highlighted in purple.

Reviewer #1 (Remarks to the Author):

I was Reviewer #1 in the previous review of this manuscript and am completely satisfied with the responses to my review by the authors and with their revision of the manuscript, taking into account their responses to the other two reviewers, as well. I have nothing further to suggest toward revision of the manuscript, which I believe is in fine shape.
Bruce G. Baldwin

Reviewer #1 (Remarks on code availability):

It appears that the authors have provided what is necessary at DRYAD.

We thank the reviewer for his time in assessing the revised manuscript. We greatly appreciate the positive feedback.

Reviewer #2 (Remarks to the Author):

The paper is somewhat improved after revision, but my concerns about selection analyses remain. The scan for selection used in this paper is relatively basic and it is prone to false positives due to sensitivity of Fay&Wu's H to by mis-identification of ancestral SNP state, as explained in the original Fay and Wu's paper (2020). There are many other approaches proposed more recently, such as Selscan (10.1093/bioinformatics/btae006), SweepFinder (10.1093/bioinformatics/btw051) and Raisd (10.1038/s42003-018-0085-8); at least one of these has to be added to the paper to make the analyses of selection more convincing.

The analyses in our paper are based on relatively low-depth sequencing depth from a dataset containing hundreds of genomes from a non-model tetraploid plant with a large nuclear genome. Such data were very expensive to generate and have great power in that hundreds of individual genomes can be analyzed simultaneously. However, we must rely on genotype-likelihood-based methods that can take genotype uncertainties (and/or site frequency spectra) into account, and as such we must face limitations in that many standard approaches and novel methods for genomic inference are typically not suitable.

We investigated in good faith the software tools suggested by the reviewer and found that unfortunately none of them are well suited to our data. Selscan cannot handle any missing genotypes, which is impossible to achieve due to the relatively low-depth sequencing in our dataset. Sweepfinder2 is compatible with allele frequencies, but requires a taxon-specific recombination map, which is infeasible in our system due to the expense, time, and lack of

required labor for generating and using such information. RAiSD requires called genotypes from a population-level sample, which are inaccurate for low-depth samples such as ours.

We are aware that the selection scanning method we apply is rather basic, and as such we refer to these regions as “putatively selected”. We acknowledge this in the relevant section reporting the selection results. We have also now added the word “candidate” when mentioning the selected genes in order to qualify that this selection is not proven. We mention some of the limitations of our method, and also call for further work using high-depth genomic sequencing and laboratory-based functional validation of the genes we report.

I’m not convinced by their claim that adaptation occurs mainly from preexisting variation. Adaptation from preexisting variation should result in soft sweeps that are not easily detectable with such statistics as FayWuH. To assess the contribution of preexisting (standing) genetic variation to adaptation it is better to use the tests that were shown to be effective in detecting soft sweeps (e.g. 10.1534/genetics.118.301502). The (negative) peaks of FayWuH they see (Figs S14 to S23) are likely due to hard sweeps (FayWuH is more sensitive to such sweeps) driven by denovo mutations or variants introgressed from other species. In small populations, as is the case in Scalesia, rare introgression of adaptive alleles is expected to play larger role in adaptation compared to large population. Sweeps driven by introgressed alleles would also explain why in many cases putatively selected SNPs detected in Scalesia are present in the other species too. There are tests specifically designed to detect such sweeps due to introgressed alleles (e.g. Volcanofinder [Setter et al 2020 PlosG]). Adding this analysis to the paper would clarify the source of adaptive alleles contributing to adaptation in Scalesia.

We considered the reviewer’s arguments and have come to agree that our analysis on ancestral alleles is not entirely convincing as was presented. Thus we have thus decided to remove it from the manuscript. This involved entirely removing one paragraph of Results+Discussion, deleting a panel from Figure 2, and otherwise slightly adjusting the phrasing of some statements in the Abstract and Conclusion. We appreciate the reviewers’ efforts to improve our manuscript.

Minor points:

In response to my request to remove “high” for seq depth >8, the authors changed “high” to “higher” throughout the paper. I do no think this is a satisfactory change. “Higher” than what? For example, the 1st sentence in Fig1 legend now reads “Multi-species coalescent phylogeny based on higher sequencing depth samples (> 8X)”. Why “high” or “higher” is needed here and elsewhere in the paper in the context of read depth? Why not simply give the information about read depth and let the reader decide whether it is high or not?

We have now removed the phrasing “high/higher”.

Thanks for clarifying distance units for branch length in fig1A legend. One other thing that is missing from the legend is the clarification of what the vertical lines with a,b,c,c-1… labels mean in fig1A.

Thank you for spotting that the explanation for the vertical lines and labels was missing from the figure caption. We have added the missing information now: “Vertical bars and labels indicate clades (a, b, c) and subclades (c-1, c-2, c-3, c-4, c-5)” (l. 949-950)

For fig2C “subclades with green: clade c-1” I do not see any green bars or dots – only purple and various shades of grey in the pdf version I’ve got for review.

We regret that the color scheme presented as difficult to distinguish in Fig. 2C. Indeed the green (petrol) bars can be perceived as difficult to distinguish from the other colors. We have thus updated the color scheme of that panel to hopefully make the colors easier to distinguish (Fig 2B).

Line 164: “below 0.2 respectively 0.041” change to “below 0.2 and 0.041, respectively”

Thank you for your comment. We have corrected this now (l. 158-159).

Lines 165-166: “Such FST values are typically considered moderate to high for differentiated populations within species, indicating that individual Scalesia species have not yet managed to generate substantial genetic diversity despite their success in adapting to various environments.” This is a surprising statement. Fst tells us little about diversity within individual species. Please refer to pi and theta levels here to support the statement that species have low diversity. Also, why “yet”? It implies that diversity is increasing, but no evidence for such increase over time is reported in the paper. Small island populations are expected to have small Ne and low genetic diversity, so pi~0.5% (FigS10c) may actually be regarded as relatively high genetic diversity for small island populations.

Thank you for your comment. We actually intended to refer to "differentiation" rather than “diversity”, as F_{ST} between different Scalesia species is rather low given they are considered different species. Thus, the “yet” refers to the expectation of population/species differentiating when gene-flow is low and when they adapt to different environments. We have corrected the sentence, it now reads: “Such F_{ST} values are typically considered moderate to high for differentiated populations within species[43], indicating that individual *Scalesia* species have not yet managed to generate substantial genetic differentiation despite their success in adapting to various environments.” (l. 161)

Line 191: “Low gene flow … facilitated by their nearly allopatric distribution” please rephrase to make it clear that allopatry leads to reduction in gene flow rather that facilitates it.

Thank you for your comment. We have re-phrased that sentence now: “Low gene-flow between species even when occurring on the same island may be due to their nearly allopatric distribution in combination with poor adaptation for long-distance dispersal in *Scalesia*.” (l. 186)

Figs S14 to S23. It would be really helpful to see plots of pi for each of the compared species along with Fst and FayWuH plots that are already shown. Sweep in one species should reduce polymorphism in that species and it would be reassuring to see this along with plots of Pst and FayWuH.

We have added additional panels for pi to Figures S14-23 as suggested by the reviewer.

Line 463 and FigS30 legend. Please clarify which species was used for this analysis. Was it done for each of the species (then why a single LD decay plot shown)?

We apologize for the confusion here. We used all samples in that plot but we agree with the reviewer that this should be done per species. We have updated the relevant methods description and added additional SI figures per species (Figures S29-53).
